# Heterosis patterns and sources of self-compatibility, cross-compatibility and key nut traits within single and double hybrid crosses of kola [*Cola nitida* (Vent) Schott and Endl.]

**DOI:** 10.1038/s41598-023-30485-3

**Published:** 2023-05-17

**Authors:** Daniel Nyadanu, Samuel Tetteh Lowor, Prince Pobee, Jerome Agbesi Dogbatse, Abraham Akpertey, Micheal Brarko-Marfo

**Affiliations:** Cocoa Research Institute of Ghana, P. O. Box 8, Akim Tafo, Ghana

**Keywords:** Biochemistry, Genetics, Physiology, Plant sciences

## Abstract

Sexual incompatibility among kola genotypes accounted for over 50% yield loss. Compatible and high yielding varieties are in demand to develop commercial orchards. The objective of this study was to assess self-compatibility and cross-compatibility of kola (*C. nitida*) genotypes within self, single and double hybrid crosses and to determine heterosis pattern in the resulting hybrids for sexual compatibility and key nut yield and quality traits. Crosses among kola genotypes from three field gene banks (JX1, GX1, MX2) and one advanced germplasm (Bunso progeny) in Ghana were evaluated along their parents for sexual compatibility, nut yield and nut quality. Data were collected on pod set, pseudo-pod set, pod weight, number of nuts per pod, nut weight, brix, potential alcohol and nut firmness. Significant (P < 0.001) differential pod set was observed within Bunso progeny, JX1, GX1 and MX2 crosses; while pseudo-pod set differed only within JX1 and MX2 crosses (P < 0.001). Very large prevalence of mid-parent, heterobeltiosis, and economic heterosis was observed for sexual compatibility, outturn and brix for the single and double hybrid crosses. Heterosis was prominent among the double hybrid crosses as compared to the single hybrid crosses suggesting that recurrent selection of compatible varieties from advanced generations could result in genetic gain in kola improvement. The top five crosses with best heterosis for sexual compatibility and an appreciable positive heterosis for outturn and brix were B1/11 × B1/71 × B1/157 × B1/149, B1/11 × B1/71 × B1/296 × B1/177, GX1/46 × GX1/33 × B1/212 × B1/236, JX1/90 × JX1/51 and JX1/51 × JX1/36. These materials could serve as sources of beneficial alleles for improving Ghanaian kola hybrids and populations for yield and sexual compatibility.

## Introduction

The genus Cola (Malvaceae) is made up of evergreen tropical and sub-tropical fruit crops^1–3^. *Cola nitida* and *Cola acuminata,* native to the warm and humid regions of tropical West Africa^4^ and with chromosome numbers 2n = 40, are of commercial importance as both species are cultivated for their edible nuts (kola nuts)^5^.

Kola nuts have multiple uses^6,7^. Kola seed has nutritional, cultural, cosmetic and pharmaceutical interests^8,9^. Kola nuts are chewed to encourage salivation, to keep awake^10,11^ and for traditional rites like marriages and naming ceremonies^12–14^. Kola nuts are used in confectionary industries as active ingredients in beverages and pharmaceuticals^13,15^. Beverage companies use kola due to the high caffeine, 1.84–2.56%^7,16^ content of the nuts. Thus, kola nuts are greatly used for the manufacture of energy drinks^15,17^. Kola nuts are also major source of bioactive compounds and are regarded as the future gold mine of plant therapy^8,9,18–21^. The genus Cola has long been involved in Ayurvedic preparations which is based on the idea of herbal treatment and other natural therapies to treat various ailments and disorders. The genus has received the attention of pharmaceutical industries due to the presence of bioactive molecules like Hydroxy Citric Acid (HCA), oleic acid, flavonoids and theobromine which has immense remedial qualities^22,23^.The nuts are also rich in palmitic and oleic acids which are known to maintain good skin; consequently they are used in the production of cosmetic products such as soaps^24^, while the red coloured ones are regarded as a rich source of red pigments in the plant kingdom^25,26^. The pigments that give fresh foods their vibrant hues of red, green, purple, yellow and orange do more than just make a pretty meal, they contain powerful antioxidant properties that make a profound effect on the total health of consumers.

Ghana kola nut production has increased from 5000 tons in 1961 to 25,303 tons in 2019^27^. The uses of the crop have created an increased demand globally in excess of current production of the crop^24,28^. In the rural economy of Ghana, where an estimated 46% of the people of the country live, especially in the forest agro-ecological zones of the country, kola cultivation and marketing plays a key role in the sustenance of livelihoods^29,30^. Kola contributes significantly to the foreign revenue of Ghana. The Gross Domestic Product for per capita contribution of kola to the economy of Ghana was US$1,370 and Gross Domestic Product in exchange rates was US$37,543,361,204^31^. This highlights the economic potential of kola as a cash crop.

Despite the cultural, nutraceutical and economic importance of kola, breeding efforts in kola are limited. Kola production has been based primarily on selections from the wild over a long history. The current exploitation of the species in Ghana mainly relies on just few stands in farmers’ background, home garden and farms^9^. In addition, only few plantations/ orchards of the species are available. These observations suggested that the species in its current state is unlikely to meet the continuously growing local and international demands. Exploiting options for large-scale cultivation requires availability of improved planting materials which farmers have indicated as one of their challenges/needs^14^. Although few cultivars have been selected from open-pollinated seedlings over the years, breeding programs to develop new and improved cultivars were not established until 1967 when active kola research began at the Cocoa Research Institute of Ghana (CRIG) with introduction of some kola genotypes from Oyoko in the Eastern region of Ghana^32^. Two high yielding hybrids GX1/46 × GX1/16 and JX1/5 × JX1/9 were recently recommended to farmers by the Cocoa Research Institute of Ghana^33^.

Management techniques in commercial orchards have been improved remarkably in recent years, but genetic improvement have not kept pace with the large economic value of this important crop. Besides, there is a need of improved varieties to overcome self- and cross-incompatibility, and to resist drought stress and kola weevils (*Balanogastris kolae* and *Sorphorhinus* spp), which are among the major constraints of kola value chain^24,34,35^.

Kola is an allogamous plant due to self-incompatibility (SI)^36^. SI is a genetically controlled mechanism that prevents self-fertilization in about half of angiosperm species^37^, and stands as one of the most effective systems adopted by flowering plants to prevent inbreeding and maintain a high level of diversity^38,39^. SI is generally subdivided into two distinct groups; sporophytic incompatibility in which the incompatible phenotype in the pollen is determined by the genotype of the pollen-producing plant and gametophytic incompatibility where the genotype of the individual microspore determines the phenotype of the pollen^40^. Kola cultivars express sporophytic self-incompatibility and are also cross-incompatible in many combinations^41^. Oladokun^42^ attributed over 50% of low yield in kola to sexual incompatibility among genotypes. Incompatibility restricts the number of desirable crosses that can be made and dictates their direction in many breeding programmes^43,44^. Furthermore, in commercial kola orchards, one or more pollinizers must be included to ensure good pod set on the main cultivar.

Knowledge of compatible and high yielding varieties are critical for the development of high yielding commercial hybrids of kola. Therefore, the availability of information on the compatible partners and patterns of compatibility of lines in the breeding programme among the several valuable kola cultivars collected and conserved at the Cocoa Research Institute of Ghana (CRIG) would boost the national kola production system. A clear definition of the most successful self-compatible and cross-compatible individuals or genotypes would improve fruit set and productivity.

Fruit set assessment is currently the most used method to test self and cross-compatibility among cultivars^45–48^. Furthermore, developing improved varieties with enhanced nut quality attributes ensures consumer acceptability of the end products^49–52^. Aside fruit set, it is important to select cultivars with enhanced nut quality attributes such as brix, texture or firmness of nuts, and potential alcohol content. Knowledge on correlation between fruit set and these quality traits in kola is lacking. Correlation strength among traits is important in cultivar development. It provides valuable information that aid breeders in determining the most efficient design for genotype evaluations^34^.

Plant breeding is a long process requiring efficient selection of suitable parents with desired traits to produce superior hybrids. Sexual compatibility in kola is determined to a larger extent by non-additive gene effects than by additive genetic effects^45^. Dominance variance is thus very important in selection for pod set/compatibility in kola. Breeding for expression of dominance is accomplished by the selection of parental pairs that express higher than average for the trait of interest when crossed, that is, heterosis breeding. Utilization of heterosis can speed up the process of generating superior hybrids^53^. Development of locally preferred hybrids with certain fruit characters along with high yield and adaptation is essentially achieved through heterosis breeding^54^.

Heterosis or hybrid vigour is a natural phenomenon whereby hybrid offspring of genetically diverse individuals display superior performance relative to the mid-parent value (average heterosis), or to the superior parent (heterobeltiosis) or over check/released cultivar or variety (economic heterosis)^55^. Recent advancements in molecular genetics have confirmed that the cause of heterosis is purely genetic^53^. Heterosis breeding involves evaluation of elite-parents and first filial generations to detect heterotic hybrids and appropriate parents^56^ for key traits of crops. Despite the established application of heterosis in plant breeding to identify outstanding genotypes or parents, not much is known about its use in selecting superior parents and hybrids for sexual compatibility and yield in kola.

Therefore, the objective of this study was to assess self-compatible and compatible partners of kola (*C. nitida*) genotypes from self, single and double hybrid crosses and heterosis pattern in resulting hybrids for sexual compatibility and key nut yield and quality traits.

## Results

### Variation in sexual compatibility among the BUNSO progeny crosses

**Figure 1:**
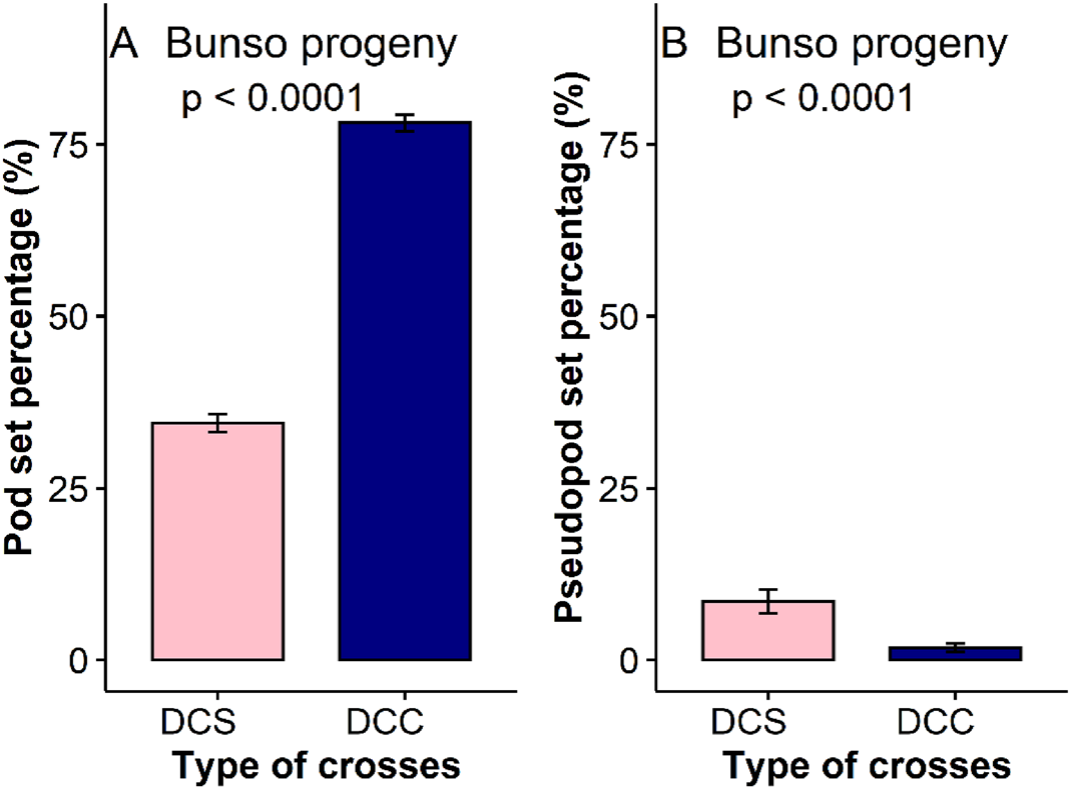
Comparative analysis of pod set and pseudo-pod set for double crosses and double self- crosses. *DCS* Double hybrid self-crosses, *DCC* Double hybrid crosses, *A* Variation among DCS and DCC for pod set (%), *B* Variation among DCS and DCC for pseudo pod set (%) Bunso progeny.

There were significant differences (P < 0.001; df = 83) among the double hybrid crosses for pod set (Supplementary Table S1). Variability among the crosses for pseudo pod set was however not significant (P > 0.05; df = 83). Pod set ranged from 21.3% in DCS14 to 97.0% in DCC19 (Supplementary Table S1). Pseudo-pod set ranged from 0.0 in several crosses to 25.4% in DCC5 (Supplementary Table S1). Pod set was more than two-fold higher in the double hybrid crosses (DCC) as compared to the double hybrid selfs (DCS). By contrast, pseudo-pod set was significantly (P < 0.0001) higher in the DCS as compared with the DCC (Fig. 1). The higher pseudo pod sets among self-crosses than crosses between different genotypes suggests a linkage between pseudo-pod set and self-incompatibility in kola.

### Variation in sexual compatibility among the AFOSU JX1 crosses

Variation among the single crosses of some JX1 accessions for the percentage pod set and percentage pseudo-pod set was significant (P < 0.001; df = 141) (Supplementary Table S2). Single self-crosses including JX1/11 × JX1/11, JX1/122 × JX1/122, JX1/23 × JX1/23, JX1/49 × JX1/49 and JX1/51 × JX1/51, and single hybrid crosses including JX1/73 × JX1/36, JX1/87 × JX1/118 were incompatible, validating the phenomenon of self-incompatibility and some degree of cross-incompatibility in kola. Pod set of single crosses including JX1/112 × JX1/23, JX1/23 × JX1/34, JX1/6 × JX1/27, and JX1/J1 × JX1/66 were very low suggesting non-compatibility of these partners. The compatibility and fertility of the combinations JX1/1 × JX1/67, JX1/20 × JX1/118, JX1/20 × JX1/31, JX1/21 × JX1/9, JX1/2 × JX1/45, JX1/51 × JX1/23, JX1/51 × JX1/20, JX1/62 × JX1/54, JX1/63 × JX1/23, JX1/73 × JX1/6, JX1/8 × JX1/119 and JX1/J1 × JX1/23 were higher than other crosses combinations (Supplementary Table S2). They are therefore good materials for involvement in kola breeding programmes to increase genetic gain for compatibility and yield in improved varieties.

**Figure 2:**
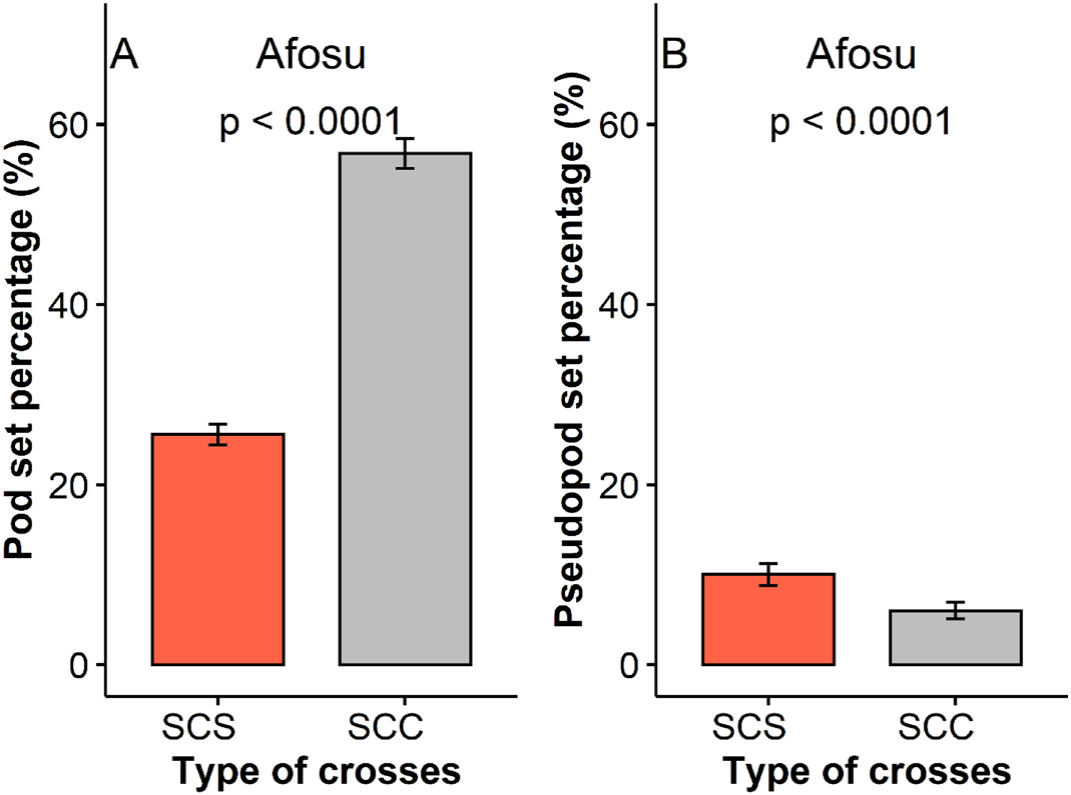
Comparative analysis of pod set and pseudo-pod set for single crosses and single self- crosses. *SCS* Single hybrid self crosses, *SCC* Single hybrid crosses, *A* Variation among SCS and SCC for pod set (%), *B* Variation among SCS and SCC for pseudo pod set (%).

Pod set of the single hybrid crosses (SCC) of JX1 was more than two-fold higher (P < 0.001) than pod set of single hybrid selfs of JX1 germplasm collection (Fig. 2). Pseudo pod set was observed to be significantly (P < 0.0001) higher for single hybrid selfs of JX1 as compared to single hybrid crosses (Fig. 2).

### Variation in sexual compatibility among the AFOSU GX1 self-crosses

There was significant (P < 0.001; df = 27) variation among the single hybrid selfs of GX1 for pod set, which ranged from 1.9% for GX1/25 × GX1/25 to 54.3% for GX1/87 × GX1/87 (Table 1). Majority of the GX1 self-crosses expressed very low and low compatibility. However, GX1/16 × GX1/16, GX1/27 × GX1/27, GX1/30 × GX1/30, GX1/37 × GX1/37, GX1/50 × GX1/50 and GX1/87 × GX1/87 were moderately compatible. These genotypes could be sources of self-compatibility genes for development of self-compatible varieties. Variability in pseudo-pod set among the GX1 self-crosses was not significant (P > 0.05) (Table 1). GX1/16 × GX1/16 did not express pseudo-pod set. Pseudo-pod set was more observed for GX1/1 × GX1/1, GX1/24 × GX1/24, and GX1/3 × GX1/3 among the crosses (Table 1).

**Table 1:** Variation in pod set, yield and nut quality traits among GX1 self-crosses.

Code	Crosses	PS	PSP	NP	PW	NNP	WUN	WPN	OT	Brix	PA	FN
SCS29	GX1/1 × GX1/1	35.1	14.0	15.0	159.5	8.8	88.7	61.7	69.1	15.1	6.5	13.2
SCS28	GX1/16 × GX1/16	48.6	0.0	20.0	210.8	12.1	122.5	81.4	70.0	9.8	6.6	12.1
SCS35	GX1/2 × GX1/2	5.4	3.9	2.4	25.6	1.6	16.7	11.2	46.4	5.6	4.0	10.1
SCS30	GX1/21 × GX1/21	34.1	6.9	12.0	125.8	7.5	75.9	53.7	70.3	7.7	7.3	13.5
SCS31	GX1/24 × GX1/24	26.5	20.3	10.0	104.6	6.5	66.5	49.3	75.7	13.5	7.9	16.7
SCS32	GX1/25 × GX1/25	1.9	1.9	0.9	8.6	0.6	5.7	5.1	29.8	3.4	3.1	5.2
SCS33	GX1/27 × GX1/27	51.0	15.2	19.7	206.8	12.2	124.5	79.7	67.3	12.8	5.7	13.3
SCS34	GX1/29 × GX1/29	26.4	5.9	10.8	112.9	6.3	63.0	50.5	79.3	9.9	6.9	11.5
SCS55	GX1/3 × GX1/3	5.9	18.4	2.6	27.7	1.6	15.9	11.0	69.9	11.5	8.2	13.6
SCS36	GX1/30 × GX1/30	50.9	5.1	25.7	270.4	13.7	139.5	79.3	62.1	11.3	8.3	12.9
SCS37	GX1/35 × GX1/35	15.1	6.7	5.9	61.8	3.8	38.3	23.9	66.2	12.0	7.1	14.4
SCS38	GX1/36 × GX1/36	32.6	10.0	13.8	144.9	8.5	86.2	70.7	81.7	17.0	7.3	14.8
SCS39	GX1/37 × GX1/37	47.1	6.8	20.8	217.4	12.8	129.5	82.2	64.6	14.9	8.0	12.9
SCS40	GX1/38 × GX1/38	11.8	8.7	4.4	46.8	2.6	26.2	17.9	69.7	14.6	7.2	15.4
SCS41	GX1/4 × GX1/4	40.0	7.8	17.3	181.2	10.6	107.8	72.8	69.2	13.2	6.6	14.7
SCS42	GX1/50 × GX1/50	41.3	13.5	16.9	178.8	9.9	99.4	78.3	78.5	14.0	6.9	15.1
SCS43	GX1/53 × GX1/53	6.8	7.5	3.5	38.2	2.0	20.4	12.9	43.5	5.7	4.0	9.6
SCS44	GX1/60 × GX1/60	35.8	3.9	15.7	165.8	8.7	86.4	71.8	82.4	11.5	8.1	14.3
SCS45	GX1/64 × GX1/64	33.3	6.7	17.0	189.6	6.3	56.3	41.3	73.3	12.9	5.6	16.4
SCS46	GX1/74 × GX1/74	31.2	7.1	10.3	103.2	7.8	81.7	60.8	74.1	11.7	8.8	13.8
SCS50	GX1/7 × GX1/7	29.5	9.1	11.2	117.2	6.9	70.2	56.0	81.4	14.0	5.5	15.3
SCS47	GX1/71 × GX1/71	12.0	3.9	5.3	55.4	3.2	31.9	23.7	76.4	8.4	8.0	15.7
SCS48	GX1/72 × GX1/72	4.2	2.1	2.0	21.2	1.0	9.5	7.9	55.3	11.5	5.0	10.6
SCS49	GX1/75 × GX1/75	28.3	13.3	11.8	123.7	7.5	76.7	59.3	78.3	13.0	7.7	14.0
SCS51	GX1/82 × GX1/82	26.6	16.0	12.6	133.9	7.2	72.0	53.9	74.1	14.7	7.7	15.2
SCS52	GX1/83 × GX1/83	31.1	12.5	12.7	132.3	8.1	82.4	58.7	69.7	11.0	7.7	13.7
SCS53	GX1/86 × GX1/86	23.0	2.2	9.0	93.8	5.3	52.8	41.0	77.3	13.7	7.8	16.0
SCS54	GX1/87 × GX1/87	54.3	6.1	21.7	228.0	13.2	134.0	74.6	58.4	10.5	7.3	16.1
	S.e Mean	4.8 28.0	8.0 8.5	3.2 11.7	35.5 123.1	2.0 7.0	21.9 71.0	15.7 49.7	19.2 68.2	5.0 11.57	2.5 6.8	4.5 13.5

*PS* Pod set (%), *PSP* Pseudo pod set (%), *NP* Number of pods, *PW* Pod weight, *NNP* Number of nuts/pod, *WUN (g)* Weight of unpeeled nuts (g), *OT* Outturn (%), *PA* potential alcohol, *FN* firmness of nuts (lb), *SCC* Single hybrid cross, *SCS* Single hybrid self-cross.

### Variation in sexual compatibility among the TAFO MX2 self-crosses

Variation among MX2 accessions for self-compatibility was significant (P < 0.0001; df = 26). The top five self-crosses that expressed significantly higher compatibility were JB1 × JB1, A1 × A1, JB 37 × JB 37, JB 36 × JB 36 and JB 9 × JB 9 (Table 2). Pod set ranged from 13.7% for Atta 3 × Atta 3 to 48.7% for JB1 × JB1. Most of the self-crosses expressed very low and low compatibility. A1 × A1 and JB1 × JB1 were moderately compatible and represent sources of self-compatibility among the MX2 genotypes. There was also significant variation among self-crosses of MX2 genotypes for pseudo-pod set. Pseudo-pod set for self-crosses ranged from 6.4% for Atta 1 × Atta 1 to 24.3% for W25 × W25 (Table 2). Genotypes A10, A1, A8, JB 36, JB 34, JB 35, JB 37, JB 40, JB 9 and P2-1c did not express pseudo-pod set when self-pollinated.

**Table 2:** Variation in pod set, yield and nut quality traits among MX2 self-crosses.

Crosses	PS	PSP	NP	PW	NNP	WUN	WPN	OT	Brix	PA	FN
A 10 × A 10	28.2	0.0	15.7	151.9	7.0	64.6	54.1	83.2	10.4	8.2	14.3
A1 × A 1	48.6	0.0	30.1	297.0	5.0	46.2	32.0	70.0	10.9	7.8	13.1
A12 × A 12	35.1	12.5	20.5	202.6	4.7	43.4	30.9	71.4	9.3	7.1	12.7
A2 × A2	28.1	11.0	18.2	179.3	6.7	60.9	47.6	78.6	7.7	6.1	15.0
A22 × A22	25.1	16.4	15.7	156.1	4.3	39.7	29.4	73.8	11.1	7.7	14.6
A26 × A26	29.5	19.0	16.8	166.0	4.7	42.4	33.2	78.2	13.4	6.4	15.0
A8 × A8	34.9	0.0	22.9	227.1	7.0	64.6	49.2	77.2	12.2	8.7	13.6
Atta 1 × Atta 1	27.3	6.4	15.8	156.6	5.0	45.2	33.9	74.9	15.2	10.5	14.7
Atta 3 × Atta 3	13.7	12.8	6.1	59.3	4.0	35.9	25.9	70.1	15.2	8.4	13.5
Club × Club	30.3	9.0	20.4	201.5	6.3	59.1	48.1	80.8	18.1	6.4	15.8
JB 1 × JB 1	48.7	0.0	27.7	274.3	6.3	59.1	47.1	79.5	15.7	7.0	14.4
JB 10 × JB 10	28.5	18.6	15.6	152.6	5.3	47.9	36.7	76.0	18.8	7.2	14.9
JB 15 × JB 15	21.7	0.0	10.9	106.5	6.3	59.1	48.4	81.3	10.2	6.5	15.7
JB 17 × JB 17	24.2	17.4	12.2	118.7	5.0	45.2	34.8	76.8	13.6	6.7	13.5
JB 19 × JB 19	15.3	20.9	9.7	93.1	4.7	42.4	33.6	79.0	18.8	7.6	16.1
JB 26 × JB 26	30.0	0.0	21.6	207.9	5.7	51.7	42.7	80.6	17.1	5.3	12.2
JB 3 × JB 3	22.8	16.6	13.3	131.0	6.3	58.1	46.9	80.6	16.7	4.5	15.7
JB 32 × JB 32	27.3	20.9	12.1	119.3	4.3	39.7	30.8	77.0	5.7	7.6	13.9
JB 35 × JB 35	18.7	0.0	12.1	116.4	6.0	56.4	45.8	79.4	6.1	7.8	12.9
JB 36 × JB 36	37.0	0.0	22.8	222.4	5.7	51.7	41.4	80.2	7.3	6.2	13.4
JB 37 × JB 37	41.9	0.0	24.1	234.6	5.7	52.6	42.0	78.5	12.9	4.8	14.1
JB 40 × JB 40	29.3	0.0	15.7	155.5	5.0	46.2	34.6	74.6	12.7	7.7	14.5
JB 9 × JB 9	36.4	0.0	19.3	189.2	7.0	64.6	52.6	81.5	11.0	9.5	13.0
JB34 × JB 34	33.5	0.0	21.7	208.4	6.7	60.9	49.7	81.5	9.0	8.8	15.6
P2-1B × P2-1B	26.0	21.8	14.5	140.3	6.7	62.9	50.9	79.2	17.9	7.5	16.7
P2-1c × P2-1c	34.0	0.0	20.6	201.4	4.7	43.4	31.7	73.0	12.6	6.6	16.2
W25 × W25	27.6	24.3	15.7	156.1	5.7	52.7	40.8	77.4	10.8	5.0	14.0
S.e Mean	8.5 29.8	5.5 8.4	5.6 17.5	55.5 171.3	1.1 5.6	10.5 51.7	10.2 40.6	5.9 77.6	0.7 12.6	0.3 7.2	1.6 14.4

*PS* Pod set (%), *PSP* Pseudo pod set (%), *NP* Number of pods, *NNP* Number of nuts/pod, *WUN (g)* Weight of unpeeled nuts (g), *OT* Outturn (%), *PA* potential alcohol, *FN* firmness of nuts (lb).

### Grouping of crosses into compatible classes

Among the double hybrid crosses, none of the crosses had compatible classes 0, 1 and 2. Double hybrid cross had a compatibility score ranging from 3 to 5. In contrast, double hybrid self-crosses exhibited compatibility scores ranging from 2 to 3. The single hybrid crosses were distributed among the compatibility scores of 0 to 5 with compatibility class 4 mostly expressed (χ^2^ = , df = 5, p < 0.001). On the other hand, single self-crosses had compatibility scores ranging from 0–4 and compatibility score 2 was predominant (χ^2^ = , df = 5, P < 0.001) (Supplementary Fig. S1). The cross-hybrids were more compatible than the self-hybrids for both the double hybrid crosses and single hybrid crosses.

### Variation in yield components and nut quality traits of the BUNSO progeny crosses

There were significant differences (P < 0.001; df = 83) among the Bunso progeny crosses in number of pods, pod weight, number of nuts/pod, weight of unpeeled nuts, weight of peeled nuts and percentage outturn (Supplementary Table S1). Number of pods ranged from 14.4 in DCS9 and DCS16 to 83.6 in DCC19 (Supplementary Table S1). Number of pods for some crosses (DCC6, DCC19, DCC24, DCC 26) was about two-fold higher than number of pods for crosses (DCC11, DCC13, DCC17, DCC38) and more than three-fold higher than those of crosses DCC5, DCS12, DCS14 (Supplementary Table S1). The significant variation in pod weight ranged from 62.8 for DCS9 and DCS16 to 364.2 for DCC19 (Supplementary Table S1). The top five crosses with higher pod weight included DCC19, DCC26, DCC24, DCC6 and DCS3 (Supplementary Table S1). Number of nuts per pod of crosses (DCC19 and DCC26) was more than three-fold higher than that of other crosses (DCC5, DCS17, DCS23 and DCS25 (Supplementary Table S1). Weight of unpeeled nuts ranged from 41.0 for DCS9 and DCS16 to 238.1 for DCC19 (Supplementary Table S1). DCC19, DCC24, DCC26 were observed to express higher values for weight of peeled nuts. The highest outturn (%) of 92.5% was observed for the DCC19 while the lowest outturn (%) value of 28.7% was exhibited by DCS 14 (Supplementary Table S1). There was no significant variability ((P > 0.05) among the Bunso progeny crosses for nut quality traits including brix, potential alcohol and firmness of nuts (Supplementary Table S1).

### Variation in yield components and nut quality traits of JX1 crosses

All yield related and nut quality traits varied significantly (P < 0.001; df = 141) among the JX1 crosses (Supplementary Table S2). Number of pods ranged from 1.3 for JX1/J1 × JX1/66, JX1/6 × JX1/27, and JX1/112 × JX1/23 to 54.6 for JX1/62 × JX1/54 (Supplementary Table S2). Pod weight of crosses JX1/62 × JX1/54 and JX1/73 × JX1/23 were about five-fold higher than pod weight of other crosses (e.g., JX1/10 × JX1/10, JX1/112 × JX1/23). The highest numbers of nuts per pod were observed for crosses JX1/21 × JX1/9, JX1/30 × JX1/45, JX1/34 × JX1/48, JX1/51 × JX1/20, JX1/6 × JX1/54, JX1/90 × JX1/51, JX1/J1 × JX1/51 and JX1/6 × JX1/113(P < 0.001) (Supplementary Table S2). Peeled nut weight ranged from 2.8 g for JX1/6 × JX1/27 to 98.7 g for JX1/73 × JX1/23 (Supplementary Table S2). Crosses JX1/80 × JX1/80, JX1/20 × JX1/34, JX1/10 × JX1/10, JX1/11 × JX1/23 and JX1/119 × JX1/119 had significantly higher outturn as compared to the rest of the crosses. JX1 crosses (JX1/24 × JX1/45, JX1/30 × JX1/7, JX1/31 × JX1/23, JX1/51 × JX1/25, JX1/51 × JX1/36, JX1/5 × JX1/9, JX1/51 × JX1/31, JX1/63 × JX1/23, JX1/J1 × JX1/6) were more than three-fold higher in brix as compared to other crosses (JX1/112 × JX1/23, JX1/2 × JX1/25, JX1/30 × JX1/51, JX1/6 × JX1/27, JX1/62 × JX1/54, JX1/51 × JX1/66 and JX1/23 × JX1/23) (Supplementary Table S2). Potential alcohol ranged from 1.73 for JX1/62 × JX1/51 to 14.6 for JX1/66 × JX1/23 and JX1/J1 × JX1/23 (Supplementary Table S2). The nuts of crosses JX1/30 × JX1/7, JX1/45 × JX1/45, JX1/62 × JX1/27, JX1/62 × JX1/7, JX1/63 × JX1/113 and JX1/J1 × JX1/33 expressed significantly (P < 0.001) higher firm nuts as compared to the rest of the crosses (Supplementary Table S2).

### Variation in yield and nut quality traits among the GX1 self-crosses

Significant differences (P < 0.001; df = 27) were observed among the GX1 self-crosses for yield related traits such as number of pods, pod weight, number of nuts/pod, unpeeled nut weight, and peeled nut weight. Variation among the crosses for outturn was not significant (P > 0.05) (Table 1). Number of pods ranged from 1.0 for GX1/25 × GX1/25 to 25.7 for GX1/30 × GX1/30 (Table 1). Pod weight for the GX1 self-crosses (GX1/16 × GX1/16, GX1/27 × GX1/27, GX1/30 × GX1/30, GX1/37 × GX1/37 and GX1/87 × GX1/87 were about twice that of some crosses (GX1/24 × GX1/24, GX1/29 × GX1/29, GX1/64 × GX1/64, GX1/86 × GX1/86) and more than ten-fold higher than pod weight of crosses GX1/2 × GX1/2, GX1/25 × GX1/25, GX1/3 × GX1/3 and GX1/72 × GX1/72 (Table 1). Number of nuts per pod was significantly higher for crosses GX1/16 × GX1/16, GX1/27 × GX1/27, GX1/30 × GX1/30, GX1/37 × GX1/37 and GX1/87 × GX1/87 as compared to the rest of the GX1 self-crosses (Table 1). Weight of unpeeled nuts and peeled nuts ranged from 5.7 for GX1/25 × GX1/25 to 139.5 for GX1/30 × GX1/30 and from 5.1 for GX1/25 × GX1/25 to 82.2 for GX1/37 × GX1/37, respectively (Table 1). Variability among the GX1 self-crosses for the nut quality traits; brix, potential alcohol and nut firmness was not significant (P > 0.05). Brix ranged from 3.4 for GX1/25 × GX1/25 to 17.0 for GX1/36 × GX1/36 while nut firmness ranged from 5.2 for GX1/25 × GX1/25 to 16.7 for GX1/24 × GX1/24 (Table 1).

### Variation in yield and nut quality traits among MX2 self-crosses

Differences among MX2 crosses for number of pods and pod weight were significant (P < 0.001; df = 26) (Table 2). Number of pods for crosses A1 × A1 was about five-fold higher than that of ATTA 3 × ATTA 3 and two-fold higher than those of crosses A10 × A10, A22 × A22, JB 10 × JB 10, JB 3 × JB 3, JB 40 × JB 40, P2-1B × P2-1B and W25 × W25 (Table 2). Pod weight ranged from 59.3 for ATTA 3 × ATTA 3 to 297.0 for A1 × A1 (Table 2). ATTA 3 × ATTA 3, A10 × A10, A8 × A8, and JB 9 × JB 9 were the topmost crosses with the highest number of nuts/pod of 7.0 (Table 2). All the crosses recorded above 50% outturn. However, outturn was significantly (P < 0.001) higher for A10 × A10, Club × Club, JB 15 × JB 15, JB 26 × JB 26, JB 3 × JB 3, JB 36 × JB 36, JB 9 × JB 9 and JB 34 × JB 34 (Table 2). Differences among the MX2 crosses for nut quality traits; brix, potential alcohol and firmness of nuts were also statistically significant (P < 0.001). For instance, brix of crosses ATTA 1 × ATTA 1, ATTA 3 × ATTA 3, Club × Club, JB 1 × JB 1, JB 10 × JB 10, JB 19 × JB 19, JB 26 × JB 26, P2-1B × P2-1B was about three-fold that of crosses JB 32 × JB 32, JB 35 × JB 35 and JB 34 × JB 34 (Table 2). Potential alcohol ranged from 4.5 for JB 3 × JB 3 to 10.5 for ATTA 1 × ATTA 1 (Table 2). JB 19 × JB 19, P2-1B × P2-1B, P2-1c × P2-1c were the top crosses with significantly (P < 0.001) higher values for nut firmness.

### Cluster and structuration of BUNSO progeny crosses based on pod set, yield and nut quality traits

**Figure 3:**
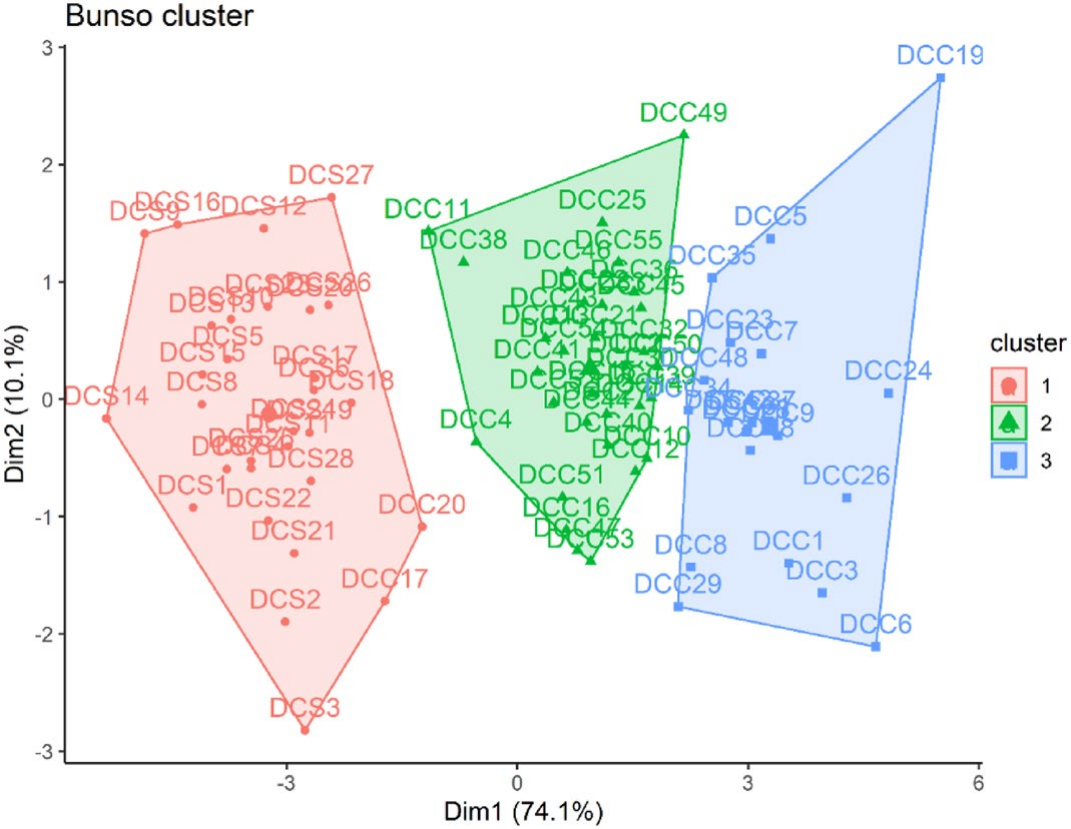
Cluster of double hybrid crosses of some Bunso progeny based on pod set and pod and nut traits.

Cluster analysis of double hybrid crosses of the Bunso progeny using pod set, pseudo-pod set and pod and nut traits grouped the crosses into 3 clusters (Fig. 3). Cluster 1 was composed of 30 individual crosses and cluster 2 had 35 individual crosses while Cluster 3 was made up of 18 crosses. Cluster 1 is characterized by crosses with significantly (P < 0.001) lower category means than overall mean of crosses for number of nuts/ pod, nut width, outturn, weight of unpeeled nuts, weight of peeled nuts, number of pods, pod weight, nut length and pod set (Supplementary Table S3). The category mean for weight of peeled nuts was two-fold lower than the overall mean of crosses for this trait (Supplementary Table S3). The category mean of cluster 2 for nut length, pod set, nut width, outturn and pod width were significantly (P < 0.001) higher than the overall mean of crosses for these traits (Supplementary Table S3). The standard deviation in category for cluster 2 ranged from 0.83 for nut length to 8.49 for pod set (Supplementary Table S3). Crosses grouped under cluster 3 expressed significantly higher category means for all traits than the overall mean of crosses (Supplementary Table S3) and the category standard deviation ranged from 0.86 for nut width to 34.30 for pod weight.

Structure analysis of the Bunso progeny crosses showed a clear separation of the double hybrid crosses and the double hybrid self-crosses. The double hybrid crosses were more distributed in the positive quadrant of the biplot while the double hybrid self-crosses were more distributed in the negative quadrants of the biplot (Supplementary Fig. S2). Dimension 1 which accounts for more than 74.1% of the total variability of the crosses was more associated with the double hybrid crosses (Supplementary Fig. S2). Dimension 2 which accounts for 10.1% of the total variability was more associated with double hybrid self-crosses (Supplementary Fig. S2).

### Cluster and structuration of JX1 crosses based on pod set, yield and nut quality traits

**Figure 4:**
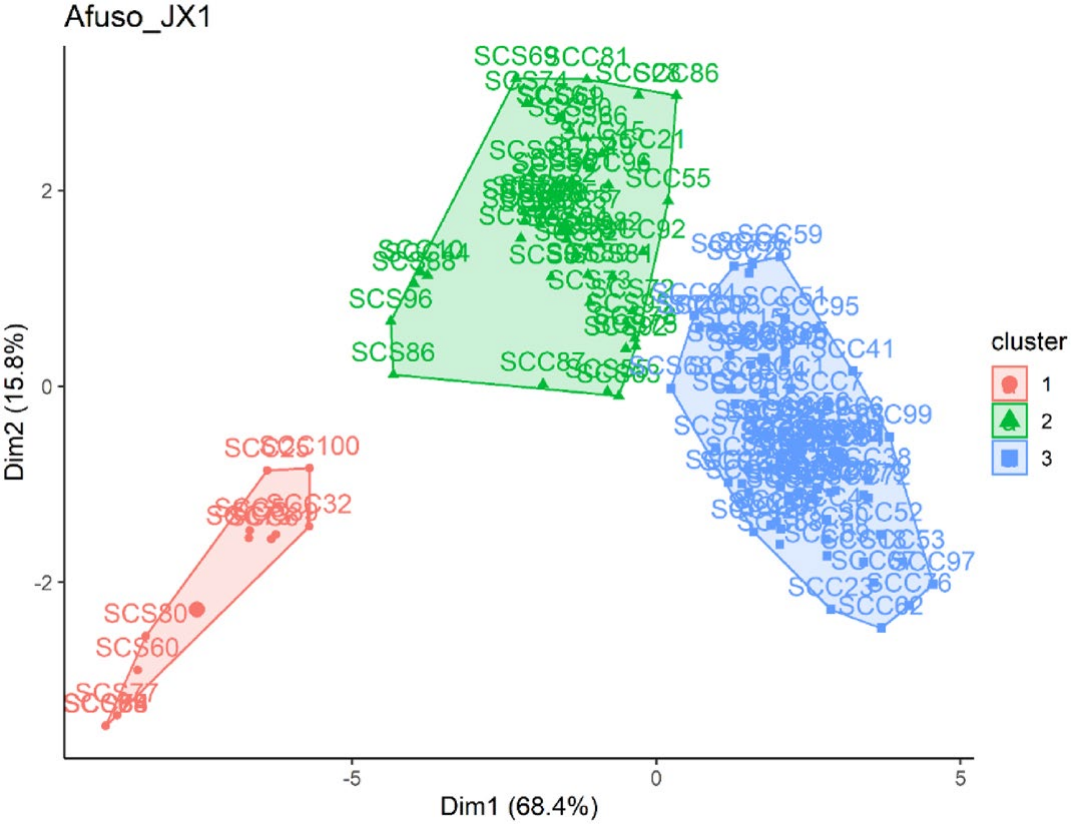
Cluster of single crosses of some JX1 genotypes based on pod set, yield components and nut quality traits.

The pod set, pseudo pod set and pod and nut traits of the JX1 crosses grouped the single cross and single self-crosses into 3 clusters (Fig. 4). Crosses in cluster 1 are characterized by traits that had category means significantly (P < 0.001) lower than the overall mean of the crosses (Supplementary Table S4). The category standard deviation for cluster 1 ranged from 1.04 for nut width to 11.69 for outturn while the standard deviation for all the crosses ranged from 2.10 for nut width (NW) to 80.32 for pod weight (PW) (Supplementary Table S4).

Structuration of JX1 crosses did not separate the single hybrid crosses and single hybrid self-crosses of JX1. The three groups of crosses were spatially distributed in all the four quadrants of the biplot. Single hybrid crosses of JX1 (SCC-JX1) were more associated with dimension 1 which contributed 68.4% of total variation. Also, the SCC-JX1 were more distributed on the positive quadrant of the biplot as compared to SCS-JX1 (Supplementary Fig. S3). The category means for crosses in cluster 2 for traits pseudo pod set, outturn, pod length, firmness of nuts was significantly (P < 0.001) higher than the overall mean of crosses for these traits. However, for traits such as nut length (NL), nut width (NW), number of nuts/pod (NN), weight of unpeeled nuts (WUN), weight of peeled nuts (WPN), pod weight (PW), number of pods (NP) and pod set (PS%), the category mean of crosses in cluster 2 was significantly (P < 0.001) lower than the overall mean of crosses (Supplementary Table S4). The category means of crosses in cluster 3 were significantly (P < 0.001) higher than the overall mean of crosses for the traits except for pseudo-pod set where the category mean was significantly (P < 0.001) lower than the overall mean of crosses (Supplementary Table S4).

### Cluster of GX1 single self-crosses based on pod set, yield components and nut quality traits

**Figure 5:**
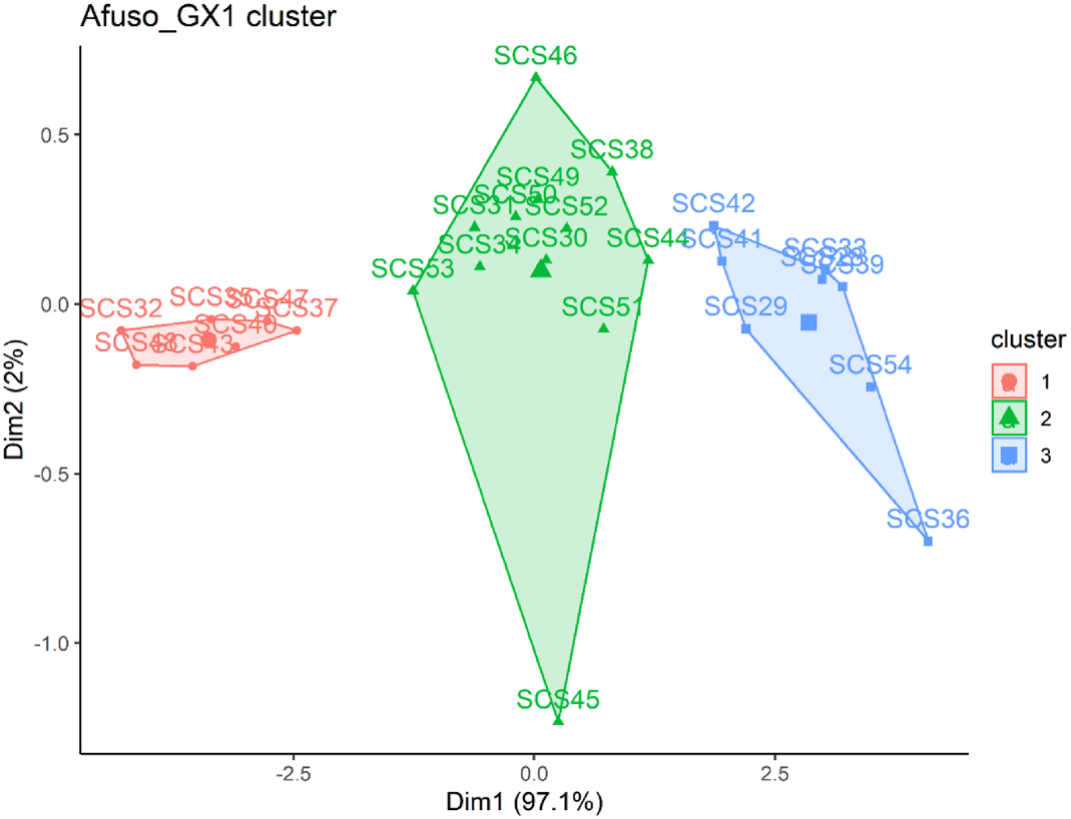
Cluster of GX1 single self crosses based on pod set, yield components and nut quality traits.

Pod set, pod and nut yield components and nut quality traits grouped the self-crosses of GX1 genotypes into three clusters (Fig. 5). Clusters 1, 2 and 3 encompassed of 7, 52 and 10 individual crosses, respectively. Cluster 1 was characterized by self-crosses that had significantly (P < 0.001) lower category mean for pod weight, number of pods, weight of unpeeled nuts, number of nuts per pod, pod set, weight of peeled nuts (Supplementary Table S5).

The self-crosses of GX1 genotypes grouped under cluster 3 exhibited category means that were significantly (P < 0.001) higher than the overall mean for weight of unpeeled nuts, number of nuts/pod, number of pod, pod weight, pod set and weight of peeled nut weight (Supplementary Table S5).

### Cluster of MX2 crosses based on pod set, yield components and nut quality traits

The self-crosses of MX2 were grouped into 5 clusters based on pod set, yield components and nut quality traits (Supplementary Fig. S4). Clusters 1, 2, 3, 4 and 5 had sizes of 2, 14, 18, 24, and 30 individual crosses, respectively. Cluster 1 was defined by self-crosses with significantly (P < 0.001) higher category mean than overall mean for nut length and nut width (Supplementary Table S6). Self-crosses in cluster 2 were characterized by significantly (P < 0.001) lower category mean for unpeeled nut weight, peeled nut weight, outturn and number of nuts/pod as compared to the overall mean of crosses for these traits (Supplementary Table S6).

Self-crosses in cluster 3 exhibited significantly (P < 0.001) higher category mean than overall mean of crosses for brix and pod width (Supplementary Table S6). Cluster 4 is defined by crosses with significantly (P < 0.001) higher category mean for number of pod, pod set and pod weight than the overall mean of crosses for these traits. However, the category mean of potential alcohol for crosses in cluster 4 was significantly (P < 0.001) lower than the overall mean indicating the crosses performed below average for this trait. The self-crosses of MX2 under cluster 5 exhibited significantly (P < 0.001) higher category mean than overall mean for number of nuts/pod, weight of unpeeled nuts, weight of peeled nuts, outturn and potential alcohol. The category mean for brix was however significantly (P < 0.001) lower than the overall mean (Supplementary Table S6).

### Correlation among pod set, yield and nut quality traits of BUNSO progeny crosses

Highly significant (P < 0.001) and positive correlation was observed between number of pods and pod set, pod weight and pod set, number of nuts per pod and number of pods, number of nuts per pod and pod weight, outturn and pod set, outturn and number of pods, outturn and pod weight, outturn and weight of unpeeled nuts, outturn and weight of peeled nuts (Supplementary Fig. S5).

### Correlation among pod set, yield and nut quality traits of JX1 crosses

Correlation was significant (P < 0.001) and positive for the relationship between number of pods and pod set, pod weight and pod set, number of nuts per pod and pod set, number of nuts per pod and number of pods, number of nuts per pod and pod weight, nut length and pod width, nut width and nut length, weight of unpeeled nuts and pod set, weight of unpeeled nuts and number of nuts per pod, weight of peeled nuts and pod set, weight of peeled nuts and number of pods, weight of peeled nuts and pod weight, weight of peeled nuts and number of nuts per pod, outturn and pod weight (Supplementary Fig. S6).

### Correlation among pod set, yield and nut quality traits of GX1 crosses

Significant (P < 0.001) and strong positive correlation was observed between number of pods and pod set, pod weight and pod set, pod weight and number of pods, number of nuts per pod and pod set, number of nuts per pod and number of pods, number of nuts per pod and pod weight, nut weight and nut length, weight of unpeeled nuts and number of pods, weight of unpeeled nuts and pod weight, weight of unpeeled nuts and number of nuts per pod, weight of peeled nuts and pod set, weight of peeled nuts and number of pods, weight of peeled nuts and number of pods, weight of peeled nuts and pod weight, weight of peeled nuts and number of nuts per pod, outturn and pod weight (Supplementary Fig. S7).

### Correlation among pod set, yield and nut quality traits of MX2 crosses

Highly significant (P < 0.001) and positive correlation was observed between number of pods and pod set, pod weight and pod set, pod weight and number of pods, nut weight and nut length, weight of unpeeled nuts and number of pods, weight of peeled nuts and number of nuts per pod, weight of peeled nuts and number of nuts per pod, weight of peeled nuts and weight of unpeeled nuts, outturn and number of nuts per pod, outturn and weight of unpeeled nuts, outturn and weight of peeled nuts (Supplementary Fig. S8).

### Heterosis for pod set, outturn and brix among the BUNSO progeny crosses

Mid parent, better parent and economic heterosis were prevalent for pod set, outturn and brix for most of the Bunso progeny crosses (Supplementary Table S7). Mid-parent heterosis for pod set (%) was positive for the double hybrid crosses and ranged from 26.02 for GX1/46 × GX1/53 × GX1/46 × GX1/16 to 242.49 for Club × JB 32 × JX1/5 × JX1/9. The top five crosses with higher positive mid parent heterosis for pod set included B1/11 × B1/71 × B1/157 × B1/149, B1/11 × B1/71 × B1/151 × B1/180, B1/212 × B1/236 × JX1/24 × JX1/22, B2/177 × B2/156 × B1/151 × B1/147and B1/211 × B1/200 × B1/157 × B1/149 (Supplementary Table S7). Equally, positive mid-parent heterosis was expressed by Bunso crosses for outturn. Percentage mid-parent heterosis for outturn for crosses B1/11 × B1/71 × B1/157 × B1/149, B1/11 × B1/71 × GX1/46 × GX1/53, B1/151 × B1/149 × B1/11 × B1/71, B1/212 × B1/210 × GX1/46 × GX1/53 is more than two-fold the positive mid-parent heterosis of B2/177 × B2/156 × JX1/9 × JX1/11, GX1/46 × GX1/16 × GX1/46 × GX1/53, GX1/46 × GX1/16 × JX1/9 × GX1/16, GX1/46 × GX1/53 × JX1/17 × JX1/5, JX1/23 × JX1/53 × GX1/46 × GX1/53, JX1/9 × JX1/11 × JX1/24 × JX1/22 and JX1/9 × JX1/11 × JX1/7 × JX1/5 (Supplementary Table S7). Unlike pod set and outturn, 45.28% and 54.71% of the BUNSO progeny crosses had negative and positive mid-parent heterosis for brix, respectively. The top five crosses with higher positive mid-parent heterosis for brix include JX1/9 × JX1/11 × JX1/17 × JX1/5, GX1/46 × GX1/16 × GX1/46 × GX1/53, B1/151 × B1/147 × GX1/46 × GX1/53, JX1/5 × JX1/9 × GX1/46 × GX1/16 and JX1/5 × JX1/9 × JX1/9 × JX1/11 (Supplementary Table S7).

Positive better parent heterosis was observed for pod set and outturn (Supplementary Table S7). B1/11 × B1/71 × B1/157 × B1/149, Club × JB32 × JX1/5 × JX1/9, B1/11 × B1/71 × B1/151 × B1/180, B1/211 × B1/200 × B1/157 × B1/149, B1/151 × B1/149 × B1/11 × B1/71 were the top five double hybrid crosses that exhibited higher better parent heterosis for pod set. Club × JB32 × JX1/5 × JX1/9, B11 × B1/71 × GX1/46 × GX1/16, B1/11 × B1/71 × B1/157 × B1/149, B1/11 × B1/71 × B1/151 × B1/180, B1/151 × B1/149 × B1/11 × B1/71, B1/208 × B1/180 × JX1/24 × JX1/22, B1/212 × B1/210 × GX1/46 × GX1/53 expressed higher better parent heterosis for percentage outturn. In relation to brix, 32.07% of the BUNSO progeny crosses had negative better parent heterosis. 67.92% of the crosses however were observed to have positive better parent heterosis for brix. Higher positive better parent heterosis values were observed for JX1/24 × JX1/22 × JX1/7 × JX1/53, JX1/7 × JX1/53 × JX1/7 × JX1/5, B1/151 × B1/149 × B1/11 × B1/71, B1/296 × B1/177 × GX1/46 × GX1/53, Club × JB 32 × JX1/5 × JX1/9, GX1/46 × GX1/16 × GX1/46 × GX1/53 and JX1/9 × JX1/11 × JX1/7 × JX1/53 (Supplementary Table S7).

In relation to standard variety 1, GX1/46 × GX1/16, an economic heterosis was prevalent for pod set (%), outturn (%) and brix (Supplementary Table S7). BUNSO progeny crosses that showed higher economic heterosis for pod set with regards to the standard variety 1 include B1/11 × B1/71 × B1/157 × B1/149, B1/151 × B1/147 × GX1/46 × GX1/53, Club × JB 32 × JX1/5 × JX1/9, GX1/46 × GX1/16 × JX1/9 × JX1/11, GX1/46 × GX1/33 × JX1/24 × JX1/22, GX1/46 × GX1/53 × B2/296 × B1/177, GX1/46 × GX1/53 × JX1/17 × JX1/5, and JX1/9 × JX1/11 × GX1/46 × GX1/53 (Supplementary Table S7). Crosses that expressed higher economic heterosis for outturn were B1/11 × B1/71 × B1/157 × B1/149, B1/11 × B1/71 × GX1/46 × GX1/16, B1/11 × B1/71 × GX1/46 × GX1/16, B/11 × B1/71 × GX1/46 × GX1/53, B1/120 × B1/193 × GX1/46 × GX1/53, B1/151 × B1/147 × GX1/46 × GX1/53, Club × JB32 × JX1/5 × JX1/9 and GX1/46 × GX1/16 × JX1/9 × JX1/11 (Supplementary Table S7).

Most of the crosses expressed negative economic heterosis for brix indicating that the crosses performed lower than standard variety 1 for brix content. However, few crosses including B1/11 × B1//71 × B1/151 × B1/180, B1/11 × B1/71 × B1/157 × B1/149, B1/11 × B1/71 × B1/296 × B1/177, B1/11 × B1/71 × B2/177 × B2/156, JX1/14 × JX1/32 × JX1/9 × JX1/11 had positive economic heterosis for brix in relation to standard variety 1. JX1/14 × JX1/32 × JX1/9 × JX1/11 was distinct for its higher economic heterosis for brix (Supplementary Table S7). Considering standard variety 2 (JX1/5 × JX1/9), there was also a prevalence of economic heterosis for pod set, outturn and brix (Supplementary Table S7). Economic heterosis for pod set was higher for B1/11 × B1/71 × B1/157 × B1/149, Club × JB 32 × JX1/5 × JX1/9, GX1/46 × GX1/16 × JX1/5 × JX1/9, GX1/46 × GX1/33 × JX1/24 × JX1/22, GX1/46 × GX1/53 × B2/296 × B1/177, GX1/46 × GX1/53 × JX1/17 × JX1/5 and JX1/9 × JX1/11 × GX1/46 × GX1/53 (Supplementary Table S7).

The top five Bunso double hybrid crosses that expressed higher economic heterosis for outturn in relation to standard variety 2 included Club × JB 32 × JX1/5 × JX1/9, B1/11 × B1/71 × GX1/46 × GX1/16, B1/11 × B1/71 × GX1/46 × GX1/53, B1/11 × B1/71 × B1/157 × B1/149 and GX1/46 × GX1/16 × JX1/9 × JX1/11 (Supplementary Table S7). Unlike standard variety 1, economic heterosis for brix in relation to standard variety 2 was positive for all the crosses. Crosses that expressed higher prevalence of positive heterosis for brix were B1/151 × B1/149 × B1/11 × B1/71, B1/211 × B1/200 × B1/157 × B1/149, Club × JB 32 × JX1/5 × JX1/9, GX1/46 × GX1/16 × JX1/9 × GX1/16, JX1/24 × JX1/22 × B1/151 × B1/147, JX1/9 × JX1/11 × JX1/7 × JX1/5 and JX1/9 × JX1/11 × JX1/7 × JX1/53 (Supplementary Table S7).

### Heterosis for pod set, outturn and brix among the JX1 single cross hybrids

There was prevalence of mid-parent, better parent and economic heterosis for pod set, outturn and brix for single cross hybrids of JX1 (Supplementary Table S8). Mid-parent for pod set was high for JX1/118 × JX1/23, JX1/119 × JX1/23, JX1/45 × JX1/6, JX1/51 × JX1/20, JX1/51 × JX1/36, JX1/63 × JX1/23, JX1/66 × JX1/23, JX1/73 × JX1/23, JX1/73 × JX1/6 and JX1/90 × JX1/51 (Supplementary Table S8).

High mid-parent heterosis for outturn was expressed by crosses JX1/117 × JX1/73, JX1/20 × JX1/23, JX1/21 × JX1/9, JX1/27 × JX1/25, JX1/31 × JX1/23, JX1/34 × JX1/23, JX1/51 × JX1/42, JX1/6 × JX1/113, JX1/6 × JX1/54, JX1/66 × JX1/23, JX1/73 × JX1/23, JX1/74 × JX1/108, JX1/87 × JX1/6, JX1/J1 × JX1/90 and JX1/J1 × JX1/6 (Supplementary Table S8). High positive mid-parent heterosis for brix was observed for JX1/20 × JX1/23, JX1/34 × JX1/48, JX1/51 × JX1/20, JX1/51 × JX1/34, JX1/66 × JX1/23, JX1/90 × JX1/51, JX1/J1 × JX1/90 and JX1/J1 × JX1/20 (Supplementary Table S8).

High better parent heterosis for pod set was expressed by crosses JX1/119 × JX1/32, JX1/119 × JX1/50, JX1/24 × JX1/6, JX1/30 × JX1/45, JX1/45 × JX1/6, JX1/51 × JX1/36, JX1/62 × JX1/6, JX1/63 × JX1/23, JX1/66 × JX1/23 and JX1/73 × JX1/23. Better parent heterosis for outturn was negative for most of the JX1 single hybrid crosses. Crosses that exhibited high positive better parent heterosis for outturn include JX1/117 × JX1/73, JX1/20 × JX1/34, JX1/6 × JX1/113, and JX1/6 × JX1/54 (Supplementary Table S8).

Prevalence of economic heterosis for pod set, outturn and brix was shown by the JX1 single hybrid crosses (Supplementary Table S8). With regards to standard variety 1, crosses JX1/1 × JX1/67, JX1/2 × JX1/45, JX1/20 × JX1/118, JX1/20 × JX1/31 and JX1/21 × JX1/9 had high positive economic heterosis for pod set. Economic heterosis for outturn was negative for most of the crosses except JX1/8 × JX1/112 and JX1/11 × JX1/23 which had positive economic heterosis for outturn in relation to standard variety 1. Crosses JX1/112 × JX1/23, JX1/2 × JX1/25, JX1/23 × JX1/34, JX1/30 × JX1/51, JX1/31 × JX1/50 and JX1/6 × JX1/27 were the top six crosses that expressed high positive economic heterosis for brix.

In relation to standard variety 2, economic heterosis was observed for pod set, outturn and brix (Supplementary Table S8). Crosses JX1/30 × JX1/51, JX1/51 × JX1/20, JX1/62 × JX1/54, JX1/63 × JX1/23, JX1/73 × JX1/23, JX1/73 × JX1/6 and JX1/51 × JX1/23 expressed high positive economic heterosis for pod set. Majority of the crosses expressed negative heterosis for outturn in relation to standard variety 2 with exception of JX1/1 × JX1/112, JX1/1 × JX1/67, JX1/11 × JX1/23, JX1/20 × JX1/34, JX1/35 × JX1/23, JX1/63 × JX1/4, and JX1/J1 × JX1/90 (Supplementary Table S8).

High positive economic heterosis for brix was expressed by JX1/112 × JX1/23, JX1/2 × JX1/25, JX1/23 × JX1/34, JX1/30 × JX1/51, JX1/31 × JX1/50, JX1/51 × JX1/25 and JX1/6 × JX1/27 in relation to standard variety 2 (Supplementary Table S8).

## Discussion

The results showed significant variation among Bunso progeny, JX1, GX1 and MX2 crosses for sexual compatibility suggesting there is a genetic variability within the gene banks to warrant selection and recovery of good performing lines for sexual compatibility. It is important to understand the amount of variation within a population in order to make a more informed selection decision^57,58^. This allows for planning crosses between compatible genotypes^59,60^. Nyadanu et al*.*^34^, Van Eijnatten^61^ and Jacob and Okoloko^41^ and Odutayo et al*.*^62^ also reported variations in sexual compatibility of kola cultivars. Efficient pollination and fertilisation depend most importantly on the presence of pollen from a compatible cultivar^45,63^. The variation observed within the kola germplasm collections at the Cocoa Research Institute of Ghana for self- and cross-compatibility provides opportunity to select self-compatible cultivars and cross-compatible partners for development of improved varieties. For instance, double hybrid crosses of the Bunso progeny such as Club × JB32 × JX1/5 × JX1/9, JX1/9 × JX1/11 × GX1/46 × GX1/53, B1/11 × B1/71 × GX1/46 × GX1/16, B1/120 × B1/193 × JX1/9 × JX1/11, B1/151 × B1/147 × GX1/46 × GX1/53, B1/296 × B1/177 × GX1/46 × GX1/53, GX1/46 × GX1/16 × JX1/17 × JX1/9, GX1/46 × GX1/16 × JX1/5 × JX1/9, GX1/46 × GX1/16 × JX1/9 × JX1/11, GX1/46 × GX1/33 × JX1/24 × JX1/22, GX1/46 × GX1/53 × B2/296 × B1/177, GX1/46 × GX1/53 × JX1/17 × JX1/5, JX1/23 × JX1/53 × GX1/46 × GX1/53, and JX1/7 × JX1/53 × JX1/7 × JX1/5 were classified as highly compatible crosses and would be good partners for development of compatible varieties. The top self-compatible crosses of the Bunso progeny were JX1/17 × JX1/9 × JX1/17 × JX1/9, JX1/23 × JX1/53 × JX1/23 × JX1/53, GX1/46 × GX1/53 × GX1/46 × GX1/53, JX1/9 × GX1/16 × JX1/9 × GX1/16 and JX1/9 × JX1/11 × JX1/9 × JX1/11. Involvement of these materials in kola breeding programmes would help in the improvement of the parental population and broaden the genetic base of elite kola germplasm for developing compatible varieties. These crosses would be important for developing self-compatible kola varieties.

Single hybrid crosses JX1/20 × JX1/118, JX1/21 × JX1/9, JX1/51 × JX1/23, JX1/51 × JX1/20, JX1/63 × JX1/23, JX1/73 × JX1/23, JX1/73 × JX1/6 and JX1/J1 × JX1/23 that expressed higher cross compatibility values would be good compatible partners for development of high yielding varieties. Self-crosses of JX1 (JX1/31 × JX131, JX1/108 × JX1/108), GX1 (GX1/27 × GX1/27, GX1/30 × GX1/30 and MX2 (A1 × A1, JB 1 × JB1 and JB37 × JB 37) that expressed moderately self-compatible class for self-compatibility could be great sources of genetic materials for developing self-compatible varieties. The selection of these crosses or genetic materials from different germplasm sources would help to capture genetic diversity available for the enhancement of elite lines and make this information accessible for breeding decisions^64–66^. Optimal cross-selection of these genotypes would increase genetic gain in breeding compatible varieties of kola^67,68^.

The genetic variability in pseudo-pod set was significant for the JX1 and MX2 crosses. Bunso progeny and GX1 crosses expressed non-significant variation for pseudo pod set. These differences among the crosses of the various germplasm collections could be due to differences in the genetic materials. Pseudo-compatibility is the fertilization by pollen with which they would normally be incompatible; incomplete incompatibility in which gametes which would normally be incompatible form a viable embryo or fruit set^40^. Pseudo-pod set is inversely related to pod set and results in low yield in kola. There is a need to consider pseudo pod set as one of the target traits to select against in breeding for sexual compatibility in kola. Crosses such as B1/11 × B1/71 × GX1/46 × GX1/16, Club × JB32 × JX1/5 × JX1/9, JX1/9 × JX1/11 × GX1/46 × GX1/53, JX1/20 × JX1/9, JX1/51 × JX1/20, JX1/5 × JX1/23 and JX1/73 × JX1/23, A1 × A1, JB1 × JB1 which were very highly compatible and expressed no pseudo-pod set would be useful genetic materials to select against pseudo-pod set. Variation in the level of pseudo-compatibility among genotypes in *Brassica oleracea* has been shown to depend on the genetic background in which the S genes operate^69^. Johnson^70^ reported pseudo-compatibility as one of the factors affecting the degree of self-incompatibility in inbred lines of Brussels sprouts.

The cross pollinations in general resulted to more pod set than the self-pollinations. Other authors reported that cross pollinations can enhance fertilization as shown by their higher fruit set reports^71,72^. Nevertheless, some genotypes were cross-incompatible and cannot fertilize each other. In some cases, this was expressed as low pod set. For example, among the single hybrids of JX1, 2% of the crosses made up of JX1/73 × JX1/36 and JX1/87 × JX1/118 were incompatible and 15 of the crosses (15% of total cross) exhibited very low compatibility. Selection against these crosses could help make progress in breeding for compatible varieties in kola. Griggs et al*.*^73^ and Cuevas and Polito^72^ reported similar findings in their work on olive.

The fairly good pod set of the double hybrid crosses as compared to the single hybrid crosses in this study could be due to an expression of genetic gains for pod set. The Bunso kola progenies were earlier selected for sexual compatibility or pod set in a hybrid breeding program^74^. However, the double hybrid self-pollinated crosses showed lower degree of compatibility reactions as observed in nearly all self -pollinations. This suggests that the phenomenon of self-incompatibility is expressed even in advanced generations of kola. This could have consequences of reduced yields in advanced generations of kola if recommended varieties are not inter-spaced with pollinizers and are solely established. The issue of self-incompatibility in advanced varieties of kola could be managed through inclusion of pollinizers in orchards to increase yield. In crops exhibiting SI, cultivars that serve as pollen donors (“pollenizers”) are usually interspersed throughout orchards since fruit set depends largely on cross pollinations. Pollenizers are commonly used in canola (*Brassica napus* L.), sunflower, strawberry (Fragaria x anannasa (Weston), European pear (*Pyrus communis*), sweet cherry, Japanese plum (*Prunus salicina* Lindl)^75–77^. The use of pollenizers is also recommended in olive (*Olea europaea* L.)^63^. Two genotypes A1 and B1 were identified to be great pollinizers of *Cola nitida* in Ghana^34^.

The falling or distribution of double and single crosses into higher compatibility scores than the double and single self-hybrid crosses further confirmed existence of self-incompatibility in kola. Self-incompatibility is a genetically controlled mechanism that prevents self-fertilization in about half of angiosperm species^37,78^. Sporophytic self-incompatibility has been reported in Asteraceae, Betulaceae, Convolvulaceae, sterculiaceae and malvaceae^40^. Kola is of the family malvaceae.

The significant variation observed among the kola crosses for pod yield related traits (number of pods, pod weight, pod length, pod width) and nut yield related traits (number of nuts/pod, nut length, nut width, peeled nut weight, unpeeled nut weight, outturn) present an opportunity to select kola hybrids and cultivars that combine high self or cross-compatibility with high yield and good nut attributes. DCC6, DCC9, DCC19, DCC26, DCC32, JX1/1 × JX1/67, JX1/2 × JX1/45, JX1/20 × JX1/118, JX1/21 × JX1/9, JX1/51 × JX1/23, JX1/73 × JX1/23 and JX1/73 × JX1/6 combined high pod and nut yield traits and high nut yield. Selecting these genetic materials would lead to purging of unfavourable alleles whilst retaining or even increasing frequency of favourable alleles for pod set, yield and nut quality. Variation in yield and its related traits in kola was also reported by Onomo et al*.*^79^, Akpertey et al*.*^80^,and Adebola et al*.*^81^.

Developing fruit crops with high qualities has been a goal in several breeding programs^82–85^. In this study, nuts of kola crosses were assessed for some important quality traits. The results confirmed that there were significant variations among the crosses for total soluble solids or brix for JX1 and MX2 kola gene banks. This suggests genetic diversity among the genotypes for brix and provides an opportunity to select crosses that are high in brix content. Brix was high for JX1/119 × JX1/32, JX1/24 × JX1/45, JX1/25 × JX1/99, JX1/30 × JX1/7, JX1/31 × JX1/23, JX1/51 × JX1/20, JX1/51 × JX1/25, JX1/51 × JX1/36, JX1/51 × JX1/42, JX1/51 × JX1/31, JX1/62 × JX1/6, JX1/63 × JX1/23, and JX1/73 × JX1/23. The top crosses with high brix for MX2 crosses were Club × Club, JB 10 × JB 10, JB 19 × JB 19, JB 26 × JB 26 and P2-1B × P2-1B. Brix is a measure of sugar or sweetness of fruits^86^. Considering the issue of astringency in kola, crosses that are high in brix could have an improved sensory attributes and good shelf-life during storage. The brix is very important because the higher the brix, the sweeter nut flavour and crop genotypes high in brix are preferred by consumers^87^. Astringency has been reported by Osei-Bonsu et al*.*^88^, Takrama et al*.*^35^ and Lowor et al*.*^89^ as a poor sensory attribute of kola consumption. Also, kola crosses with high brix content could store better during post-harvest storage. It is generally known that the higher the brix content of fruits, the better the shelf life during storage^90–92^.

Significant differences were observed among the crosses of JX1 and MX2 gene banks for potential alcohol. However, the variability in this trait for Bunso progeny and GX1 crosses was not statistically different. The difference in the significance of variation of this trait among different populations of kola could be explained by the differences in the genetic composition of genotypes and the environments where the accessions are established. Kola is used to develop various products including soft drinks and wine^17^. Varieties with higher potential alcohol content are therefore desirable. Potential alcohol does not only influence flavour and sensory perceptions of kola nuts^93^ but serve as one of the principal characters required in the development and improvement of kola genotypes for domestic and industrial uses. JX1 crosses (JX1/27 × JX1/25, JX1/66 × JX1/23, JX1/74 × JX1/108, JX1/8 × JX1/119, JX1/8 × JX1/23, JX1/89 × JX1/63, JX1/90 × JX1/51, JX1/J1 × JX1/59, JX1/J1 × JX1/23, JX1/J1 × JX1/66) and MX2 self-crosses (A10 × A10, A8 × A8, Atta1 × Atta 1, JB 9 × JB 9, and JB34 × JB 34) expressed high potential alcohol content. Though not significant, the following crosses of Bunso progeny ( DCS7, DCC1, DCS13, DCC33, DCC43, DCC51) and GX1 crosses (GX1/3 × GX1/3, GX1/30 × GX1/30, GX1/37 × GX1/37, GX1/60 × GX1/60, GX1/74 × GX1/74 and GX1/71 × GX1/71) had high potential alcohol content compared to the other crosses. These crosses would be important for developing industrial products like wine.

Fruit or nut firmness is an important component of texture and influences sensory perception of consumers. Consumers regard texture as a positive quality attribute donating freshness and storability of products and contributing to the enjoyment of eating^94,95^. Texture properties are very important in foods for harvesting, processing, packaging, storage and presentation to the consumer/customer. For example, hardness/firmness, one of the texture properties, is one of the most substantial parameters generally used to determine freshness of fruits and vegetables^94^. All the four populations of kola used in this study did not exhibit significant differences among nuts of crosses for nut firmness except JX1 crosses. The top six crosses of the JX1 with higher nut firmness were JX1/30 × JX1/7, JX1/45 × JX1/45, JX1/62 × JX1/27, JX1/62 × JX1/7, JX1/63 × JX1/113 and JX1/J1 × JX1/33. These materials are great resources to improve nut firmness attribute of kola hybrids.

Cluster analysis was carried out to group crosses having similar performance in relation to pod set, pseudo-pod set, yield components and nut quality traits. Quantitative analysis regrouped the crosses into 3 clusters for Bunso progeny, JX1 and GX1 gene banks and 5 clusters for the MX2 gene bank which facilitates the selection of diverse crosses or parents for the kola breeding programme. Based on the results, the diversity panel was categorized into three clusters with cluster 2 and 3 containing the best performing crosses for Bunso progeny, JX1 and GX1. Crosses in cluster 2 and 3 for Bunso progeny, JX1 and GX1 and cluster 4 and 5 of MX2 were more distributed at the positive side of the quadrant and were more associated with dimension 1. The dimension 1 contributed 74.1%, 68.4%, 97.1% and 27.4% of the total variability for Bunso progeny, JX1, GX1 and MX2 crosses respectively. Given the information on the contribution of the traits to variation on the PC1 and PC2 axes, the biplots identified the genetic materials with high compatibility, yield and nut quality. The higher category means than overall mean for traits of the crosses that constitute cluster 2 and 3 and 4 and 5 of MX2 indicates that they performed above average of all the crosses. Such crosses would be rewarding if selected and used in kola breeding programmes to develop compatible and high yielding varieties.

The significant strong correlations of pod set, yield traits such as number of pods, pod weight, number of nuts/pod, weight of unpeeled nuts, weight of peeled nuts and outturn for Bunso progeny, JX1 and GX1 crosses suggested that these economic traits could be improved simultaneously. These results further suggest possible deployment of indirect selection for nut yield using pod set, pod weight, nut width and nut length as surrogate traits in kola breeding programmes. This agrees the findings of Nyadanu et al*.*^34^ and Adebola et al*.*^81^ who found significant and positive correlation between pod set and number of pods, number of nuts /pod, and nut weight. The negative and significant correlations between pod set and pseudo pod set indicates that an increase in pseudo-pod set would result in a decrease in pod set. This necessitates the need to select against pseudo-pod set in kola breeding programmes. Correlation between the nut quality traits and the yield traits was weak and not significant for Bunso progeny crosses. Similarly, correlation between the nut quality traits and the yield traits was weak and not significant for the MX2 crosses except correlation between brix and nut length (r = 0.46, P < 0.05), brix and nut width (r = 0.38, P < 0.05), firmness of nuts and nut length (r = 0.47, P < 0.05). However, in the case of JX1 and GX1 crosses, significantly strong correlations were observed between the nut quality traits and the agronomic/yield traits. For instance, the correlation between brix and pod length and between brix and outturn was 0.81 and 0.84 respectively for JX1 accessions. Similarly, the correlation between brix and outturn and brix and nut width was 0.6 and 0.56 respectively for GX1 accessions. The association of yield traits and nut quality traits for these genetic resources of kola allows the selection of promising genotypes that combines high yield with nut quality traits^96,97^. Due to the high demand and the search for new hybrids that meet the requirements of the consumer market, breeding strategies consist of exploring important agronomic traits and improvements in organoleptic properties to favour both higher quality and production. The contrasting results for the kola germplasm sets could be explained by genetic differences of their genotypes and the environments in which they were grown. Brix is affected by a lot of factors including genetics and environmental factors^98–100^. Selection and involvement of kola genotypes identified in this study to have appreciable higher contents of brix in breeding programmes could help develop improved varieties with enhanced nut quality.

The high prevalence of positive estimates of MPH, BPH and ECH for pod set and outturn among the double hybrid crosses of Bunso progeny suggest that the crosses performed better than their parents and standard varieties for this trait and further indicate absence of bidirectional dominance deviation. The prevalence positive heterosis observed among the single hybrid crosses of JX1 for pod set, outturn and brix indicates that effective progress can be made in the development of compatible and high yield kola varieties with quality nuts. The expression of high and positive heterosis among the double hybrids of Bunso progeny for pod set and outturn and pod set, outturn and brix in the case of JX1 crosses could be dependent on the degree of fit and genetic diversity^58,101^ among the parental lines used which is worth testing to inform future decision making in the kola breeding pipeline. This could further be explained by the additive effects of several desired dominant alleles, or as ‘overdominance’ the combined effect of two different alleles at the same gene locus, or a combination of both^102–105^. Hence, heterosis helps a breeder to make more stringent selections. The parents of the double hybrid crosses were selected earlier for their high fruit set or compatibility potentials^74^. Hence linkats containing favourable gene reassortments, especially in linkage disequilibrium, could be preserved. This may account for the very large mid-parent heterosis and heterobeltiosis noted for the double hybrid crosses which was above that of single hybrid crosses. It would be interesting to check if further cross-breeding of the double hybrid crosses could maximize progressive heterosis responses resulting in even higher compatibility in third generation of hybrid crosses.

Lamkey and Edwards^106^ and Alam et al*.*^107^ suggested that positive heterosis is desired in the selection for yield and its components, whereas negative heterosis is desired for early cycling and short plant height. In our case however, a positive heterobeltiosis for compatibility, outturn and brix was desirable since it indicates that the crosses were more compatible, had higher nut outturn and brix than their parents.

## Conclusion

Significant and large variations for sexual compatibility, nut yield and nut quality attributes were observed for crosses of Bunso progeny, JX1, GX1 and MX2 field genebanks of kola in Ghana. The cross pollinations in general resulted to more than two-fold pod set than the self-pollinations confirming the need for pollinizers to increase fruit set in kola. Self-compatible and cross compatible partners within single hybrid and double hybrid crosses were identified. Strong and significant correlation was observed between sexual compatibility and number of pods, pod weight, number of nuts/pod and outturn for the crosses of all the four field gene banks of kola. Significant and positive correlation between yield and nut quality traits observed for JX1 and GX1 crosses provides opportunity to simultaneously develop kola varieties that combine high yield with good nut quality. Very large prevalence of mid-parent heterosis, heterobeltiosis and economic heterosis was observed for the double hybrid crosses of Bunso progeny and single hybrid crosses. Mid-parent heterosis, heterobeltiosis and economic heterosis observed for the double hybrid crosses was above that of single hybrid crosses. This indicates that recurrent selection of compatible kola varieties from advanced generations of kola could be rewarding. The top five crosses with best heterosis for sexual compatibility and an appreciable positive heterosis for outturn and brix were B1/11 × B1/71 × B1/157 × B1/149, B1/11 × B1/71 × B1/296 × B1/177, GX1/46 × GX1/33 × B1/212 × B1/236, JX1/90 × JX1/51 and JX1/51 × JX1/36. The top very highly compatible kola partners identified in this study included B1/11 × B1/71 × GX1/46 × GX1/16, Club x JB 32 × JX1/5 × JX1/9, GX1/46 × GX1/16 × JX1/5 × JX1/9, GX1/46 × GX1/16 × JX1/5 × JX1/9, GX1/46 × GX1/53 × JX1/17 × JX1/5, JX1/9 × JX1/11 × GX1/46 × GX1/53, JX1/21 × JX1/9, JX1/51 × JX1/23, JX1/51xJX1/20, JX1/63 × JX1/23, JX1/73 × JX1/6, and JX1/J1 x JX1/23. The top self-compatible crosses included JX1/17 × JX1/9 × JX1/17 × JX1/9, JX1/23 × JX1/53 × JX1/23 × JX1/53, GX1/46 × GX1/53 × GX1/46 × GX1/53, JX1/9 × GX1/16 × JX1/9 × GX1/16, JX1/9 × JX1/11 × JX1/9 × JX1/11 JX1/31 × JX131, JX1/108 × JX1/108, GX1/27 × GX1/27, GX1/30 × GX1/30, A1 × A1, JB 1 × JB1 and JB37 × JB 37. Involvement of these materials from different germplasm collections in the Ghanaian kola breeding programme could help to capture genetic diversity available in the improvement of the parental population and broaden the genetic base of elite kola germplasm for developing compatible varieties.

## Materials and methods

### Germplasm and experimental sites

The source of plant materials used for the study is the Cocoa Research Institute of Ghana (CRIG). The experiment was conducted on four different kola field gene banks held at CRIG namely, MX2, JX1, GX1 and Bunso progeny (advanced germplasm). All the materials were collected under the authority of CRIG in Ghana and all procedures were carried out in accordance with relevant guidelines for handling of plant genetic resources.

Assessment of self-compatibility and cross-compatibility of genotypes within the various gene banks was carried out from January 2019 to December 2020. MX2 kola field gene bank is located at CRIG, Tafo. CRIG is located at an altitude of 222 m above sea level with a minimum and maximum temperature of 21 to 37 °C, respectively. The weather conditions during 2019 and 2020 at CRIG are shown in Supplementary Fig. S9. JX1 and GX1 kola field gene banks are situated at Afosu. Afosu is a marginal area in terms of rainfall. Amount of rainfall received in the area is between 150 and 200 cm reaching its maximum during the two peak periods of May–June and September–October yearly with 25.2 °C and 27.9 °C as minimum and maximum temperatures, respectively. The Bekwai-Oda association is the predominant soil formation in the area. The weather conditions in 2019 and 2020 at Afosu is presented in Supplementary Fig. S10. Bunso progeny trial is located at Bunso. Bunso is located at altitude 145.00 m/475.72ft above sea level with minimum and maximum temperature of 23.2 °C and 34.9 °C, respectively. Supplementary Fig. S11 shows the weather conditions at Bunso during the experimental period in 2019 and 2020.

The physical and chemical properties of the soils at MX2 (Tafo); JX1 and GX1 (Afosu), and Bunso progeny trial (Bunso) are presented in Supplementary Table S9. Information on the year of establishment and the number of accessions of the four field gene banks of kola are presented in Supplementary Table S10.

### Assessment of sexual compatibility within the germplasm collections

From May 2019 to December 2020, crossing of some genotypes of MX2, JX1, GX1 and Bunso progeny trial was carried out. Partners used in the crossing depends on the availability of male and female flowers for the genotypes involved as flowering in the species is erratic across years. Twenty pollinations/crossings were targeted per each cross. The crossings/pollination was replicated thrice using three pollinators. Before pollination, female flowers about to open were covered or bagged with nylon nets and the male flowers were emasculated before pollen maturation. Freshly opened female flowers were pollinated with pollen grains from newly opened male flowers. Pollen grains from newly opened male flowers were collected with 15 cm thin sticks with sharp ends and smeared to each of the stigmatic lobes of freshly opened female flowers. Pollinated female flowers were re-bagged immediately with nylon nets for further 48 h. For each of the crosses carried out, pod sets, pseudo-pod sets, number of dropped flowers after pollination were recorded two weeks after pollination. Based on the records of pod sets, the crosses were categorized into incompatible, very low compatible, low compatible, moderate compatible, high compatible and very high compatible using a 0–5 scale—0 (no pod set), 1 (1–20% pod set), 2 (21–40% pod set), 3 (41–60% pod set), 4 (61–80% pod set) and 5 (81–100% pod set). The proportion of fruit set to number of flowers pollinated was expressed in percentage and noted as percentage pod set as shown in Eq. (1). The proportion of number of pseudo fruit set to the number of flowers pollinated was expressed in percentage and noted as pseudo pod set as shown in Eq. (2).

1 $$\mathrm{Pod \; set }\left(\mathrm{\%}\right)=\frac{Number \; of \; fruit \; set}{Number \; of \; flowers \; pollinated}\times 100,$$

2 $$\mathrm{Pseudo \; pod \; set }\left(\mathrm{\%}\right)=\frac{Number \; of \; Pseudo \; fruit \; set}{Number \; of \; flowers \; pollinated}\times 100.$$

### Measurement of yield components of pod and nuts

The pods were harvested 130 days after pollination to ensure all the nuts of the kola accessions are of same maturity and data were collected on yield and yield components of pods and nuts. Number of pods harvested per each cross was counted and recorded. Pod length and width were measured using tape measure (Royal Dockyard Tape Measure—KA037) and digital caliper (NEIKO Stainless Digital Caliper 01407A) respectively. The weight of the pods was measured using weighing balance. The pods were broken and the number of nuts per pod was counted and recorded. Nut length and width were measured using digital caliper. Weight of nuts with the peels and weight of the nuts after removing the peels were measured using the weighing balance. The proportion of the total peeled weight of the nut of each cross to the weight of fresh unpeeled nuts was estimated as the outturn as shown in the formula below.

3 $$\mathrm{Outturn }\left(\mathrm{\%}\right)=\frac{Weight \; of \; peeled \; nuts \left(g\right)}{Weight \; of \; unpeeled \; nuts \left(g\right)}\times 100.$$

### Determination of firmness, brix and potential alcohol content of the kola nuts

Firmness of the nuts from each cross within MX2, JX1, GX1 and Bunso progeny trial was determined using a handheld penetrometer (EFFEGI model ft327 (3-27Lbs, Italy). The nuts collected were stored in a nylon net and kept at room temperature for 24 h to ensure uniform temperature of nuts of the various crosses. A disc of about 2 cm in diameter was peeled of the skin of each nut using a stainless-steel vegetable peeler. A plunger with the tip size 5/16 was used. The plunger was forced into the nut through the peeled surface with the nuts against a stationary hard surface with a uniform speed of about 2 s. Depth of penetration was consistent to the inscribed line on the tip of the plunger. Readings were recorded in poundforce (lbf).

To measure the brix, the refractometer (HANNA instruments HI 968113, Romania) was calibrated using distilled water. The nuts of the crosses were crushed using mortar and pestle. The juice in the crushed nuts was then sieved through cheeth cloth into glass beaker. A disposable plastic pipette was then used to drop the juices into the window of the refractometer. Total soluble solids (TSS) was determined with a digital refractometer and was expressed in Brix^0^. Potential alcohol content of the juice from the nuts of the various crosses was also measured and recorded.

### Statistical analyses

Data collected were analysed using the R environment (V. 3.6.2)^108^. To test the effect of the crossing type on pod and pseudo-pod set, a generalised linear model with a binomial distribution error to the data organized as success and failure events was fitted. The prominence of compatibility classes within each specific cross type was compared by using a multiple proportion comparison test from the *prop.test ()* function of base R. An analysis of variance or a Kruskall-wallis test (where relevant) was used to assess the effect of the cross type on the pod and pseudo-pod yield as well as on the nut firmness, brix and potential alcohol content. When the analysis showed significance, mean comparisons were made according to the least significance difference test at P < 0.05 (LSD_0.05_).To understand the grouping patterns of different type of crosses based on pod and pseudo-pod traits, a hierarchical clustering on principal component analysis was computed using the FactorMineR package^109^, and the graphical outputs were visualized using the factoextra package^110^. The correlation strength and significance among different pod and pseudo-pod traits was assessed using function corr_plot () of the Metan package^111^.

Heterosis for sexual compatibility (pod set %), outturn and brix were computed using the overall mean of each cross over replications. The heterosis of single cross hybrids and double cross hybrids were computed after Fehr and (1987)^112^. Relative heterosis or mid parent heterosis was estimated as percent deviation of hybrid cross value from its mid-parental self-cross value. The formulae used for estimating the relative heterosis was as follows:

4 $$Relative \; heterosis \left(\%\right)\left(di\right)=\frac{F1-MP}{MP}\times 100,$$

where di is the Heterosis over mid parental value (relative heterosis), F1 is the mean hybrid cross performance for pod set, outturn and brix. MP is the mid parental value, i.e., the arithmetic mean of self-cross pod set, outturn and brix of two parents involved in the respective cross combination.

5 $$Mid \; parent =\frac{P1+P2}{2}.$$

Heterobeltiosis was calculated as the superiority of hybrid cross for pod set, outturn and brix from the better parent self-cross pod set as follows:

6 $$Heterobeltiosis \left(\%\right)(dii)=\frac{F1-BP}{BP}\times 100,$$

where dii is the Heterobeltiosis (heterosis over better parent). BP is the average performance of the better parent with respect to the cross combination for pod set, outturn and brix.

7 $$Economic \; heterosis \left(\%\right)=\frac{F1-Standard \; variety}{Standard \; variety}\times 100.$$

Two recommended varieties of kola were used for the estimation. Standard variety 1 = GX1/46 × GX1/16 and standard variety 2 = JX1/5 × JX1/9.

## Supplementary Information

Supplementary Information 1.

Supplementary Information 2.

Supplementary Information 3.

## Data Availability

The datasets generated and or analyzed during the current study are available and presented in the supplementary materials as Raw Data S1 Bunso progeny, Raw Data S2 JX1, Raw Data S3 GX1 and Raw Data S4 MX2.
