# Supplementary material for: Heterosis patterns and sources of self-compatibility, cross-compatibility and key nut traits within single and double hybrid crosses of kola [Cola nitida (Vent) Schott and Endl.]
Source: Sci Rep. 2023 May 17;13:8036. doi: 10.1038/s41598-023-30485-3 (PMC10192454; doi:10.1038/s41598-023-30485-3)
Supplement: Supplementary file 2 — Supplementary Information 2. [file 41598_2023_30485_MOESM2_ESM.docx]

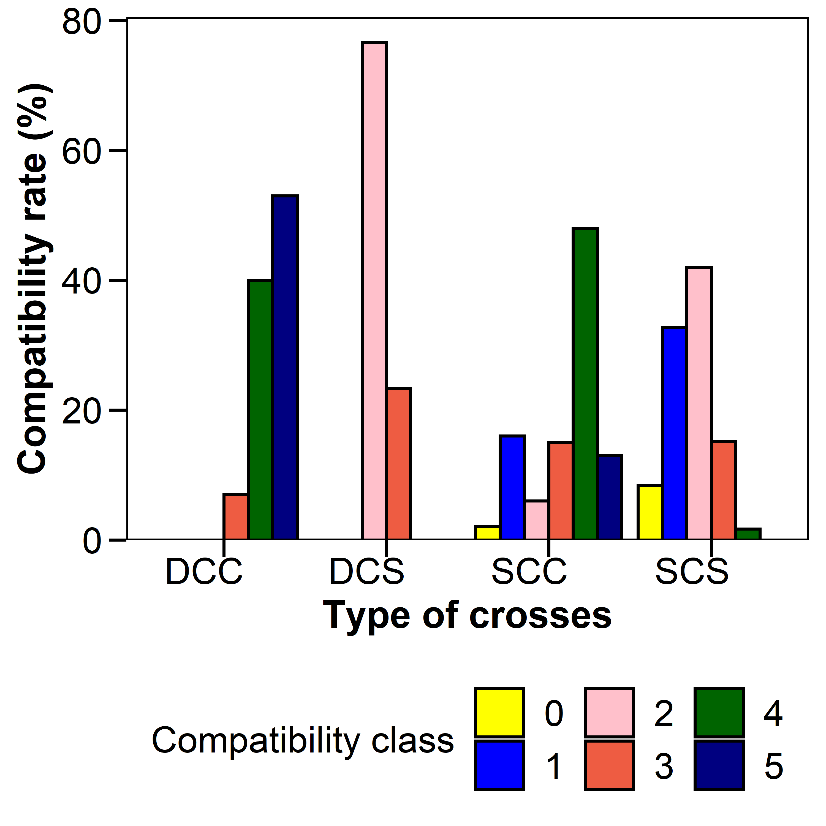


**Supplementary Figure S1.** Distribution of compatibility scores for percentage pod set among double hybrid crosses and single hybrid crosses. DCC = double hybrid crosses; SCC = single hybrid crosses; DCS = double hybrid self-crosses and SCS = single hybrid self-crosses


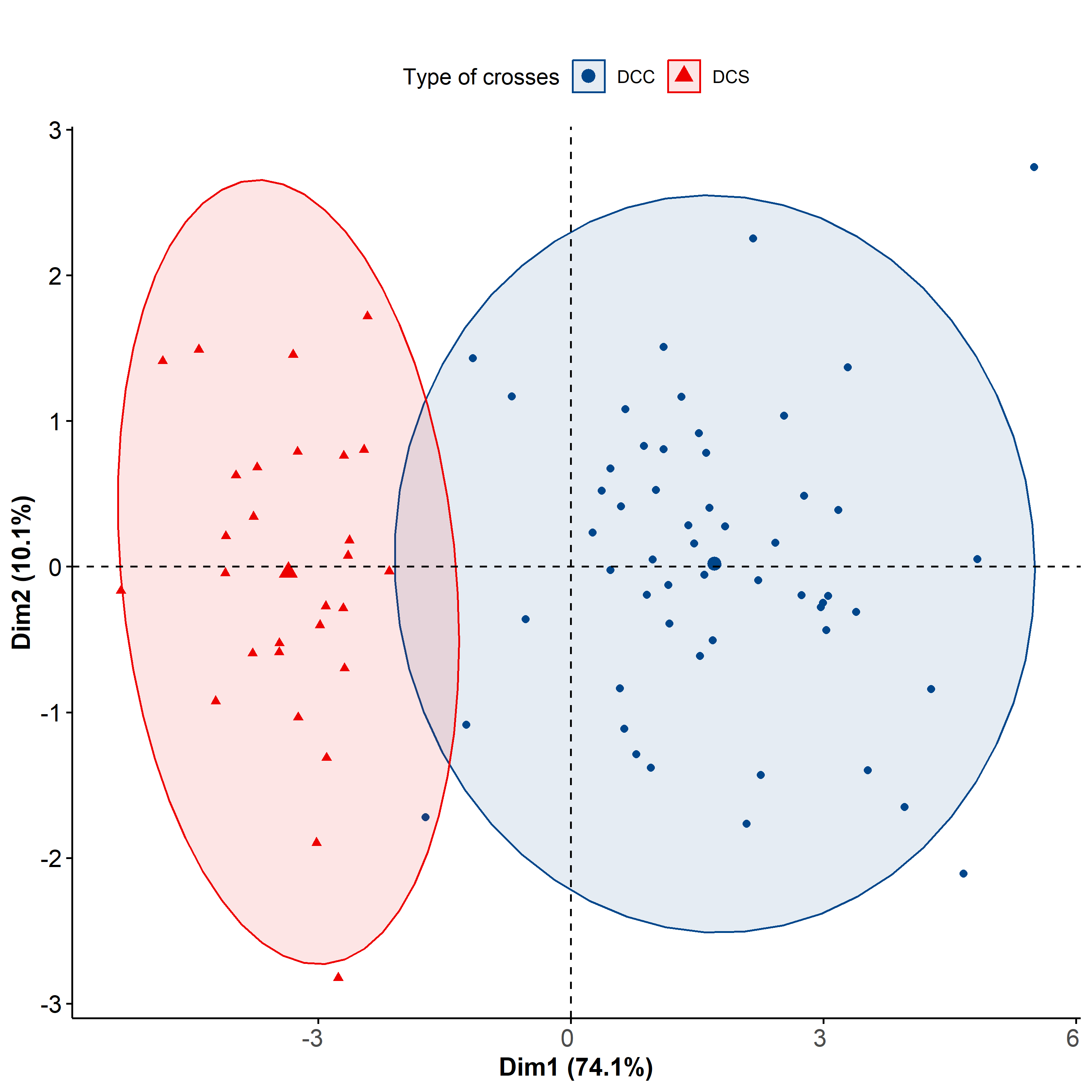


**Supplementary Figure S2**. Structuration of crosses of Bunso progeny based on pod set, yield and nut quality traits. DCC = Double hybrid crosses, DCS = Double hybrid self-crosses


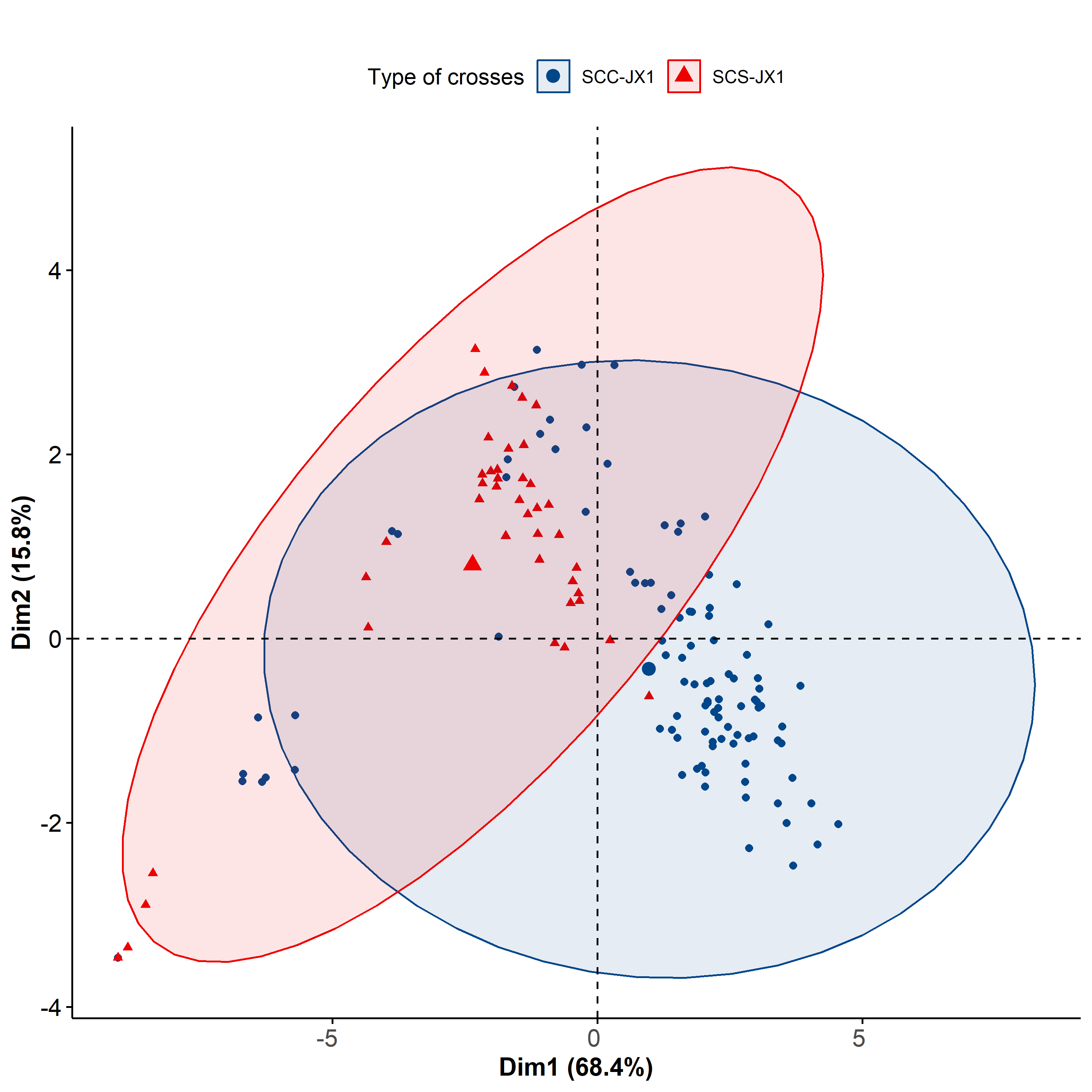


**Supplementary Figure S3**. Structuration of JX1 single crosses and single self-crosses based on pod set, yield components and nut quality traits.


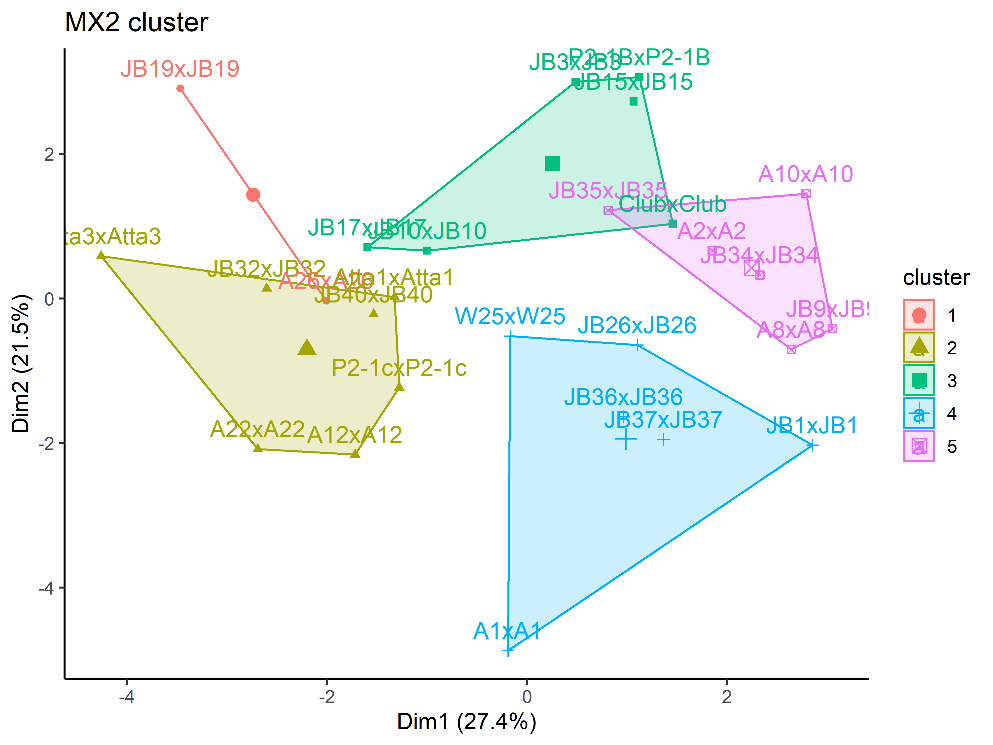


**Supplementary Figure S4.** Cluster of single-self crosses of some MX2 genotypes based on pod set, yield components and nut quality traits.


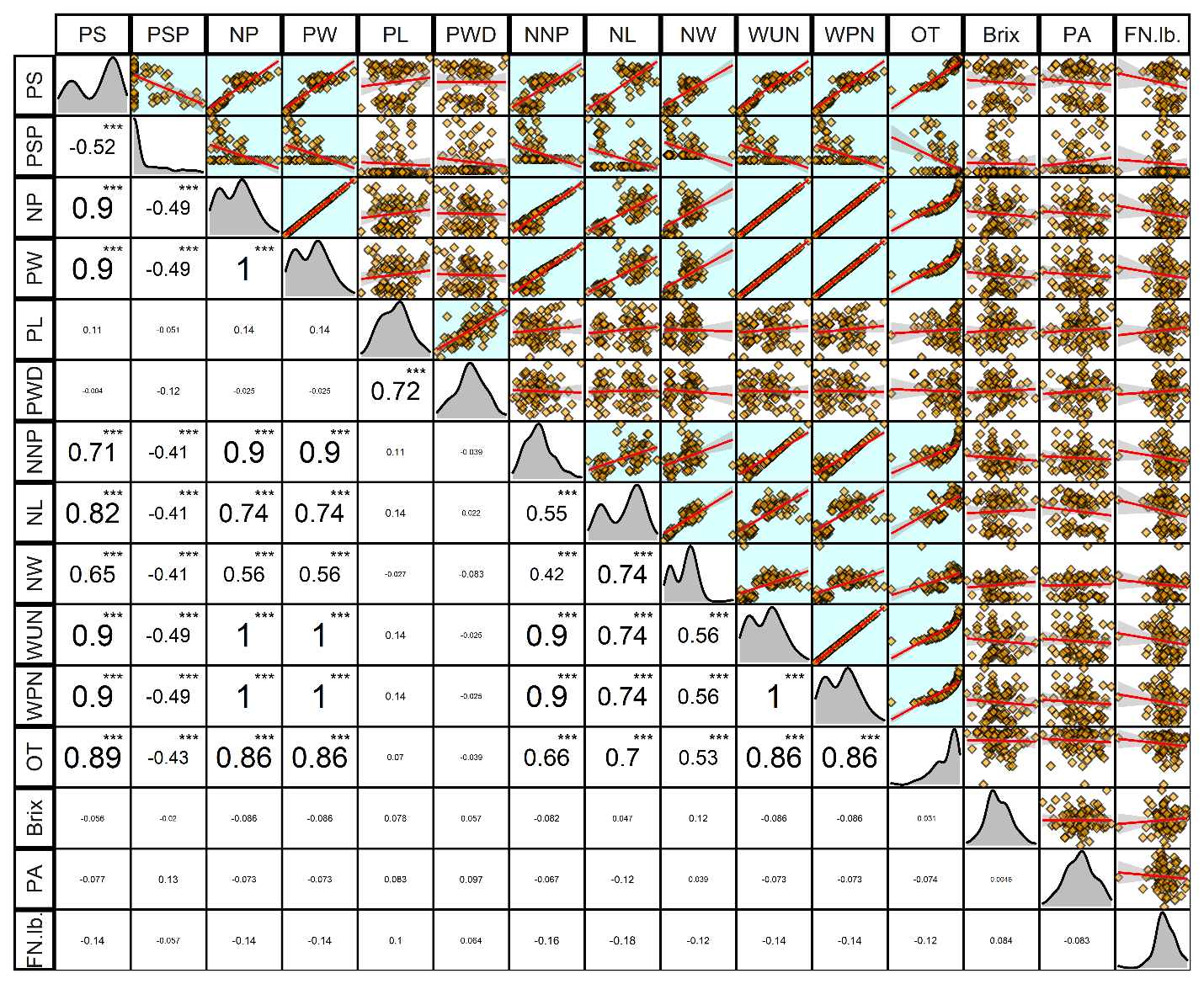


**Supplementary Figure S5.** Correlations among pod set, yield components and nut quality traits of BUNSO progeny double hybrid crosses. PS = Pod set (%), PSP =Pseudo pod set (%), NP = number of pods, PW = Pod weight (g), PL = Pod length (cm), PWD =Pod width (cm), NNP = Number of nuts per pod, NL = Nut length (cm), NW = Nut width (cm), WUN = Weight of unpeeled nuts (g), WPN = Weight of peeled nuts (g), OT = Outturn (%), PA= Potential alcohol, FN.Ib = Firmness of nuts (Ib).


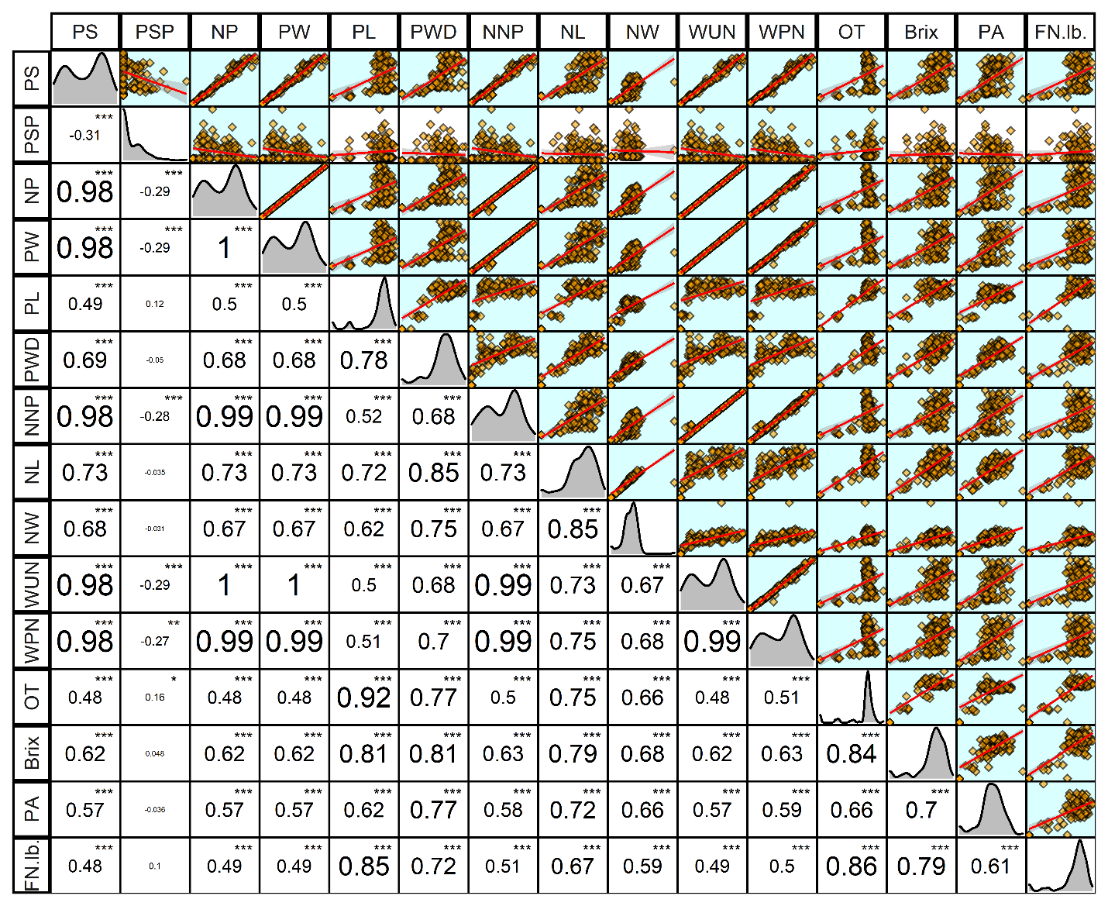


**Supplementary Figure S6**. Correlation among pod set, yield components and nut quality traits of JX1 crosses. PS = Pod set (%), PSP =Pseudo pod-set (%), NP = number of pods, PW = Pod weight (g), PL = Pod length (cm), PWD =Pod width (cm), NNP = Number of nuts per pod, NL = Nut length (cm), NW = Nut width (cm), WUN = Weight of unpeeled nuts (g), WPN = Weight of peeled nuts (g), OT = Outturn (%), PA= Potential alcohol, FN.Ib = Firmness of nuts (Ib)


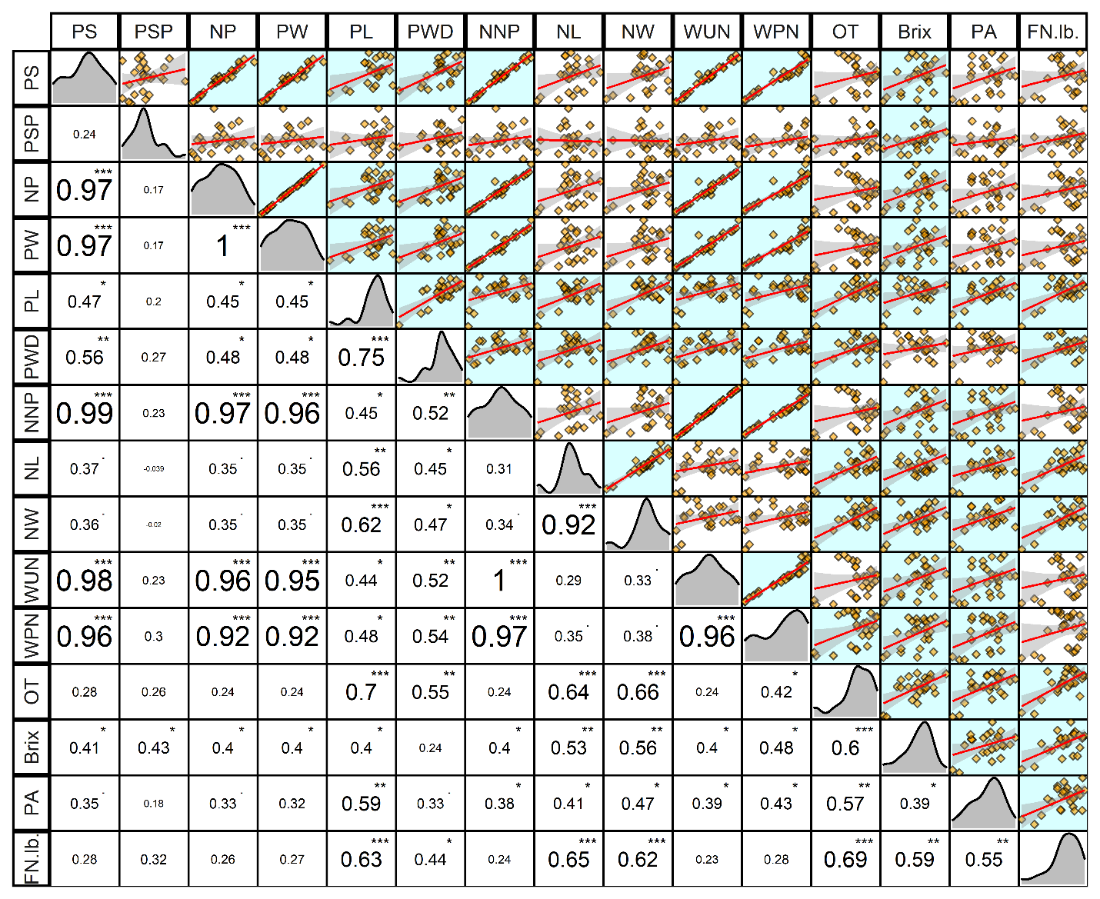


**Supplementary Figure S7.** Correlations among pod set, yield components and nut quality traits of GX1 self-crosses. PS = Pod set (%), PSP =Pseudo-pod set (%), NP = number of pods, PW = Pod weight (g), PL = Pod length (cm), PWD =Pod width (cm), NNP = Number of nuts per pod, NL = Nut length (cm), NW = Nut width (cm), WUN = Weight of unpeeled nuts (g), WPN = Weight of peeled nuts (g), OT = Outturn (%), PA= Potential alcohol, FN.Ib = Firmness of nuts (Ib)


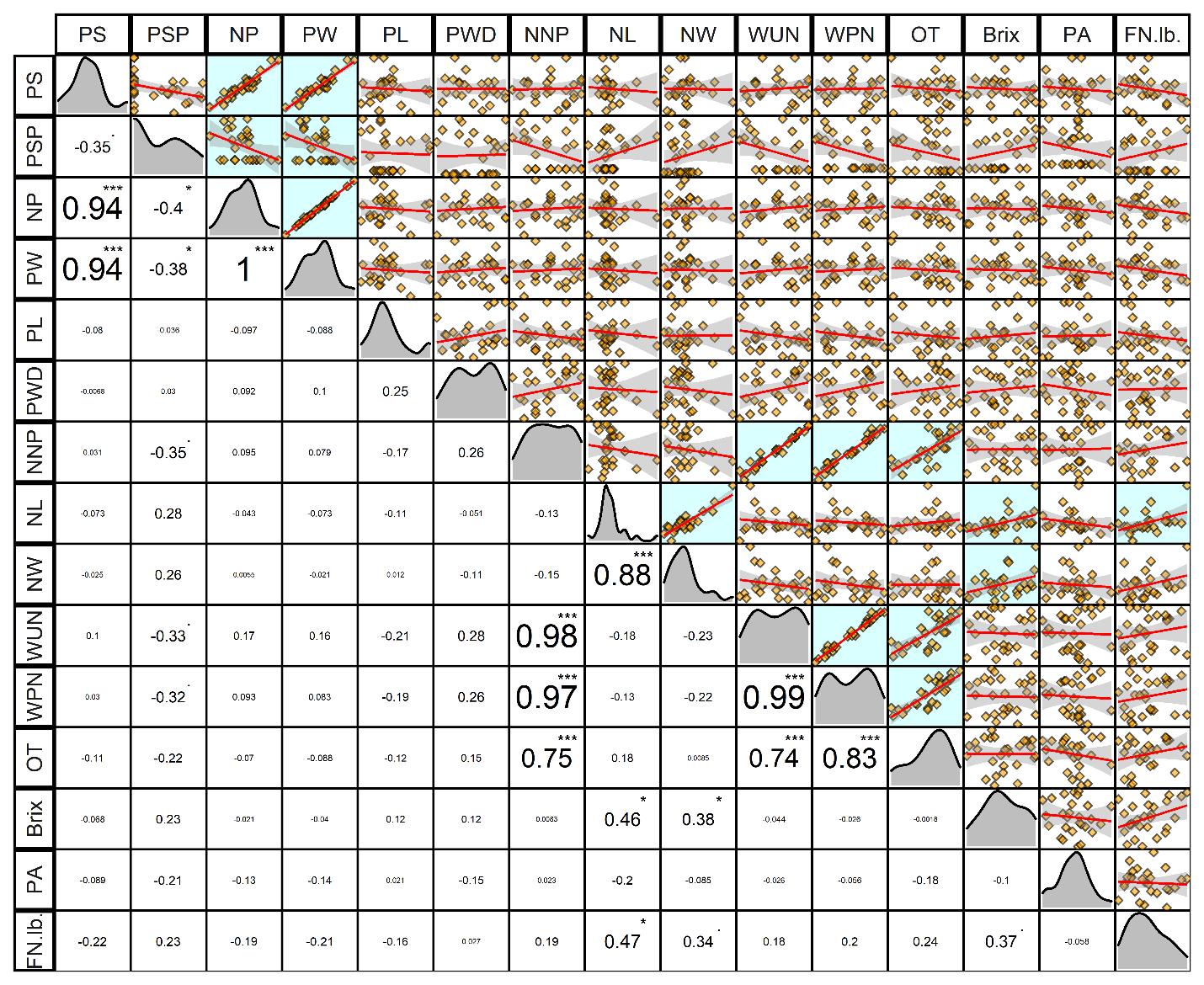


**Supplementary Figure S8.** Correlation among pod set, yield components and nut quality traits of MX2 self-crosses. PS = Pod set (%), PSP =Pseudo pod set (%), NP = number of pods, PW = Pod weight (g), PL = Pod length (cm), PWD =Pod width (cm), NNP = Number of nuts per pod, NL = Nut length (cm), NW = Nut width (cm), WUN = Weight of unpeeled nuts (g), WPN = Weight of peeled nuts (g), OT = Outturn (%), PA= Potential alcohol, FN.Ib = Firmness of nuts (Ib)

**a**

**b**

**Supplementary Figure S9.** Weather conditions in CRIG Tafo in 2019 **(a)** and 2020 **(b)**

ISU = International System of Units

**a**

**b**

**Supplementary Figure S10.** Weather conditions in Afosu during 2019 **(a)** and 2020 **(b)**

**a**

**b**

**Supplementary Figure S11.** Weather conditions at Bunso in 2019 **(a)** and 2020 **(b)**

ISU = International System of Units
