# Supplementary material for: Heterosis patterns and sources of self-compatibility, cross-compatibility and key nut traits within single and double hybrid crosses of kola [Cola nitida (Vent) Schott and Endl.]
Source: Sci Rep. 2023 May 17;13:8036. doi: 10.1038/s41598-023-30485-3 (PMC10192454; doi:10.1038/s41598-023-30485-3)
Supplement: Supplementary file 3 — Supplementary Information 3. [file 41598_2023_30485_MOESM3_ESM.docx]

**Supplementary Table S1.** Variation in pod set, yield and nut quality traits among Bunso progeny crosses.

| Code | Crosses | PS | PSP | NP | PW | NNP | WUN | WPN | OT | Brix | PA | FN |
| --- | --- | --- | --- | --- | --- | --- | --- | --- | --- | --- | --- | --- |
| DCC2 | B1/11 × B1/71 × B1/151×B1/180 | 72.3 | 12.1 | 49.0 | 213.5 | 6.0 | 139.6 | 121.7 | 87.1 | 14.5 | 13.6 | 13.3 |
| DCC5 | B1/11 × B1/71 × B2/177 × B2/156 | 78.1 | 0.0 | 64.9 | 282.6 | 8.0 | 184.7 | 166.9 | 90.3 | 14.3 | 14.6 | 14.4 |
| DCS7 | B1/11 × B1/71 × GX1/46 × GX1/53 | 84.0 | 0.0 | 60.6 | 263.8 | 7.4 | 172.4 | 154.6 | 89.6 | 14.5 | 18.3 | 13.6 |
| DCC5 | B1/11 × B1/71 × B1/11 × B1/71 | 26.9 | 25.4 | 17.3 | 75.4 | 3.9 | 49.3 | 31.4 | 52.9 | 14.7 | 14.9 | 13.0 |
| DCC4 | B1/11 × B1/71 × B1/296 × B1/177 | 82.1 | 4.8 | 31.7 | 138.2 | 3.9 | 90.3 | 72.5 | 80.2 | 14.3 | 10.3 | 14.4 |
| DCC6 | B1/11 × B1/71 × GX1/46 × GX1/16 | 90.0 | 0.0 | 72.1 | 314.0 | 8.9 | 205.2 | 187.4 | 91.3 | 13.2 | 14.2 | 14.8 |
| DCS3 | B1/11 × B1/71 × B1/157 × B1/149 | 81.8 | 0.0 | 69.2 | 301.4 | 8.5 | 197.0 | 179.2 | 90.8 | 14.4 | 15.1 | 12.8 |
| DCC6 | B1/120 × B1/193 × B1/120 × B1/193 | 40.4 | 16.7 | 26.0 | 113.0 | 5.4 | 73.9 | 56.1 | 73.9 | 14.4 | 16.6 | 16.1 |
| DCC1 | B1/120 × B1/193 × GX1/46 × GX1/53 | 82.9 | 0.0 | 62.0 | 270.0 | 7.6 | 176.5 | 158.7 | 89.9 | 14.0 | 17.2 | 13.8 |
| DCS8 | B1/120 × B1/193 × JX1/9 × JX1/11 | 86.9 | 0.0 | 56.2 | 244.9 | 6.9 | 160.1 | 142.2 | 88.9 | 14.5 | 12.1 | 14.7 |
| DCS7 | B1/151 × B1/147 × B1/151 × B1/147 | 32.6 | 11.1 | 21.6 | 94.2 | 2.7 | 61.6 | 43.7 | 67.4 | 15.5 | 14.0 | 15.8 |
| DCC9 | B1/151 × B1/147 × GX1/46 × GX1/53 | 87.7 | 0.0 | 60.6 | 263.8 | 7.4 | 172.4 | 154.6 | 89.7 | 17.3 | 10.8 | 14.5 |
| DCC10 | B1/151 × B1/149 × B1/11 × B1/71 | 84.9 | 0.0 | 49.0 | 213.5 | 6.0 | 139.6 | 121.7 | 87.1 | 12.8 | 12.0 | 15.4 |
| DCS8 | B1/151 × B1/149 × B1/151 × B1/149 | 28.2 | 0.0 | 18.7 | 81.6 | 4.1 | 53.4 | 35.5 | 63.8 | 13.9 | 12.3 | 15.7 |
| DCC11 | B1/151 × B1/149 × B2/177 × B2/156 | 59.1 | 0.0 | 33.2 | 144.4 | 4.1 | 94.4 | 76.6 | 66.7 | 16.3 | 16.7 | 15.8 |
| DCS1 | B1/151 × B1/180 × B1/151 × B1/180 | 27.4 | 24.4 | 17.3 | 75.4 | 5.2 | 49.3 | 31.4 | 62.2 | 16.2 | 16.9 | 14.0 |
| DCS9 | B1/151 × B1/180 × B1/151 × B1/180 | 24.3 | 17.8 | 14.4 | 62.8 | 3.9 | 41.0 | 23.2 | 50.1 | 16.0 | 14.4 | 14.1 |
| DCS10 | B1/157 × B1/149 × B1/157 × B1/149 | 31.8 | 0.0 | 18.0 | 78.5 | 5.7 | 51.3 | 33.5 | 60.8 | 15.7 | 14.2 | 13.0 |
| DCS11 | B1/208 × B1/180 × B1/208 × B1/180 | 36.3 | 16.7 | 24.5 | 106.8 | 4.7 | 69.8 | 51.9 | 73.0 | 14.1 | 14.0 | 11.4 |
| DCC12 | B1/208 × B1/180 × JX1/24 × JX1/22 | 74.3 | 0.0 | 50.5 | 219.8 | 6.2 | 143.7 | 125.8 | 87.4 | 16.1 | 13.0 | 17.6 |
| DCC13 | B1/211 × B1/200 × B1/157 × B1/149 | 82.0 | 3.7 | 38.9 | 169.6 | 4.8 | 110.8 | 93.0 | 83.8 | 13.3 | 12.9 | 13.2 |
| DCS12 | B1/211 × B1/200 × B1/211 × B1/200 | 31.4 | 0.0 | 23.1 | 100.5 | 4.1 | 65.7 | 47.8 | 71.4 | 14.3 | 16.3 | 13.5 |
| DCS13 | B1/212 × B1/210 × B1/212 × B1/210 | 28.5 | 20.8 | 20.9 | 91.1 | 3.4 | 59.5 | 41.7 | 59.3 | 15.1 | 17.3 | 15.7 |
| DCC14 | B1/212 × B1/210 × GX1/46 × GX1/53 | 80.8 | 0.0 | 47.6 | 207.2 | 5.8 | 135.4 | 117.6 | 86.8 | 14.1 | 13.9 | 7.2 |
| DCS14 | B1/212 × B1/236 × B1/212 × B1/236 | 21.3 | 0.0 | 15.9 | 69.1 | 4.2 | 45.1 | 27.3 | 28.7 | 13.5 | 14.7 | 14.1 |
| DCC15 | B1/212 × B1/236 × JX1/24 × JX1/22 | 81.5 | 0.0 | 44.7 | 194.7 | 5.5 | 127.2 | 109.4 | 85.6 | 14.0 | 13.4 | 13.5 |
| DCS2 | B1/212 × B1/236 × B1/212 × B1/236 | 36.6 | 6.7 | 21.6 | 94.2 | 5.0 | 61.6 | 43.7 | 70.2 | 13.8 | 13.2 | 13.8 |
| DCS3 | B1/296 × B1/177 × B1/296 × B1/177 | 28.2 | 0.0 | 18.7 | 81.6 | 4.6 | 53.4 | 35.5 | 56.9 | 16.1 | 15.1 | 16.3 |
| DCC16 | B1/296 × B1/177 × GX1/46 × GX1/53 | 86.5 | 0.0 | 43.3 | 188.4 | 5.3 | 123.1 | 105.3 | 85.4 | 15.4 | 14.4 | 14.9 |
| DCC17 | B2/177 × B2/156 × B1/151 × B1/147 | 44.2 | 22.2 | 30.3 | 131.9 | 3.7 | 86.2 | 68.4 | 70.5 | 14.4 | 13.5 | 14.8 |
| DCS15 | B2/177 × B2/156 × B2/177 × B2/156 | 28.2 | 6.7 | 15.9 | 69.1 | 4.2 | 45.1 | 27.3 | 54.1 | 13.7 | 13.0 | 14.6 |
| DCC18 | B2/177 × B2/156 × JX1/9 × JX1/11 | 80.9 | 0.0 | 60.6 | 263.8 | 7.4 | 172.4 | 154.6 | 89.4 | 15.8 | 15.2 | 11.7 |
| DCS16 | Club × JB 32 × Club × JB 32 | 24.2 | 0.0 | 14.4 | 62.8 | 5.0 | 41.0 | 23.2 | 50.1 | 16.3 | 10.2 | 15.8 |
| DCC19 | Club × JB 32 × JX1/5 × JX1/9 | 97.0 | 0.0 | 83.6 | 364.2 | 10.3 | 238.1 | 220.2 | 92.5 | 13.6 | 14.6 | 15.1 |
| DCC20 | GX1/46 × GX1/16 × B1/151 × B1/180 | 54.2 | 5.6 | 31.7 | 138.2 | 3.9 | 90.3 | 72.5 | 75.9 | 17.3 | 14.2 | 11.8 |
| DCS17 | GX1/46 × GX1/16 × GX1/46 × GX1/16 | 48.6 | 0.0 | 28.8 | 125.6 | 5.3 | 82.1 | 64.3 | 77.4 | 15.8 | 13.4 | 15.5 |
| DCC21 | GX1/46 × GX1/16 × GX1/46 × GX1/53 | 68.8 | 0.0 | 46.1 | 201.0 | 5.7 | 131.3 | 113.5 | 86.4 | 17.1 | 9.1 | 14.1 |
| DCC22 | GX1/46 × GX1/16 × JX1/17 × JX1/9 | 86.1 | 0.0 | 41.8 | 182.1 | 5.1 | 119.0 | 101.2 | 85.0 | 14.6 | 12.3 | 12.9 |
| DCC23 | GX1/46 × GX1/16 × JX1/24 × JX1/22 | 80.7 | 0.0 | 59.1 | 257.5 | 7.3 | 168.3 | 150.5 | 89.4 | 14.3 | 14.2 | 15.0 |
| DCC24 | GX1/46 × GX1/16 × JX1/5 × JX1/9 | 89.2 | 0.0 | 72.1 | 314.0 | 8.9 | 205.2 | 187.4 | 91.2 | 16.6 | 12.5 | 14.1 |
| DCC25 | GX1/46 × GX1/16 × JX1/9 × GX1/16 | 80.5 | 0.0 | 47.6 | 207.2 | 5.8 | 135.4 | 117.6 | 86.8 | 13.9 | 14.3 | 16.5 |
| DCC26 | GX1/46 × GX1/16 × JX1/9 × JX1/11 | 87.4 | 0.0 | 75.0 | 326.6 | 9.2 | 213.4 | 195.6 | 91.0 | 14.4 | 10.9 | 13.8 |
| DCC27 | GX1/46 × GX1/33 × B1/212 × B1/236 | 80.5 | 7.4 | 46.1 | 201.0 | 5.7 | 131.3 | 113.5 | 86.1 | 15.2 | 14.0 | 13.1 |
| DCS18 | GX1/46 × GX1/33 × GX1/46 × GX1/33 | 38.7 | 0.0 | 28.8 | 125.6 | 5.7 | 82.1 | 64.3 | 72.5 | 14.5 | 12.7 | 15.1 |
| DCC28 | GX1/46 × GX1/33 × JX1/24 × JX1/22 | 87.9 | 0.0 | 60.6 | 263.8 | 7.4 | 172.4 | 154.6 | 89.6 | 16.7 | 14.4 | 12.3 |
| DCC29 | GX1/46 × GX1/53 × B2/296 × B1/177 | 87.9 | 0.0 | 51.9 | 226.1 | 6.4 | 147.8 | 129.9 | 87.9 | 11.9 | 14.1 | 14.4 |
| DCC30 | GX1/46 × GX1/53 × GX1/46 × GX1/16 | 66.4 | 0.0 | 49.0 | 213.5 | 6.0 | 139.6 | 121.7 | 86.9 | 15.0 | 13.1 | 15.2 |
| DCC31 | GX1/46 × GX1/53 × GX1/46 × GX1/53 | 56.5 | 0.0 | 39.8 | 173.3 | 5.4 | 113.3 | 95.5 | 78.4 | 14.1 | 12.0 | 12.6 |
| DCC32 | GX1/46 × GX1/53 × JX1/17 × JX1/5 | 89.3 | 0.0 | 47.6 | 207.2 | 5.8 | 135.4 | 117.6 | 86.8 | 15.8 | 15.7 | 13.7 |
| DCC33 | GX1/46 × GX1/53 × JX1/24 × JX1/22 | 68.3 | 11.1 | 46.1 | 201.0 | 5.9 | 131.3 | 113.5 | 84.5 | 14.1 | 17.5 | 12.8 |
| DCC34 | GX1/46 × GX1/53 × JX1/9 × JX1/11 | 77.2 | 0.0 | 53.3 | 232.4 | 6.5 | 151.9 | 134.0 | 88.0 | 16.2 | 15.1 | 17.3 |
| DCS20 | JX1/14 × JX1/32 × JX1/14 × JX1/32 | 37.8 | 8.3 | 27.4 | 119.3 | 4.6 | 78.0 | 60.2 | 75.0 | 15.6 | 12.3 | 14.5 |
| DCC35 | JX1/14 × JX1/32 × JX1/9 × JX1/11 | 66.2 | 0.0 | 56.2 | 244.9 | 6.9 | 160.1 | 142.2 | 87.7 | 14.6 | 11.4 | 15.6 |
| DCS21 | JX1/17 × JX1/5 × JX1/17 × JX1/5 | 40.9 | 16.7 | 26.0 | 113.0 | 3.8 | 73.9 | 56.1 | 75.4 | 14.3 | 10.9 | 16.7 |
| DCC36 | JX1/17 × JX1/5 × JX1/24 × JX1/22 | 77.3 | 0.0 | 49.0 | 213.5 | 6.0 | 139.6 | 121.7 | 87.1 | 14.7 | 15.9 | 15.0 |
| DCC37 | JX1/17 × JX1/5 × JX1/9 × JX1/11 | 84.8 | 0.0 | 62.0 | 270.0 | 7.6 | 176.5 | 158.7 | 89.7 | 15.6 | 15.0 | 14.2 |
| DCC38 | JX1/17 × JX1/9 × B1/212 × B1/210 | 52.5 | 12.5 | 34.6 | 150.7 | 5.6 | 98.5 | 80.7 | 66.4 | 15.1 | 13.1 | 13.4 |
| DCC39 | JX1/17 × JX1/9 × GX1/46 × GX1/16 | 73.1 | 5.6 | 47.6 | 207.2 | 5.8 | 135.4 | 117.6 | 86.6 | 15.6 | 12.9 | 13.6 |
| DCS22 | JX1/17 × JX1/9 × JX1/17 × JX1/9 | 42.2 | 0.0 | 24.5 | 106.8 | 3.4 | 69.8 | 51.9 | 73.0 | 15.2 | 16.9 | 13.7 |
| DCC40 | JX1/17 × JX1/9 × JX1/9 × JX1/11 | 66.9 | 2.6 | 43.3 | 188.4 | 5.3 | 123.1 | 105.3 | 84.9 | 16.9 | 16.0 | 14.0 |
| DCC41 | JX1/23 × JX1/53 × GX1/46 × GX1/16 | 80.1 | 0.0 | 40.4 | 175.8 | 5.0 | 114.9 | 97.1 | 84.3 | 15.2 | 15.3 | 17.4 |
| DCC42 | JX1/23 × JX1/53 × GX1/46 × GX1/53 | 85.6 | 0.0 | 56.2 | 244.9 | 6.9 | 160.1 | 142.2 | 88.8 | 12.9 | 14.4 | 12.2 |
| DCS23 | JX1/23 × JX1/53 × JX1/23 × JX1/53 | 42.3 | 0.0 | 24.5 | 106.8 | 3.1 | 69.8 | 51.9 | 73.0 | 14.0 | 16.3 | 15.9 |
| DCC43 | JX1/24 × JX1/22 × B1/151 × B1/147 | 71.5 | 0.0 | 40.4 | 175.8 | 5.0 | 114.9 | 97.1 | 83.9 | 14.5 | 17.3 | 14.3 |
| DCC44 | JX1/24 × JX1/22 × GX1/46 × GX1/16 | 77.2 | 0.0 | 43.3 | 188.4 | 5.3 | 123.1 | 105.3 | 85.4 | 15.6 | 12.1 | 17.3 |
| DCC45 | JX1/24 × JX1/22 × GX1/46 × GX1/53 | 79.4 | 0.0 | 49.0 | 213.5 | 6.0 | 139.6 | 121.7 | 87.2 | 15.8 | 16.1 | 12.9 |
| DCS24 | JX1/24 × JX1/22 × JX1/24 × JX1/22 | 37.6 | 0.0 | 24.5 | 106.8 | 4.9 | 69.8 | 51.9 | 73.0 | 16.8 | 16.4 | 14.0 |
| DCC46 | JX1/24 × JX1/22 × JX1/7 × JX1/53 | 73.0 | 4.2 | 41.8 | 182.1 | 5.1 | 119.0 | 101.2 | 84.8 | 14.4 | 14.2 | 13.5 |
| DCC47 | JX1/5 × JX1/9 × GX1/46 × GX1/16 | 68.2 | 0.0 | 46.1 | 201.0 | 5.7 | 131.3 | 113.5 | 81.0 | 15.1 | 10.5 | 14.5 |
| DCS25 | JX1/5 × JX1/9 × JX1/5 × JX1/9 | 32.4 | 8.3 | 23.1 | 100.5 | 3.1 | 65.7 | 47.8 | 69.8 | 12.4 | 15.3 | 14.1 |
| DCC48 | JX1/5 × JX1/9 × JX1/9 × JX1/11 | 82.6 | 0.0 | 54.8 | 238.6 | 6.7 | 156.0 | 138.1 | 88.6 | 15.9 | 13.4 | 14.8 |
| DCS4 | JX1/7 × JX1/5 × JX1/7 × JX1/5 | 35.0 | 22.2 | 20.2 | 87.9 | 5.3 | 57.5 | 39.6 | 66.2 | 15.4 | 16.7 | 13.7 |
| DCC49 | JX1/7 × JX1/53 × JX1/7 × JX1/5 | 85.8 | 0.0 | 53.3 | 232.4 | 6.5 | 151.9 | 134.0 | 88.0 | 15.8 | 14.3 | 13.0 |
| DCS26 | JX1/7 × JX1/53 × JX1/7 × JX1/53 | 42.2 | 0.0 | 28.8 | 125.6 | 4.0 | 82.1 | 64.3 | 77.4 | 17.7 | 12.2 | 15.5 |
| DCS27 | JX1/9 × GX1/16 × JX1/9 × GX1/16 | 44.3 | 0.0 | 28.8 | 125.6 | 4.1 | 82.1 | 64.3 | 77.4 | 14.4 | 14.7 | 16.9 |
| DCC50 | JX1/9 × JX1/11 × GX1/46 × GX1/53 | 90.0 | 0.0 | 50.5 | 219.8 | 6.2 | 143.7 | 125.8 | 87.3 | 15.5 | 12.8 | 16.1 |
| DCC51 | JX1/9 × JX1/11 × JX1/17 × JX1/5 | 78.7 | 0.0 | 38.9 | 169.6 | 4.8 | 110.8 | 93.0 | 83.1 | 18.5 | 17.6 | 13.8 |
| DCC52 | JX1/9 × JX1/11 × JX1/24 × JX1/22 | 79.0 | 0.0 | 41.8 | 182.1 | 5.1 | 119.0 | 101.2 | 84.9 | 14.5 | 14.7 | 15.6 |
| DCC53 | JX1/9 × JX1/11 × JX1/5 × JX1/9 | 78.2 | 9.1 | 41.8 | 182.1 | 5.1 | 119.0 | 101.2 | 84.8 | 15.3 | 12.5 | 13.3 |
| DCC54 | JX1/9 × JX1/11 × JX1/7 × JX1/5 | 65.0 | 0.0 | 44.7 | 194.7 | 6.1 | 127.2 | 109.4 | 80.8 | 13.5 | 14.3 | 15.5 |
| DCC55 | JX1/9 × JX1/11 × JX1/7 × JX1/53 | 82.5 | 0.0 | 47.6 | 207.2 | 5.8 | 135.4 | 117.6 | 86.8 | 13.9 | 14.8 | 13.4 |
| DCS28 | JX1/9 × JX1/11 × JX1/9 × JX1/11 | 42.7 | 12.7 | 26.0 | 113.0 | 4.5 | 73.9 | 56.1 | 74.8 | 15.5 | 11.8 | 13.1 |
|  | S.e  Mean | 13.5  61.7 | 12.2  4.4 | 10.1  39.9 | 43.8  173.9 | 1.2  5.5 | 28.6  113.6 | 28.7  95.8 | 12.5  78.5 | 2.7  14.9 | 3.1  14.1 | 2.5  14.3 |

PS = Pod set (%), PSP = Pseudo pod set (%), NP= Number of pods. PW = Pod weight, NNP = Number of nuts / pods, WUN (g) = Weight of unpeeled nuts (g), OT = Outturn (%), PA = potential alcohol, FN = firmness of nuts (lb), DCC = Double hybrid cross, DCS = Double hybrid self-cross

**Supplementary Table S2.** Variation in pod set, yield and nut quality traits among JX1 crosses.

| Code | Crosses | PS | PSP | NP | PW | NNP | WUN | WPN | OT | Brix | PA | FN |
| --- | --- | --- | --- | --- | --- | --- | --- | --- | --- | --- | --- | --- |
| SCC15 | JX1/1 × JX1/112 | 71.4 | 26.7 | 31.2 | 165.7 | 5.5 | 88.4 | 58.3 | 66.2 | 14.7 | 10.2 | 12.9 |
| SCC16 | JX1/1 × JX1/67 | 80.9 | 0.0 | 41.6 | 221.0 | 7.3 | 117.8 | 84.4 | 72.0 | 13.9 | 11.4 | 16.2 |
| SCS56 | JX1/10 × JX1/10 | 18.7 | 16.7 | 11.7 | 62.2 | 2.0 | 33.1 | 24.1 | 73.2 | 12.3 | 8.2 | 11.8 |
| SCC3 | JX1/10 × JX1/36 | 69.6 | 0.0 | 35.1 | 186.5 | 6.1 | 99.4 | 64.9 | 65.7 | 12.6 | 10.7 | 13.5 |
| SCC4 | JX1/10 × JX1/48 | 75.9 | 0.0 | 42.9 | 227.9 | 7.5 | 121.5 | 75.1 | 61.6 | 12.4 | 9.0 | 14.3 |
| SCC1 | JX1/108 × JX1/23 | 68.1 | 0.0 | 33.8 | 179.5 | 5.9 | 95.7 | 59.0 | 61.7 | 13.5 | 11.2 | 16.4 |
| SCC2 | JX1/108 × JX1/6 | 75.0 | 0.0 | 37.7 | 200.3 | 6.6 | 106.8 | 68.8 | 64.0 | 15.0 | 12.1 | 15.2 |
| SCS55 | JX1/108 × JX1/108 | 44.4 | 0.0 | 22.1 | 117.4 | 3.9 | 62.6 | 40.2 | 63.4 | 11.1 | 7.3 | 14.2 |
| SCS59 | JX1/11 × JX1/11 | 0.0 | 0.0 | - | - | - | - | - | - | - | - | - |
| SCC14 | JX1/11 × JX1/23 | 63.5 | 4.2 | 32.5 | 172.6 | 5.7 | 92.1 | 70.6 | 75.1 | 12.9 | 9.0 | 14.3 |
| SCC5 | JX1/112 × JX1/23 | 3.3 | 0.0 | 1.3 | 6.9 | 0.2 | 3.7 | 2.8 | 24.9 | 4.8 | 3.6 | 4.1 |
| SCC6 | JX1/117 × JX1/73 | 52.5 | 40.3 | 33.8 | 179.5 | 5.9 | 95.7 | 64.3 | 68.9 | 15.9 | 7.4 | 16.5 |
| SCS57 | JX1/118 × JX1/118 | 26.4 | 11.1 | 14.3 | 76.0 | 2.5 | 40.5 | 27.1 | 68.5 | 14.1 | 7.7 | 16.1 |
| SCC7 | JX1/118 × JX1/23 | 70.6 | 8.3 | 35.1 | 186.5 | 6.1 | 99.4 | 60.4 | 60.5 | 12.5 | 12.3 | 14.2 |
| SCC8 | JX1/118 × JX1/36 | 16.2 | 0.0 | 7.8 | 41.4 | 1.4 | 22.1 | 14.1 | 61.2 | 17.0 | 10.0 | 12.8 |
| SCC9 | JX1/118 × JX1/6 | 67.0 | 14.8 | 39.0 | 207.2 | 6.8 | 110.5 | 69.6 | 62.7 | 14.1 | 10.2 | 11.7 |
| SCC10 | JX1/118 × JX1/8 | 12.5 | 27.8 | 6.5 | 34.5 | 1.1 | 18.4 | 11.4 | 40.0 | 12.2 | 7.4 | 10.4 |
| SCS58 | JX1/119 × JX1/ 119 | 20.1 | 17.8 | 15.6 | 82.9 | 2.7 | 44.2 | 31.6 | 73.0 | 10.7 | 8.9 | 13.0 |
| SCC12 | JX1/119 × JX1/23 | 60.3 | 0.0 | 35.1 | 186.5 | 6.1 | 99.4 | 64.3 | 64.5 | 12.9 | 7.6 | 14.8 |
| SCC13 | JX1/119 × JX1/32 | 75.2 | 0.0 | 35.1 | 186.5 | 6.1 | 99.4 | 60.6 | 61.8 | 17.1 | 5.9 | 14.6 |
| SCC11 | JX1/119 × JX1/50 | 74.2 | 0.0 | 36.4 | 193.4 | 6.4 | 103.1 | 62.9 | 61.4 | 11.5 | 8.5 | 14.4 |
| SCS60 | JX1/122 × JX1/122 | 0.0 | 0.0 | - | - | - | - | - | - | - | - | - |
| SCS61 | JX1/199 × JX1/199 | 30.6 | 63.3 | 16.9 | 89.8 | 3.0 | 47.9 | 31.8 | 69.8 | 10.7 | 5.9 | 13.1 |
| SCS68 | JX1/2 × JX1/2 | 45.6 | 0.0 | 27.3 | 145.0 | 4.8 | 77.3 | 54.2 | 70.2 | 11.7 | 7.3 | 16.3 |
| SCC32 | JX1/2 × JX1/25 | 13.3 | 11.1 | 7.8 | 41.4 | 1.4 | 22.1 | 14.9 | 22.4 | 5.5 | 2.6 | 8.2 |
| SCC33 | JX1/2 × JX1/27 | 65.6 | 0.0 | 35.1 | 186.5 | 6.1 | 99.4 | 64.6 | 65.0 | 11.2 | 9.8 | 14.4 |
| SCC34 | JX1/2 × JX1/45 | 80.5 | 0.0 | 35.1 | 186.5 | 6.1 | 99.4 | 62.4 | 63.6 | 15.4 | 9.6 | 13.0 |
| SCC35 | JX1/2 × JX1/54 | 65.7 | 0.0 | 33.8 | 179.5 | 5.9 | 95.7 | 58.8 | 61.2 | 13.1 | 10.9 | 16.1 |
| SCC36 | JX1/2 × JX1/6 | 73.7 | 0.0 | 39.0 | 207.2 | 6.8 | 110.5 | 68.2 | 61.8 | 15.1 | 8.9 | 12.8 |
| SCC37 | JX1/2 × JX1/7 | 71.1 | 0.0 | 40.3 | 214.1 | 7.1 | 114.2 | 70.7 | 59.7 | 13.4 | 8.5 | 13.1 |
| SCC18 | JX1/20 × JX1/118 | 87.9 | 0.0 | 48.1 | 255.5 | 8.4 | 136.2 | 87.1 | 64.1 | 16.1 | 9.3 | 13.8 |
| SCC19 | JX1/20 × JX1/119 | 64.4 | 20.8 | 35.1 | 186.5 | 6.1 | 99.4 | 63.7 | 63.6 | 15.3 | 9.4 | 16.2 |
| SCS62 | JX1/20 × JX1/20 | 31.6 | 28.3 | 19.5 | 103.6 | 3.4 | 55.2 | 36.0 | 64.0 | 9.1 | 6.1 | 15.7 |
| SCC20 | JX1/20 × JX1/31 | 83.8 | 0.0 | 44.2 | 234.8 | 7.7 | 125.2 | 78.3 | 62.4 | 16.0 | 11 | 13.7 |
| SCC21 | JX1/20 × JX1/34 | 31.0 | 21.7 | 15.6 | 82.9 | 2.7 | 44.2 | 38.2 | 85.2 | 16.6 | 7.7 | 12.6 |
| SCC22 | JX1/20 × JX1/50 | 16.5 | 8.3 | 9.1 | 48.3 | 1.6 | 25.8 | 16.4 | 67.2 | 12.2 | 8.9 | 15.1 |
| SCC17 | JX1/20 × JX1/23 | 45.3 | 11.1 | 27.3 | 145.0 | 4.8 | 77.3 | 50.0 | 64.5 | 15.0 | 11.1 | 14.3 |
| SCS63 | JX1/21 × JX1/21 | 41.3 | 0.0 | 24.7 | 131.2 | 4.3 | 70.0 | 42.0 | 60.1 | 13.8 | 8.0 | 15.0 |
| SCC23 | JX1/21 × JX1/9 | 89.8 | 0.0 | 49.4 | 262.4 | 8.7 | 139.9 | 85.3 | 61.0 | 14.1 | 8.1 | 14.2 |
| SCS64 | JX1/22 × JX1/22 | 16.9 | 0.0 | 9.1 | 48.3 | 1.6 | 25.8 | 18.5 | 71.9 | 14.1 | 8.3 | 12.4 |
| SCC24 | JX1/22 × JX1/34 | 65.8 | 3.3 | 36.4 | 193.4 | 6.4 | 103.1 | 69.3 | 67.5 | 16.0 | 6.8 | 14.6 |
| SCS65 | JX1/23 × JX1/23 | 0.0 | 0.0 | - | - | - | - | - | - | - | - | - |
| SCC25 | JX1/23 × JX1/34 | 7.1 | 22.2 | 3.9 | 20.7 | 0.7 | 11.1 | 6.7 | 20.3 | 5.5 | 4.1 | 5.7 |
| SCS66 | JX1/24 × JX1/24 | 20.2 | 25.0 | 13.0 | 69.1 | 2.3 | 36.8 | 26.2 | 70.9 | 14.4 | 8.8 | 16.3 |
| SCC26 | JX1/24 × JX1/45 | 46.0 | 16.7 | 27.3 | 145.0 | 4.8 | 77.3 | 51.6 | 66.8 | 17.3 | 10.0 | 11.6 |
| SCC27 | JX1/24 × JX1/6 | 70.0 | 0.0 | 39.0 | 207.2 | 6.8 | 110.5 | 74.6 | 67.5 | 13.6 | 8.5 | 13.0 |
| SCC28 | JX1/25 × JX1/99 | 18.6 | 16.7 | 7.8 | 41.4 | 1.4 | 22.1 | 35.9 | 66.6 | 16.7 | 11.1 | 13.8 |
| SCC29 | JX1/27 × JX1/25 | 72.6 | 0.0 | 41.6 | 221.0 | 7.3 | 117.8 | 76.9 | 65.2 | 16.9 | 13.2 | 14.4 |
| SCS67 | JX1/27 × JX1/27 | 16.0 | 0.0 | 9.1 | 48.3 | 1.6 | 25.8 | 16.1 | 60.9 | 14.9 | 8.1 | 14.6 |
| SCC30 | JX1/27 × JX1/31 | 74.7 | 0.0 | 36.4 | 193.4 | 6.4 | 103.1 | 67.0 | 65.1 | 13.4 | 9.1 | 15.9 |
| SCC31 | JX1/27 × JX1/89 | 73.2 | 0.0 | 37.7 | 200.3 | 6.6 | 106.8 | 64.4 | 60.8 | 12.9 | 11.1 | 13.4 |
| SCS69 | JX1/30 × JX1/30 | 13.9 | 44.4 | 9.1 | 48.3 | 1.6 | 25.8 | 18.5 | 72.1 | 13.5 | 7.3 | 13.6 |
| SCC38 | JX1/30 × JX1/45 | 72.4 | 20.0 | 50.7 | 269.3 | 8.9 | 143.6 | 91.1 | 63.7 | 16.4 | 10.8 | 15.3 |
| SCC39 | JX1/30 × JX1/51 | 3.9.0 | 0.0 | 2.6 | 13.8 | 0.5 | 7.4 | 4.6 | 20.7 | 3.1 | 4.6 | 5.4 |
| SCC40 | JX1/30 × JX1/7 | 63.2 | 0.0 | 36.4 | 193.4 | 6.4 | 103.1 | 65.6 | 63.8 | 18.1 | 10.2 | 17.0 |
| SCC41 | JX1/31 × JX1/23 | 73.1 | 0.0 | 35.1 | 186.5 | 6.1 | 99.4 | 67.8 | 68.4 | 17.6 | 11.5 | 15.3 |
| SCC42 | JX1/31 × JX1/27 | 69.1 | 6.7 | 40.3 | 214.1 | 7.1 | 114.2 | 74.4 | 65.2 | 9.8 | 7.6 | 13.1 |
| SCC43 | JX1/31 × JX1/28 | 71.8 | 0.0 | 39.0 | 207.2 | 6.8 | 110.5 | 69.5 | 62.8 | 16.1 | 12.0 | 12.2 |
| SCS70 | JX1/31 × JX1/31 | 62.5 | 12.5 | 36.4 | 193.4 | 6.4 | 103.1 | 67.2 | 65.2 | 14.7 | 7.9 | 14.2 |
| SCC44 | JX1/31 × JX1/50 | 14.2 | 27.8 | 6.5 | 34.5 | 1.1 | 18.4 | 12.0 | 43.6 | 5.1 | 8.2 | 9.6 |
| SCS71 | JX1/32 × JX1/32 | 13.3 | 0.0 | 6.5 | 34.5 | 2.9 | 18.4 | 13.4 | 74.0 | 11.0 | 7.8 | 16.0 |
| SCS72 | JX1/33 × JX1/33 | 41.9 | 5.6 | 20.8 | 110.5 | 3.6 | 58.9 | 39.2 | 66.7 | 14.1 | 7.4 | 16.4 |
| SCC45 | JX1/34 × JX1/23 | 16.3 | 8.3 | 9.1 | 48.3 | 1.6 | 25.8 | 18.4 | 68.1 | 14.2 | 9.4 | 13.5 |
| SCS73 | JX1/34 × JX1/34 | 28.3 | 0.0 | 15.6 | 82.9 | 2.7 | 44.2 | 29.9 | 67.6 | 9.8 | 6.4 | 14.1 |
| SCC46 | JX1/34 × JX1/48 | 59.6 | 7.4 | 32.5 | 172.6 | 5.7 | 92.1 | 59.3 | 64.4 | 15.2 | 10.3 | 13.1 |
| SCC47 | JX1/35 × JX1/23 | 70.6 | 0.0 | 39.0 | 207.2 | 6.8 | 110.5 | 74.4 | 67.1 | 12.6 | 9.6 | 14.5 |
| SCS74 | JX1/36 × JX1/36 | 14.7 | 33.3 | 9.1 | 48.3 | 1.6 | 25.8 | 16.9 | 67.4 | 13.0 | 6.8 | 14.7 |
| SCS78 | JX1/4 × JX1/4 | 42.6 | 13.3 | 24.7 | 131.2 | 4.3 | 70.0 | 46.0 | 65.4 | 12.4 | 6.6 | 11.9 |
| SCS75 | JX1/42 × JX1/42 | 42.0 | 12.5 | 26.0 | 138.1 | 4.6 | 73.6 | 44.2 | 60.6 | 14.4 | 8.3 | 14.9 |
| SCS76 | JX1/45 × JX1/45 | 15.4 | 0.0 | 9.1 | 48.3 | 2.4 | 25.8 | 17.5 | 69.1 | 14.0 | 7.3 | 17.0 |
| SCC48 | JX1/45 × JX1/6 | 73.2 | 0.0 | 36.4 | 193.4 | 6.4 | 103.1 | 66.3 | 64.1 | 11.7 | 11.8 | 14.3 |
| SCS77 | JX1/49 × JX1/49 | 0.0 | 0.0 | - | - | - | - | - | - | - | - | - |
| SCC59 | JX1/5 × JX1/9 | 50.1 | 16.7 | 31.2 | 165.7 | 5.5 | 88.4 | 56.0 | 63.4 | 19.4 | 12.2 | 16.5 |
| SCC49 | JX1/50 × JX1/22 | 14.4 | 0.0 | 7.8 | 41.4 | 1.4 | 22.1 | 14.9 | 65.5 | 12.6 | 10.5 | 15.4 |
| SCC50 | JX1/50 × JX1/31 | 75.1 | 0.0 | 37.7 | 200.3 | 6.6 | 106.8 | 71.1 | 66.3 | 12.5 | 10.5 | 11.1 |
| SCS79 | JX1/50 × JX1/50 | 16.2 | 11.1 | 9.1 | 48.3 | 2.1 | 25.8 | 16.8 | 64.1 | 13.7 | 9.5 | 12.7 |
| SCC53 | JX1/51 × JX1/20 | 87.1 | 0.0 | 50.7 | 269.3 | 8.9 | 143.6 | 85.5 | 59.8 | 17.1 | 9.3 | 14.8 |
| SCC54 | JX1/51 × JX1/25 | 65.2 | 9.5 | 35.1 | 186.5 | 6.1 | 99.4 | 63.9 | 64.8 | 18.8 | 12.6 | 15.4 |
| SCC55 | JX1/51 × JX1/34 | 25.4 | 0.0 | 15.6 | 82.9 | 2.7 | 44.2 | 29.4 | 65.7 | 14.7 | 12.6 | 15.4 |
| SCC56 | JX1/51 × JX1/36 | 77.0 | 0.0 | 35.1 | 186.5 | 6.1 | 99.4 | 62.0 | 62.7 | 17.6 | 10.4 | 14.2 |
| SCC57 | JX1/51 × JX1/42 | 76.9 | 0.0 | 39.0 | 207.2 | 6.8 | 110.5 | 66.7 | 60.7 | 17.1 | 9.5 | 12.8 |
| SCS80 | JX1/51 × JX1/51 | 0.0 | 0.0 | - | - | - | - | - | - | - | - | - |
| SCC58 | JX1/51 × JX1/ATTA 2 | 72.9 | 0.0 | 40.3 | 214.1 | 7.1 | 114.2 | 67.9 | 59.7 | 15.3 | 8.7 | 11.4 |
| SCC51 | JX1/51 × JX1/31 | 61.3 | 16.7 | 32.5 | 172.6 | 5.7 | 92.1 | 59.6 | 64.6 | 17.3 | 10.0 | 14.3 |
| SCC52 | JX1/51 × JX1/23 | 90.8 | 0.0 | 48.1 | 255.5 | 8.4 | 136.2 | 86.8 | 63.8 | 14.3 | 11.5 | 16.8 |
| SCS81 | JX1/58 × JX1/58 | 34.6 | 16.7 | 20.8 | 110.5 | 3.6 | 58.9 | 38.8 | 67.4 | 12.5 | 7.2 | 14.9 |
| SCC72 | JX1/6 × JX1/113 | 78.1 | 0.0 | 44.2 | 234.8 | 7.7 | 125.2 | 76.3 | 61.1 | 14.8 | 11.0 | 12.9 |
| SCC73 | JX1/6 × JX1/27 | 2.2 | 0.0 | 1.3 | 6.9 | 0.2 | 3.7 | 2.8 | 25.0 | 4.2 | 3.8 | 3.4 |
| SCC74 | JX1/6 × JX1/54 | 53.9 | 0.0 | 28.6 | 151.9 | 5.0 | 81 | 50.5 | 62.2 | 16.6 | 9.2 | 15.2 |
| SCS86 | JX1/6 × JX1/6 | 10.8 | 0.0 | 5.2 | 27.6 | 0.9 | 14.7 | 10.0 | 45.4 | 12.1 | 3.8 | 9.8 |
| SCC75 | JX1/6 × JX1/7 | 63.2 | 0.0 | 35.1 | 186.5 | 6.1 | 99.4 | 61.4 | 61.5 | 14.4 | 10.5 | 13.8 |
| SCS82 | JX1/61 × JX1/61 | 29.8 | 17.8 | 16.9 | 89.8 | 3.0 | 47.9 | 32.8 | 69.5 | 13.5 | 9.1 | 11.4 |
| SCC60 | JX1/62 × JX1/27 | 41.8 | 0.0 | 24.7 | 131.2 | 4.3 | 70.0 | 44.4 | 63.3 | 14.3 | 11.7 | 17.7 |
| SCC61 | JX1/62 × JX1/51 | 4.8 | 0.0 | 2.6 | 13.8 | 0.5 | 7.4 | 5.1 | 23.2 | 5.8 | 1.7 | 5.5 |
| SCC62 | JX1/62 × JX1/54 | 84.4 | 0.0 | 54.6 | 290.0 | 9.6 | 154.7 | 95.7 | 61.9 | 12.7 | 7.3 | 16.3 |
| SCC63 | JX1/62 × JX1/6 | 57.6 | 14.3 | 31.2 | 165.7 | 5.5 | 88.4 | 54.2 | 61.1 | 17.1 | 8.3 | 14.1 |
| SCS83 | JX1/62 × JX1/62 | 15.8 | 11.1 | 9.1 | 48.3 | 3.1 | 25.8 | 19.1 | 69.0 | 15.2 | 7.7 | 13.9 |
| SCC64 | JX1/62 × JX1/7 | 78.9 | 0.0 | 41.6 | 221.0 | 7.3 | 117.8 | 71.3 | 60.7 | 15.8 | 9.9 | 17.2 |
| SCC66 | JX1/63 × JX1/113 | 72.3 | 0.0 | 40.3 | 214.1 | 7.1 | 114.2 | 74.3 | 65.1 | 15.0 | 11.1 | 18.0 |
| SCC67 | JX1/63 × JX1/23 | 87.1 | 0.0 | 50.7 | 269.3 | 8.9 | 143.6 | 85.0 | 59.5 | 17.3 | 11.3 | 15.1 |
| SCC68 | JX1/63 × JX1/4 | 54.4 | 6.7 | 31.2 | 165.7 | 5.5 | 88.4 | 63.9 | 70.6 | 13.4 | 11.5 | 14.6 |
| SCC69 | JX1/63 × JX1/6 | 18.1 | 27.8 | 10.4 | 55.2 | 1.8 | 29.5 | 19.0 | 64.9 | 15.9 | 10.8 | 16.7 |
| SCS84 | JX1/63 × JX1/63 | 27.2 | 20.0 | 15.6 | 82.9 | 2.7 | 44.2 | 28.7 | 65.3 | 11.8 | 7.1 | 15.0 |
| SCC65 | JX1/63 × JX1/112 | 67.1 | 0.0 | 40.3 | 214.1 | 7.1 | 114.2 | 70.7 | 62.0 | 15.9 | 12.7 | 14.9 |
| SCC70 | JX1/66 × JX1/23 | 75.2 | 0.0 | 39.0 | 207.2 | 6.8 | 110.5 | 74.3 | 67.0 | 14.0 | 14.6 | 11.6 |
| SCC71 | JX1/66 × JX1/34 | 74.6 | 0.0 | 41.6 | 221.0 | 7.3 | 117.8 | 74.2 | 63.0 | 16.9 | 9.5 | 13.7 |
| SCS85 | JX1/66 × JX1/66 | 16.1 | 11.1 | 9.1 | 48.3 | 3.1 | 25.8 | 15.6 | 60.0 | 8.6 | 8.1 | 16.7 |
| SCS89 | JX1/7 × JX1/7 | 35.9 | 16.7 | 18.2 | 96.7 | 3.2 | 51.6 | 33.3 | 65.2 | 13.3 | 9.3 | 14.8 |
| SCC76 | JX1/73 × JX1/23 | 86.8 | 0.0 | 53.3 | 283.1 | 9.3 | 151.0 | 98.7 | 64.5 | 17.9 | 12.6 | 11.8 |
| SCC77 | JX1/73 × JX1/31 | 46.6 | 23.8 | 27.3 | 145.0 | 4.8 | 77.3 | 49.9 | 64.1 | 14.7 | 10.8 | 12.9 |
| SCC78 | JX1/73 × JX1/36 | 0.0 | 0.0 | - | - | - | - | - | - | - | - | - |
| SCC79 | JX1/73 × JX1/6 | 89.7 | 0.0 | 44.2 | 234.8 | 7.7 | 125.2 | 79.2 | 63.7 | 16.9 | 11.1 | 16.1 |
| SCC80 | JX1/73 × JX1/63 | 70.0 | 0.0 | 40.3 | 214.1 | 7.1 | 114.2 | 75.5 | 66.1 | 16.9 | 10.1 | 14.3 |
| SCS87 | JX1/73 × JX1/73 | 22.5 | 0.0 | 13.0 | 69.1 | 2.3 | 36.8 | 23.6 | 62.2 | 12.9 | 8.5 | 13.3 |
| SCC81 | JX1/74 × JX1/108 | 13.6 | 22.2 | 7.8 | 41.4 | 1.4 | 22.1 | 14.6 | 64.4 | 14.0 | 13.5 | 14.5 |
| SCS88 | JX1/77 × JX1/77 | 14.1 | 30.0 | 9.1 | 48.3 | 1.6 | 25.8 | 16.6 | 42.3 | 11.3 | 5.5 | 11.9 |
| SCC82 | JX1/8 × JX1/112 | 72.7 | 0.0 | 37.7 | 200.3 | 6.6 | 106.8 | 69.9 | 65.4 | 12.8 | 10.3 | 10.5 |
| SCC87 | JX1/8 × JX1/113 | 34.3 | 16.7 | 18.9 | 100.1 | 3.3 | 53.4 | 36.3 | 46.3 | 10.2 | 8.3 | 11.3 |
| SCC88 | JX1/8 × JX1/118 | 74.3 | 5.0 | 41.0 | 217.5 | 7.2 | 116.0 | 76.1 | 65.5 | 15.9 | 10.9 | 13.7 |
| SCC89 | JX1/8 × JX1/119 | 82.8 | 0.0 | 44.9 | 238.2 | 7.9 | 127.0 | 83.7 | 66.1 | 15.4 | 13.6 | 11.4 |
| SCC90 | JX1/8 × JX1/23 | 57.1 | 12.5 | 32.5 | 172.6 | 5.7 | 92.1 | 62.0 | 67.4 | 12.1 | 14.1 | 14.0 |
| SCS95 | JX1/8 × JX1/8 | 36.7 | 0.0 | 20.8 | 110.5 | 3.6 | 58.9 | 38.5 | 67.6 | 14.7 | 6.5 | 14.9 |
| SCS90 | JX1/80 × JX1/80 | 15.0 | 11.1 | 7.8 | 41.4 | 2.3 | 22.1 | 16.7 | 76.1 | 14.7 | 7.7 | 16.1 |
| SCS91 | JX1/81 × JX1/81 | 21.5 | 16.7 | 13.0 | 69.1 | 2.3 | 36.8 | 25.8 | 70.7 | 13.5 | 9.5 | 15.6 |
| SCS92 | JX1/82 × JX1/82 | 39.9 | 0.0 | 22.1 | 117.4 | 3.9 | 62.6 | 40.3 | 64.5 | 9.4 | 7.6 | 16.0 |
| SCC83 | JX1/83 × JX1/33 | 72.4 | 0.0 | 40.3 | 214.1 | 7.1 | 114.2 | 74.5 | 65.4 | 16.1 | 12.8 | 15.5 |
| SCS93 | JX1/85 × JX1/85 | 16.0 | 22.2 | 9.1 | 48.3 | 3.1 | 25.8 | 16.4 | 62.5 | 13.8 | 7.8 | 14.7 |
| SCS94 | JX1/86 × JX1/86 | 24.8 | 0.0 | 14.3 | 76.0 | 2.5 | 40.5 | 26.8 | 69.0 | 16.6 | 7.5 | 14.3 |
| SCC84 | JX1/87 × JX1/118 | 0.0 | 0.0 | - | - | - | - | - | - | - | - | - |
| SCC85 | JX1/87 × JX1/6 | 65.4 | 16.7 | 32.5 | 172.6 | 5.7 | 92.1 | 62.7 | 68.3 | 17.2 | 12.9 | 12.5 |
| SCC86 | JX1/89 × JX1/63 | 30.3 | 36.7 | 16.9 | 89.8 | 3.0 | 47.9 | 31.0 | 65.2 | 16.0 | 14.5 | 15.1 |
| SCC91 | JX1/90 × JX1/51 | 67.9 | 0.0 | 40.3 | 214.1 | 7.1 | 114.2 | 72.1 | 63.3 | 15.0 | 13.9 | 14.2 |
| SCS96 | JX1/90 × JX1/90 | 8.3 | 25.0 | 3.9 | 20.7 | 0.7 | 11.1 | 9.4 | 42.6 | 8.2 | 4.1 | 6.2 |
| SCC92 | JX1/98 × JX1/51 | 33.9 | 13.3 | 19.5 | 103.6 | 3.4 | 55.2 | 38.2 | 69.7 | 15.5 | 12.3 | 15.3 |
| SCC93 | JX1/J 1 × JX1/33 | 53.5 | 9.5 | 29.9 | 158.8 | 5.2 | 84.7 | 53.3 | 63.0 | 13.7 | 11.9 | 17.1 |
| SCC95 | JX1/J1 × JX1/ 59 | 60.7 | 16.7 | 36.4 | 193.4 | 6.4 | 103.1 | 66.2 | 63.6 | 14.4 | 14.1 | 16.4 |
| SCC96 | JX1/J1 × JX1/ 90 | 21.9 | 8.3 | 11.7 | 62.2 | 2.0 | 33.1 | 24.0 | 71.0 | 14.3 | 12.4 | 15.6 |
| SCC97 | JX1/J1 × JX1/23 | 93.2 | 0.0 | 53.3 | 283.1 | 9.3 | 151 | 94.4 | 62.5 | 16.7 | 14.6 | 13.6 |
| SCC99 | JX1/J1 × JX1/51 | 76.2 | 0.0 | 42.9 | 227.9 | 7.5 | 121.5 | 80.4 | 66.1 | 16.7 | 13.4 | 16.0 |
| SCC100 | JX1/J1 × JX1/66 | 2.4 | 0.0 | 1.3 | 6.9 | 0.2 | 3.7 | 2.9 | 26.0 | 3.2 | 17.9 | 4.1 |
| SCC94 | JX1/J1 × JX1/6 | 50.0 | 0.0 | 22.1 | 117.4 | 3.9 | 62.6 | 39.6 | 59.0 | 17.4 | 9.6 | 16.0 |
| SCC101 | JX1/JX1 × JX1/20 | 59.4 | 0.0 | 39.0 | 207.2 | 6.8 | 110.5 | 66.5 | 60.9 | 15.0 | 12.0 | 13.7 |
|  | S.e  Mean | 12.3  47.1 | 17.4  7.67 | 5.7  25.8 | 30.2  137.2 | 1.0  4.6 | 16.1  73.18 | 11.8  47.4 | 13.7  59.0 | 4.2  13.1 | 4.3  9.0 | 3.8  13.1 |

PS = Pod set (%), PSP = Pseudo pod set (%), NP= Number of pod. PW = Pod weight, NNP = Number of nuts/pod, WUN (g) = Weight of unpeeled nuts (g), OT = Outturn (%), PA = potential alcohol, FN = firmness of nuts (lb), SCC = Single hybrid cross, SCS = Single hybrid self-cross

**Supplementary Table S3.** Cluster characteristics of the Bunso progeny crosses.

| Trait | v.test | Mean in category | Overall mean | Sd in category | Overall Sd | p.value |
| --- | --- | --- | --- | --- | --- | --- |
| ***Cluster 1*** |  |  |  |  |  |  |
| NN | -5.1 | 4.3 | 5.6 | 0.8 | 1.5 | 2.5E-09 |
| NW | -6.4 | 5.5 | 7.6 | 2.2 | 2.1 | 1.2E-10 |
| OT | -7.1 | 66.8 | 79.3 | 10.5 | 11.9 | 1.1E-12 |
| WUN | -7.5 | 65.4 | 116.9 | 13.6 | 46.6 | 5.1E-14 |
| WPN | -7.5 | 47.6 | 99.0 | 13.6 | 46.6 | 5.1E-14 |
| NP | -7.5 | 22.9 | 41.0 | 4.8 | 16.4 | 5.1E-14 |
| PW | -7.5 | 100.1 | 178.8 | 20.7 | 71.2 | 5.1E-14 |
| NL | -8.0 | 6.1 | 8.5 | 0.9 | 2.0 | 1.3E-15 |
| PS | -8.5 | 35.5 | 63.4 | 7.3 | 22.5 | 2.9E-17 |
| ***Cluster 2*** |  |  |  |  |  |  |
| NL | 4.6 | 9.8 | 8.5 | 0.8 | 2.0 | 3.1E-06 |
| PS | 4.2 | 76.4 | 63.4 | 8.5 | 22.5 | 2.3E-05 |
| PWD | 4.1 | 8.7 | 7.5 | 0.9 | 2.1 | 3.8E-05 |
| OT | 3.1 | 84.2 | 79.3 | 4.9 | 11.9 | 0.0 |
| PWD | 2.3 | 13.2 | 12.8 | 0.9 | 1.0 | 0.0 |
| ***Cluster 3*** |  |  |  |  |  |  |
| NNP | 7.1 | 7.7 | 5.6 | 0.9 | 1.5 | 1.3E-12 |
| WUN | 6.7 | 178.1 | 116.9 | 22.4 | 46.6 | 1.9E-11 |
| WPN | 6.7 | 160.3 | 99.0 | 22.4 | 46.6 | 1.9E-11 |
| PW | 6.7 | 272.6 | 178.8 | 34.3 | 71.2 | 1.9E-11 |
| NP | 6.7 | 62.6 | 41.0 | 7.9 | 16.4 | 1.9E-11 |
| PS | 4.7 | 83.9 | 63.4 | 6.1 | 22.5 | 3.4E-06 |
| OT | 4.5 | 89.7 | 79.3 | 1.2 | 11.9 | 7.5E-06 |
| NL | 3.6 | 9.9 | 8.5 | 1.1 | 2.0 | 0.0 |
| NW | 2.5 | 8.5 | 7.5 | 0.9 | 2.1 | 0.0 |

Sd: standard deviation, PS = Pod set, PSP =Pseudo-pod set, NP = number of pods, PW = Pod weight, PL = Pod length, PWD =Pod width, NNP = Number of nuts per pod, NL = Nut length, NW = Nut width, WUN = Weight of unpeeled nuts, WPN = Weight of peeled nuts, OT = Outturn

**Supplementary Table S4**. Cluster characteristics of JX1 crosses.

| Traits | v.test | Mean in category | Overall mean | Sd in category | Overall Sd | p.value |
| --- | --- | --- | --- | --- | --- | --- |
| ***Cluster 1*** |  |  |  |  |  |  |
| PS | -6.3 | 2.7 | 46.8 | 3.7 | 27.7 | 4.13E-10 |
| PW | -6.3 | 7.9 | 136.5 | 11.3 | 80.3 | 3.25E-10 |
| NP | -6.3 | 1.5 | 25.7 | 2.1 | 15.1 | 3.25E-10 |
| WUN | -6.3 | 4.2 | 72.8 | 6.0 | 42.8 | 3.25E-10 |
| WPN | -6.5 | 2.8 | 47.1 | 4.0 | 26.8 | 9.18E-11 |
| NNP | -6.6 | 0.3 | 4.6 | 0.4 | 2.6 | 5.12E-11 |
| PA | -7.5 | 2.7 | 9.0 | 4.6 | 3.3 | 6.48E-14 |
| NW | -7.9 | 0.9 | 5.2 | 1.0 | 2.1 | 4.20E-15 |
| NL | -8.9 | 1.0 | 5.8 | 1.1 | 2.1 | 8.73E-19 |
| PWD | -9.1 | 2.9 | 11.8 | 2.4 | 3.8 | 1.43E-19 |
| Brix | -9.9 | 2.3 | 13.1 | 2.4 | 4.3 | 3.39E-23 |
| FN.Ib | -10.1 | 3.4 | 13.2 | 3.4 | 3.8 | 3.91E-24 |
| PL | -10.8 | 2.9 | 14.3 | 2.6 | 4.1 | 4.13E-27 |
| OT | -10.9 | 11.6 | 59.2 | 11.7 | 17.0 | 5.33E-28 |
| ***Cluster 2*** |  |  |  |  |  |  |
| PSP | 5.1 | 14.2 | 7.6 | 13.3 | 11.2 | 2.75E-07 |
| OT | 2.9 | 64.7 | 59.2 | 8.6 | 17.0 | 0.004285 |
| PL | 2.7 | 15.5 | 14.3 | 1.9 | 4.1 | 0.007979 |
| FN.Ib | 1.9 | 14.1 | 13.2 | 1.9 | 3.8 | 0.047538 |
| NL | -2.7 | 5.1 | 5.8 | 1.2 | 2.1 | 0.006358 |
| NW | -2.9 | 4.5 | 5.2 | 0.9 | 2.1 | 0.003788 |
| NNP | -6.9 | 2.5 | 4.6 | 0.9 | 2.6 | 5.38E-12 |
| WPN | -7.0 | 25.6 | 47.1 | 10.3 | 26.8 | 2.51E-12 |
| WUN | -7.2 | 37.7 | 72.8 | 16.1 | 42.8 | 7.74E-13 |
| PW | -7.2 | 70.6 | 136.5 | 30.2 | 80.3 | 7.74E-13 |
| NP | -7.2 | 13.3 | 25.7 | 5.7 | 15.1 | 7.74E-13 |
| PS | -7.3 | 23.6 | 46.8 | 9.9 | 27.7 | 2.86E-13 |
| ***Cluster 3*** |  |  |  |  |  |  |
| PS | 10.7 | 69.3 | 46.8 | 11.7 | 27.7 | 6.88E-27 |
| PW | 10.6 | 201.0 | 136.5 | 35.3 | 80.3 | 2.18E-26 |
| NP | 10.6 | 37.8 | 25.7 | 6.6 | 15.1 | 2.18E-26 |
| WUN | 10.6 | 107.2 | 72.8 | 18.8 | 42.8 | 2.18E-26 |
| WPN | 10.6 | 68.6 | 47.1 | 11.5 | 26.8 | 3.36E-26 |
| NNP | 10.5 | 6.6 | 4.6 | 1.2 | 2.6 | 5.79E-26 |
| NL | 7.9 | 7.1 | 5.8 | 1.1 | 2.1 | 2.65E-15 |
| NW | 7.5 | 6.4 | 5.2 | 1.5 | 2.1 | 8.17E-14 |
| PWD | 7.2 | 13.9 | 11.8 | 1.8 | 3.8 | 5.18E-13 |
| PA | 6.1 | 10.6 | 9.0 | 1.9 | 3.3 | 9.93E-10 |
| Brix | 6.0 | 14.9 | 13.1 | 1.9 | 4.3 | 1.92E-09 |
| FN.Ib | 4.2 | 14.4 | 13.2 | 1.7 | 3.8 | 3.23E-05 |
| PL | 3.9 | 15.5 | 14.3 | 1.4 | 4.1 | 9.60E-05 |
| OT | 3.8 | 64.1 | 59.2 | 2.9 | 17.0 | 0.000135 |
| PSP | -3.8 | 4.4 | 7.6 | 7.9 | 11.2 | 0.000128 |

Sd = Standard deviation, PS = Pod set, PSP =Pseudo-pod set, NP = number of pods, PW = Pod weight (g), PL = Pod length (cm), PWD =Pod width (cm), NNP = Number of nuts per pod, NL = Nut length (cm), NW = Nut width (cm), WUN = Weight of unpeeled nuts (g), WPN = Weight of peeled nuts (g), OT = Outturn (%), PA= Potential alcohol, FN = Firmness of nuts (Ib)

**Supplementary Table S5.** Cluster characteristics of GX1 crosses.

| Traits | v.test | Mean in category | Overall mean | Sd in category | Overall Sd | p.value |
| --- | --- | --- | --- | --- | --- | --- |
| ***Cluster 1*** |  |  |  |  |  |  |
| PW | -4.0 | 39.2 | 131.3 | 16.3 | 68.9 | 5.57E-05 |
| NP | -4.1 | 3.7 | 12.4 | 1.5 | 6.5 | 5.04E-05 |
| WUN | -4.1 | 22.8 | 74.3 | 10.2 | 38.1 | 4.79E-05 |
| NNP | -4.1 | 2.3 | 7.4 | 0.9 | 3.8 | 4.05E-05 |
| PS | -4.3 | 8.7 | 29.5 | 4.2 | 14.8 | 2.09E-05 |
| WPN | -4.5 | 15.8 | 52.2 | 6.2 | 24.5 | 7.30E-06 |
| ***Cluster 2*** |  |  |  |  |  |  |
|  |  |  |  |  |  |  |
| ***Cluster 3*** |  |  |  |  |  |  |
| WUN | 4.0 | 120.6 | 74.3 | 13.4 | 38.1 | 5.89E-05 |
| NNP | 4.0 | 11.9 | 7.4 | 1.3 | 3.8 | 6.04E-05 |
| NP | 3.9 | 20.1 | 12.4 | 2.6 | 6.5 | 9.83E-05 |
| PW | 3.9 | 211.8 | 131.3 | 27.2 | 68.9 | 0.000111 |
| PS | 3.9 | 46.8 | 29.5 | 5.0 | 14.8 | 0.000113 |
| WPN | 3.5 | 77.9 | 52.2 | 3.2 | 24.5 | 0.000518 |

Sd = Standard deviation, PS = Pod set (%), NP = Number of pods, PW = Pod weight (g), NNP = Number of nuts per pod, WUN = Weight of unpeeled nuts (g), WPN = Weight of peeled nuts (g)

**Supplementary Table S6**. Cluster characteristics of MX2 crosses.

| Traits | v.test | Mean in category | Overall mean | Sd in category | Overall Sd | p.value |
| --- | --- | --- | --- | --- | --- | --- |
| ***Cluster 1*** |  |  |  |  |  |  |
| NL | 3.9 | 5.8 | 4.4 | 0.4 | 0.5 | 6.87E-05 |
| NW | 3.5 | 4.7 | 3.7 | 0.2 | 0.4 | 0.000393 |
| ***Cluster 2*** |  |  |  |  |  |  |
| WUN | -3.5 | 41.9 | 51.9 | 3.2 | 8.7 | 0.000533 |
| OT | -3.5 | 73.3 | 77.5 | 2.1 | 3.6 | 0.000519 |
| WPN | -3.6 | 31.1 | 40.7 | 2.5 | 8.1 | 0.000356 |
| NNP | -3.6 | 4.6 | 5.7 | 0.3 | 0.9 | 0.000355 |
| ***Cluster 3*** |  |  |  |  |  |  |
| Brix | 2.3 | 15.8 | 12.6 | 3.0 | 3.8 | 0.019338 |
| PWD | 2.2 | 7.9 | 7.3 | 0.5 | 0.7 | 0.030494 |
| ***Cluster 4*** |  |  |  |  |  |  |
| NP | 3.3 | 24.4 | 18.3 | 3.5 | 5.0 | 0.001044 |
| PS | 3.3 | 40.1 | 31.1 | 7.0 | 7.5 | 0.001055 |
| PW | 3.3 | 239.5 | 179.4 | 34.9 | 50.4 | 0.001124 |
| PA | -2.3 | 6.0 | 7.2 | 1.1 | 1.4 | 0.024634 |
| ***Cluster 5*** |  |  |  |  |  |  |
| NNP | 3.3 | 6.7 | 5.7 | 0.4 | 0.9 | 0.000995 |
| WUN | 3.2 | 62.0 | 51.9 | 3.0 | 8.7 | 0.001466 |
| WPN | 3.1 | 49.8 | 40.7 | 2.8 | 8.1 | 0.002072 |
| OT | 2.1 | 80.2 | 77.5 | 2.0 | 3.6 | 0.034571 |
| PA | 2.0 | 8.2 | 7.2 | 1.1 | 1.4 | 0.044678 |
| Brix | -2.3 | 9.4 | 12.6 | 2.1 | 3.8 | 0.020314 |

Sd = Standard deviation, PS = Pod set (%), PSP =Pseudo pod set (%), NP = number of pods, PW = Pod weight (g), PL = Pod length (cm), PWD =Pod width (cm), NNP = Number of nuts per pod, NL = Nut length (cm), NW = Nut width (cm), WUN = Weight of unpeeled nuts (g), WPN = Weight of peeled nuts (g), OT = Outturn (%), PA= Potential alcohol, FN = Firmness of nuts (Ib)

**Supplementary Table S7.** Mid-parent, better parent and economic heterosis for pod set, outturn and brix among the BUNSO progeny crosses.

|  | MPH(%) | | | BPH(%) | | | ECH1(%) | | | ECH2(%) | | |
| --- | --- | --- | --- | --- | --- | --- | --- | --- | --- | --- | --- | --- |
| Crosses | Pod set (%) | Outturn (%) | Brix | Pod set (%) | Outturn (%) | Brix | Pod set (%) | Outturn (%) | Brix | Pod set (%) | Outturn (%) | Brix |
| B1/11 x B1/71 x B1/151xB1/180 | 184.2 | 29.1 | -3.7 | 158.9 | 19.8 | -10.5 | 77.6 | 19.5 | 5.1 | 71.9 | 24.8 | 16.9 |
| B1/11xB1/71 x B1/157 x B1/149 | 206.3 | 42.4 | 3.6 | 199.6 | 24.2 | 2.9 | 105.4 | 23.9 | 3.6 | 98.9 | 29.4 | 15.3 |
| B1/11 xB1/71 x B1/296 x B1/177 | 167.4 | 18.7 | 5.1 | 146.8 | 14.3 | 2.8 | 69.3 | 22.9 | 5.1 | 63.8 | 28.4 | 16.4 |
| B1/11 x B1/71 x B2/177 x B2/156 | 153.9 | 23.8 | 4.7 | 134.6 | 10.3 | 11.2 | 37.8 | 10.1 | 3.6 | 55.7 | 14.9 | 15.3 |
| B1/11 XB1/71 xGX1/46 xGX1/16 | 119.9 | 25.4 | -4.7 | 85.3 | 25.2 | -5.0 | -31.4 | 25.2 | -4.3 | 33.6 | 30.8 | 6.5 |
| B1/11 x B1/71 x GX1/46 x GX1/53 | 86.2 | 36.0 | -2.7 | 47.7 | 24.9 | -8.3 | 72.9 | 24.6 | -80.2 | 67.4 | 30.1 | 79.4 |
| B1/120 x B1/193 x GX1/46 x GX1/53 | 70.6 | 17.2 | -2.1 | 45.9 | 14.7 | -2.8 | 70.9 | 23.3 | -80.8 | 65.4 | 28.8 | -79.9 |
| B1/120 x B1/193 x JX1/9 x JX1/11 | 109.3 | -9.4 | 3.7 | 103.9 | -9.9 | 0.0 | 79.1 | -7.5 | -78.7 | 73.4 | -3.4 | -77.7 |
| B1/151 x B1/147 x GX1/46 x GX1/53 | 96.1 | 23.2 | 16.9 | 54.3 | 14.4 | 11.6 | 80.7 | 23.2 | -76.3 | 74.9 | 28.6 | -75.2 |
| B1/151 x B1/149 x B1/11 x B1/71 | 176.2 | 30.8 | -13.5 | 155.0 | 19.8 | -18.5 | 74.9 | 19.5 | -83.5 | 69.3 | 24.7 | -81.7 |
| B1/151 x B1/149 x B2/177 x B2/156 | 109.6 | 16.2 | 10.9 | 109.5 | 9.1 | 3.8 | 21.7 | -8.5 | -77.7 | 17.9 | -4.4 | -76.6 |
| B1/208 x B1/180 x JX1/24 x JX1/22 | 101.0 | 19.7 | 3.9 | 97.8 | 19.7 | -4.2 | 53.1 | 19.9 | -77.9 | 48.2 | 25.2 | -76.9 |
| B1/211 x B1/200 x B1/157 x B1/149 | 159.3 | 26.8 | -11.3 | 157.5 | 16.7 | -15.3 | 68.9 | 14.9 | -81.8 | 63.5 | 20.1 | -80.9 |
| B1/212 x B1/236 x JX1/24 x JX1/22 | 176.9 | 26.1 | 1.9 | 116.8 | 8.9 | -4.3 | 67.8 | 17.1 | -78.9 | 62.5 | 22.3 | -77.9 |
| B1/296 x B1/177 x GX1/46 x GX1/53 | 103.6 | 19.6 | -8.5 | 52.2 | 17.3 | -16.7 | 78.1 | 17.4 | -80.8 | 72.5 | 22.6 | -79.9 |
| B2/177 x B2/156 x B1/151 x B1/147 | 45.2 | 26.1 | 1.9 | 35.3 | 8.9 | -4.3 | -9.4 | 17.1 | -78.9 | -11.9 | 22.3 | -77.9 |
| B2/177 x B2/156 x JX1/9 x JX1/11 | 128.6 | 15.9 | -1.4 | 89.9 | 4.6 | -7.1 | 66.7 | -3.3 | -80.2 | 61.4 | 1.0 | -79.4 |
| B1/212 x B1/210 x GX1/46 x GX1/53 | 89.2 | 38.0 | 8.1 | 42.1 | 19.5 | 1.9 | 66.4 | 22.6 | -78.3 | 61.1 | 28.1 | -77.4 |
| Club x JB 32 x JX1/5 x JX1/9 | 242.5 | 54.3 | -5.6 | 199.2 | 32.5 | -16.6 | 99.8 | 26.9 | -81.3 | 93.4 | 32.5 | -80.5 |
| GX1/46 x GX1/16 x B1/151 x B1/180 | 42.7 | 8.7 | 8.1 | 11.6 | -1.9 | 6.8 | 11.6 | 4.1 | -76.2 | 8.0 | 8.7 | -75.2 |
| GX1/46 x GX1/16 x GX1/46 x GX1/53 | 30.5 | 14.1 | 22.6 | 21.0 | 10.2 | 19.9 | 41.7 | 18.5 | -76.5 | 37.2 | 23.9 | -75.5 |
| GX1/46 x GX1/16 x JX1/17 x JX1/9 | 89.7 | 13.0 | -5.8 | 77.4 | 9.8 | -7.6 | 77.4 | 16.6 | -79.8 | 71.8 | 21.8 | -79.0 |
| GX1/46 x GX1/16 x JX1/24 x JX1/22 | 87.4 | 18.9 | -4.0 | 66.2 | 15.5 | -9.5 | 66.2 | 22.4 | -80.4 | 60.9 | 28.1 | -79.5 |
| GX1/46 x GX1/16 x JX1/5 x JX1/9 | 120.4 | 25.3 | 9.2 | 83.8 | 22.6 | 3.6 | 83.8 | 22.6 | -80.4 | 77.9 | 28.1 | -79.5 |
| GX1/46 x GX1/16 x JX1/9 x GX1/16 | 73.5 | 14.9 | -1.8 | 65.9 | 12.2 | -3.5 | 65.9 | 19.1 | -80.9 | 60.6 | 24.4 | -80.1 |
| GX1/46 x GX1/16 x JX1/9 x JX1/11 | 91.8 | 23.1 | -2.0 | 80.1 | 21.7 | -7.1 | 80.1 | 24.8 | -80.2 | 74.4 | 30.4 | -79.4 |
| GX1/46 x GX1/33 x B1/212x B1/236 | 168.3 | 70.2 | 8.6 | 107.9 | 18.8 | 4.8 | 65.8 | 18.1 | -79.1 | 60.5 | 23.4 | -78.2 |
| GX1/46 x GX1/33 x JX1/24 x JX1/22 | 130.4 | 23.1 | 6.4 | 127.1 | 22.8 | -0.6 | 81.1 | 22.9 | -77.1 | 75.3 | 28.4 | -76.1 |
| GX1/46 x GX1/53 x B2/296 x B1/177 | - | − | − | - | − | − | 81.1 | − | − | 75.3 | − | − |
| GX1/46 x GX1/53 x GX1/46 x GX1/16 | 26.0 | 15.4 | 7.5 | 16.8 | 10.8 | 6.4 | 36.8 | 19.2 | -79.4 | 32.5 | 24.5 | -78.5 |
| GX1/46 x GX1/53 x JX1/17 x JX1/5 | 82.8 | 12.9 | 9.2 | 57.1 | 10.7 | 8.4 | 83.9 | 19.1 | -78.8 | 78.2 | 24.4 | -77.8 |
| GX1/46 x GX1/53 x JX1/24 x JX1/22 | 44.7 | 11.6 | -0.1 | 20.2 | 7.7 | -0.2 | 40.8 | 15.9 | -80.7 | 36.3 | 21.1 | -79.8 |
| GX1/46 x GX1/53 x JX1/9 x JX1/11 | 55.2 | 2.0 | 9.5 | 35.8 | 2.0 | 4.5 | 58.9 | 9.7 | -77.8 | 53.9 | 14.6 | -76.8 |
| JX1/14 x JX1/32 x JX1/9 x JX1/11 | 64.8 | 17.1 | -6.1 | 55.4 | 16.9 | -6.4 | 36.5 | 20.3 | -79.9 | 32.1 | 25.6 | -79.1 |
| JX1/17 x JX1/5 x JX1/24 x JX1/22 | 97.1 | 17.4 | -5.5 | 89.1 | 15.5 | -12.5 | 59.3 | 19.5 | -79.8 | 54.2 | 24.8 | -78.9 |
| JX1/17 x JX1/5 x JX1/9 x JX1/11 | 103.2 | 19.4 | 4.7 | 98.9 | 18.9 | 0.6 | 74.8 | 23.0 | -78.6 | 69.2 | 28.5 | -77.7 |
| JX1/17 x JX1/9 x B1/212 x B1/210 | 48.4 | 0.3 | -12.2 | 24.3 | -9.0 | -12.7 | 8.1 | -8.9 | -79.3 | 4.7 | -4.9 | -78.4 |
| JX1/17 x JX1/9 x GX1/46 x GX1/16 | 61.0 | 18.7 | 1.6 | 50.5 | 18.6 | -7.7 | 50.5 | 18.8 | -78.6 | 45.7 | 24.1 | -77.7 |
| JX1/23 x JX1/53 x GX1/46 x GX1/16 | 76.3 | 14.9 | 10.1 | 64.9 | 13.5 | 9.03 | 64.9 | 16.5 | -76.8 | 59.7 | 21.6 | -75.8 |
| JX1/23 x JX1/53 x GX1/46 x GX1/53 | 72.6 | 17.1 | 1.9 | 50.6 | 16.9 | 1.8 | 76.3 | 17.1 | -78.6 | 70.7 | 22.3 | -77.7 |
| JX1/24 x JX1/22 x B1/151 x B1/147 | 104.1 | 17.5 | -12.5 | 90.7 | 16.0 | -16.8 | 47.2 | 19.1 | -82.3 | 42.6 | 24.4 | -81.5 |
| JX1/24 x JX1/22 x GX1/46 x GX1/16 | 79.3 | 17.1 | 1.9 | 59.0 | 16.9 | 1.8 | 59.0 | 17.1 | -78.6 | 53.9 | 22.3 | -77.7 |
| JX1/24 x JX1/22 x GX1/46 x GX1/53 | 68.2 | 15.2 | -1.6 | 39.7 | 11.2 | -9.5 | 63.6 | 19.1 | -79.1 | 58.4 | 24.9 | -78.2 |
| JX1/24 x JX1/22 x JX1/7 x JX1/53 | 83.1 | 12.8 | -16.5 | 73.2 | 9.6 | -78.6 | 50.4 | 16.3 | -80.2 | 45.6 | 21.5 | -79.4 |
| JX1/5 x JX1/9 x GX1/46 x GX1/16 | 68.3 | 13.5 | 14.5 | 40.4 | 11.1 | 8.7 | 40.4 | 11.1 | -79.4 | 35.9 | 16.0 | -78.5 |
| JX1/5 x JX1/9 x JX1/9 x JX1/11 | 120.5 | 22.2 | 13.9 | 93.8 | 18.4 | 2.6 | 70.2 | 21.5 | -78.2 | 64.8 | 27.7 | -77.2 |
| JX1/7 x JX1/53 x JX1/7 x JX1/5 | 122.5 | 22.1 | -9.4 | 103.6 | 13.7 | -75.3 | 76.8 | 20.7 | -79.4 | 71.2 | 26.1 | -78.5 |
| JX1/9 x JX1/11 x GX1/46 x GX1/53 | 80.9 | 13.9 | 4.8 | 58.4 | 11.4 | 0.0 | 85.5 | 19.8 | -78.7 | 79.6 | 25.1 | -77.7 |
| JX1/9 x JX1/11 x JX1/17 x JX1/5 | 88.5 | 10.7 | 24.2 | 84.6 | 10.2 | 19.4 | 62.2 | 13.9 | -74.6 | 57.1 | 19.1 | -73.5 |
| JX1/9 x JX1/11 x JX1/24 x JX1/22 | 96.9 | 14.9 | -2.7 | 85.2 | 13.5 | 13.7 | 62.7 | 16.5 | -80.1 | 57.6 | 21.6 | -79.2 |
| JX1/9 x JX1/11 x JX1/5 x JX1/9 | 108.3 | 17.3 | 9.7 | 83.4 | 13.5 | -1.3 | 61.1 | 16.5 | -79.0 | 55.9 | 21.6 | -78.1 |
| JX1/9 x JX1/11 x JX1/7 x JX1/5 | 67.4 | 14.6 | -12.6 | 52.4 | 8.0 | -12.9 | 33.9 | 10.8 | -81.5 | 29.6 | 15.8 | -80.7 |
| JX1/9 x JX1/11 x JX1/7 x JX1/53 | 94.7 | 13.9 | -16.3 | 93.6 | 12.1 | -21.5 | 70.0 | 19.1 | -80.9 | 64.6 | 24.4 | -80.1 |

MPH = Mid parent heterosis, BPH = Better parent heterosis, ECH1 = Economic heterosis in relation to standard variety 1 (GX1/46 × GX1/16), Economic heterosis in relation to standard variety 2 (JX1/5 × JX1/9).

**Supplementary Table S8**. Mid parent, better parent and economic heterosis for pod set, outturn and brix among the JX1 crosses.

|  | MPH (%) | | | BPH (%) | | | | ECH1 (%) | | | ECH2 (%) | | |
| --- | --- | --- | --- | --- | --- | --- | --- | --- | --- | --- | --- | --- | --- |
| Cross | %Pod set | Outturn% | Brix | %Pod set | Outturn% | Brix | | Pod set% | Outturn% | Brix | Pod set% | Outturn% | Brix |
| JX1/1 x JX1/112 | - | - | - | - | - | | - | 47.1 | -9.2 | 6.5 | 42.5 | 5.1 | 18.6 |
| JX1/1 x JX1/67 | - | - | - | - | - | | - | 66.8 | -1.3 | -7.2 | 61.5 | 3.1 | 0.1 |
| JX1/10 x JX1/36 | - | -6.5 | -0.1 | - | -10.2 | | -2.6 | - | -9.8 | -10.4 | - | -5.8 | 1.9 |
| JX1/10 x JX1/48 | - | 68.3 | 101.1 | - | -15.9 | | 0.6 | 56.4 | -15.5 | -10.4 | 33.9 | -11.7 | -0.2 |
| JX1/108 x JX1/23 | 207.1 | 94.6 | 142.8 | 53.6 | -2.7 | | 21.4 | 40.4 | -15.4 | -2.2 | 35.9 | -11.7 | 8.9 |
| JX1/108 x JX1/6 | 171.9 | 17.7 | 28.9 | 69.2 | 0.9 | | 23.7 | 54.6 | -12.2 | 8.7 | 49.7 | -8.3 | 20.9 |
| JX1/11 x JX1/23 | - | - | - | - | - | | - | 30.8 | 3.1 | 6.5 | 26.7 | 7.7 | 4.0 |
| JX1/112 x JX1/23 | - | - | - | - | - | | - | -93.1 | -65.9 | 65.4 | -93.2 | -58.7 | -61.5 |
| JX1/117 x JX1/73 | - | 121.5 | 146.5 | - | 10.7 | | 23.3 | 8.2 | -5.5 | 15.2 | 4.7 | -1.3 | 28.2 |
| JX1/118 x JX1/23 | 434.6 | 76.8 | 77.2 | 167.3 | -11.6 | | -11.3 | 45.4 | -17.0 | -9.2 | 40.8 | -13.3 | 1.1 |
| JX1/118 x JX1/36 | -21.3 | -9.9 | 10.7 | -38.7 | -10.6 | | 20.3 | -66.7 | -16.0 | 23.2 | -67.7 | -12.3 | 37.1 |
| JX1/118 x JX1/6 | 260.0 | 10.2 | 7.6 | 153.8 | -8.4 | | 0 | 38.0 | -14.0 | 2.4 | 33.7 | -10.2 | 13.9 |
| JX1/118 x JX1/8 | -60.6 | -41.2 | -15.3 | -66.1 | -41.5 | | -16.9 | -74.4 | -45.1 | -11.4 | -75.2 | -42.7 | -1.4 |
| JX1/119 x JX1/23 | 499.2 | 76.6 | 141.6 | 199.6 | -11.7 | | 20.9 | 24.3 | -11.5 | -6.5 | 20.4 | -7.6 | 4.0 |
| JX1/119 x JX1/32 | 349.3 | -15.9 | 57.2 | 273.4 | -16.5 | | 54.6 | 54.9 | -15.2 | 23.7 | 50.0 | -11.5 | 37.7 |
| JX1/119 xJX1/50 | 308.6 | -10.5 | 57.2 | 268.6 | -15.9 | | 54.6 | 52.9 | -11.7 | 23.7 | 48.1 | -12.1 | 37.7 |
| JX1/2 x JX1/25 | - | -36.1 | -5.8 | - | -68.0 | | -52.9 | -72.5 | -69.2 | -60.2 | -73.4 | -67.9 | -55.7 |
| JX1/2 x JX1/27 | 113.4 | -0.8 | -15.8 | 43.9 | -7.4 | | -24.9 | 35.2 | -10.8 | -19.1 | 30.9 | -6.9 | -9.9 |
| JX1/2 x JX1/45 | 164.1 | -8.7 | 20.4 | 76.7 | -9.4 | | 10.5 | 65.9 | -12.8 | 11.8 | 60.6 | -8.9 | 24.4 |
| JX1/2 x JX1/54 | - | 74.4 | 124.3 | - | -12.7 | | 12.3 | 35.4 | -16.0 | -5.1 | 31.1 | -12.3 | 5.7 |
| JX1/2 x JX1/6 | 161.3 | 6.9 | 27.3 | 61.7 | -12.0 | | 24.7 | 51.7 | -15.3 | 9.6 | 46.9 | -11.5 | 22.0 |
| JX1/2 x JX1/7 | 74.5 | -11.8 | 7.7 | 56.1 | -14.9 | | 1.2 | 46.5 | -18.1 | -2.7 | 41.9 | -14.4 | 8.3 |
| JX1/20 x JX1/118 | 203.3 | -3.2 | 39.1 | 178.5 | -6.4 | | 14.2 | 81.1 | -12.1 | 16.9 | 75.3 | -8.2 | 30.1 |
| JX1/20 x JX1/119 | 149.1 | -7.2 | 55.3 | 104.1 | -12.9 | | 43.7 | 32.7 | -12.7 | 11.1 | 28.5 | -8.9 | 23.6 |
| JX1/20 x JX1/31 | 78.2 | -3.4 | 34.8 | 34.1 | -4.3 | | 9.1 | 72.6 | -14.4 | 16.2 | 67.1 | -10.6 | 29.3 |
| JX1/20 x JX1/34 | 3.6 | 29.6 | 76.0 | -1.7 | 26.2 | | 67.7 | -36.1 | 16.9 | 20.3 | -38.1 | 22.1 | 33.9 |
| JX1/20 x JX1/50 | -30.8 | 4.9 | 7.4 | -47.6 | 4.8 | | -10.7 | -65.9 | -7.5 | -11.4 | -67.0 | -3.7 | -1.4 |
| JX1/20 xJX1/23 | 187.3 | 101.6 | 230.4 | 43.7 | 0.8 | | 65.4 | -6.6 | -11.5 | 8.7 | -9.6 | -7.6 | 20.9 |
| JX1/21 x JX1/9 | - | 102.3 | 103.9 | - | 1.4 | | 1.9 | 84.9 | -16.4 | 1.9 | 79.1 | -12.7 | 13.5 |
| JX1/22 x JX1/34 | 191.2 | -3.1 | 33.9 | 132.4 | -6.1 | | 13.5 | 35.5 | -7.4 | 15.7 | 31.2 | -3.3 | 28.8 |
| JX1/23 x JX1/34 | -49.5 | -39.9 | 11.9 | -74.8 | -69.9 | | -44.1 | -85.3 | -72.2 | -60.4 | -85.8 | -70.9 | -55.9 |
| JX1/24 x JX1/45 | 158.4 | -4.6 | 22.3 | 125.4 | 5.9 | | 20.6 | -5.2 | -8.4 | 25.6 | -8.2 | -4.4 | 39.8 |
| JX1/24 x JX1/6 | 351.3 | 16.1 | 2.9 | 246.7 | -4.8 | | -5.2 | 44.2 | -7.4 | -1.2 | 39.6 | -3.3 | 9.9 |
| JX1/25 x JX1/99 | - | - | - | - | - | | - | -61.7 | -8.6 | 20.8 | -62.9 | -4.5 | 34.4 |
| JX1/27 x JX1/25 | - | 114.2 | 127.2 | - | 7.1 | | 13.7 | - | -10.6 | 22.5 | - | -6.6 | 36.3 |
| JX1/27 x JX1/31 | 90.6 | 3.4 | -9.6 | 19.6 | -0.0 | | -10.1 | 53.9 | -10.6 | -3.1 | 49.1 | -6.7 | 7.8 |
| JX1/27 x JX1/89 | - | 99.6 | 72.9 | - | -0.2 | | -13.5 | 50.8 | -16.7 | -6.7 | 46.0 | -12.9 | 3.8 |
| JX1/30 x JX1/45 | 393.5 | -9.8 | 19.3 | 369.2 | -11.6 | | 17.4 | 49.1 | -12.6 | 18.8 | 44.3 | -8.7 | 32.3 |
| JX1/30 x JX1/51 | -43.6 | -42.7 | -53.8 | -71.8 | -71.4 | | -76.9 | -91.9 | -71.7 | -77.3 | -92.2 | -70.4 | -74.8 |
| JX1/30 x JX1/7 | 153.8 | -6.9 | 35.1 | 75.9 | -11.5 | | 33.8 | 30.3 | -12.4 | 31.2 | 26.1 | -8.5 | 45.9 |
| JX1/31 x JX1/23 | 134.0 | 109.8 | 139.9 | 17.0 | 4.9 | | 19.9 | 50.6 | -6.2 | 27.8 | 45.8 | -2.1 | 42.2 |
| JX1/31 x JX1/27 | 76.2 | 3.4 | -33.7 | 10.6 | 0.1 | | -34.1 | 42.3 | -10.6 | -28.9 | 37.8 | -6.6 | -20.9 |
| JX1/31 x JX1/28 | - | 92.8 | 118.6 | - | -3.6 | | 9.3 | 47.9 | -13.8 | 16.5 | 43.3 | -10.0 | 29.6 |
| JX1/31 x JX1/50 | - | -32.5 | -64.1 | - | -33.0 | | -65.3 | - | -40.1 | -63.0 | - | -37.5 | -58.9 |
| JX1/34 x JX1/23 | 14.9 | 101.5 | 190.4 | -42.5 | 0.8 | | 45.2 | -66.5 | -6.6 | 2.9 | -67.5 | -2.5 | 14.5 |
| JX1/34 x JX1/48 | - | 90.6 | 211.5 | - | -4.7 | | 55.7 | 22.7 | -11.7 | 10.4 | 18.8 | -7.8 | 22.8 |
| JX1/35 x JX1/23 | - | 0.0 | 0.0 | - | 0.0 | | 0.0 | 45.4 | -7.9 | -8.5 | 40.8 | 3.9 | 1.8 |
| JX1/45 x JX1/6 | 457.6 | 12.1 | -16.3 | 374.5 | -7.1 | | -16.2 | 50.7 | -12.0 | -15.2 | 45.9 | -8.1 | -5.7 |
| JX1/5 x JX1/9 | - | 0.0 | 0.0 | - | 0.0 | | 0.0 | - | -13.1 | 0.0 | - | -9.2 | 0.0 |
| JX1/50 x JX1/22 | -13.1 | -3.6 | -9.1 | -14.8 | -8.8 | | -10.2 | -70.4 | -10.1 | -8.5 | -71.4 | -6.1 | 1.9 |
| JX1/50 x JX1/31 | - | 2.5 | -11.8 | - | 1.7 | | -14.8 | - | -9.1 | -9.2 | - | -5.0 | 1.1 |
| JX1/51 x JX1/20 | 451.7 | 86.8 | 276.7 | 175.9 | -6.6 | | 88.5 | 79.4 | -18.0 | 23.9 | 73.7 | -14.4 | 37.9 |
| JX1/51 x JX1/25 | - | 0.0 | 0.0 | - | 0.00 | | 0.0 | 34.3 | -11.2 | 36.0 | 30.0 | -7.2 | 51.4 |
| JX1/51 x JX1/34 | 79.5 | 94.5 | 201.2 | -10.3 | -2.7 | | 50.6 | -47.7 | -9.9 | 6.7 | -49.3 | -5.9 | 18.8 |
| JX1/51 x JX1/36 | 947.4 | 85.8 | 171.7 | 423.7 | -7.1 | | 35.9 | 58.6 | -14.1 | 27.8 | 53.6 | -10.2 | 42.2 |
| JX1/51 x JX1/42 | 265.9 | 100.4 | 137.6 | 82.9 | 0.2 | | 18.9 | 58.5 | -16.7 | 24.1 | 53.4 | -12.9 | 38.2 |
| JX1/51 x JX1/ATTA 2 0.0 | | 0.0 | 0.0 | 0.0 | 0.0 | | 0.0 | 50.1 | -18.1 | 11.1 | 45.4 | -14.5 | 23.6 |
| JX1/51 xJX1/31 | 96.2 | 98.3 | 135.8 | 1.9 | -0.8 | | 17.9 | 26.2 | -11.4 | 25.6 | 22.2 | -7.4 | 39.8 |
| JX1/51/JX1/23 | 0.0 | 0.0 | 0.0 | 0.0 | 0.0 | | 0.0 | 87.0 | -12.5 | 3.6 | 81.0 | -8.7 | 15.3 |
| JX1/6 x JX1/113 | 0.0 | 169.3 | 144.3 | 0.0 | 34.7 | | 0.2 | 60.9 | -16.2 | 7.5 | 55.9 | -12.5 | 19.6 |
| JX1/6 x JX1/27 | -83.4 | -53.0 | -68.7 | -86.1 | -58.9 | | -71.6 | -95.4 | -65.8 | -69.4 | -95.6 | -64.2 | -65.9 |
| JX1/6 x JX1/54 | 0.0 | 174.43 | 173.9 | 0.0 | 37.2 | | 37.1 | 0.0 | -14.6 | 20.5 | 0.0 | -10.8 | 34.1 |
| JX1/6 x JX1/7 | 170.0 | 11.2 | 13.2 | 75.9 | -5.7 | | 8.3 | 30.2 | -15.7 | 4.1 | 26.1 | -11.9 | 15.9 |
| JX1/62 x JX1/27 | 163.1 | -2.6 | -4.6 | 161.7 | -8.3 | | 5.5 | -13.9 | -13.2 | 3.8 | -16.7 | -9.4 | 15.6 |
| JX1/62 x JX1/51 | -39.7 | -32.7 | -23.9 | -69.8 | -66.4 | | -61.9 | -90.2 | -68.2 | -58.2 | -90.5 | -66.8 | -53.5 |
| JX1/62 x JX1/54 | 0.0 | 79.4 | 66.9 | 0.0 | -10.3 | | -16.5 | 108.3 | -15.1 | -8.2 | 101.7 | -11.4 | 2.2 |
| JX1/62 x JX1/6 | 333.3 | 6.9 | 25.5 | 265.1 | -11.4 | | 12.9 | 18.6 | -16.2 | 24.1 | 14.9 | -12.5 | 38.2 |
| JX1/62 x JX1/7 | 205.4 | -9.5 | 11.3 | 119.7 | -11.9 | | 4.4 | 62.6 | -16.7 | 14.7 | 57.5 | -12.9 | 27.7 |
| JX1/63 x JX1/113 | 0.0 | 89.9 | 153.4 | 0.0 | -5.0 | | 26.8 | 48.9 | -15.0 | 8.7 | 44.2 | -11.2 | 20.9 |
| JX1/63 x JX1/23 | 540.6 | 99.4 | 192.8 | 220.4 | -0.3 | | 46.3 | 79.4 | -10.8 | 25.6 | 73.7 | -6.8 | 39.8 |
| JX1/63 x JX1/4 | - | 8.1 | 10.7 | - | 8.0 | | 8.3 | - | -3.2 | -2.9 | - | 1.1 | 8.1 |
| JX1/63 x JX1/6 | -4.6 | 17.2 | 32.7 | -33.4 | -0.5 | | 31.1 | -62.7 | -10.9 | 15.2 | -63.9 | -6.9 | 28.2 |
| JX1/63 xJX1/112 | 0.0 | 89.9 | 169.1 | 0.0 | -5.0 | | 34.7 | 38.3 | -15.0 | 15.4 | 33.9 | -11.2 | 28.5 |
| JX1/66 x JX1/23 | 831.4 | 123.1 | 226.4 | 365.7 | 11.6 | | 63.2 | 54.8 | -8.2 | 1.2 | 49.9 | -4.1 | 12.7 |
| JX1/66 x JX1/34 | 244.7 | -1.3 | 84.6 | 170.7 | -6.8 | | 73.1 | 57.8 | -13.6 | 22.7 | 52.8 | -9.8 | 36.5 |
| JX1/73 x JX1/23 | 688.1 | 107.4 | 177.9 | 294.1 | 3.7 | | 38.9 | 82.9 | -11.5 | 29.9 | 77.2 | -7.6 | 44.6 |
| JX1/73 x JX1/31 | 9.7 | 0.7 | 6.3 | -25.3 | 1.6 | | -0.2 | -3.9 | -12.1 | 6.3 | -6.9 | -8.2 | 18.3 |
| JX1/73 x JX1/36 | -100.0 | - | - | -100.0 | - | | - | -100 | - | - | -100.0 | - | - |
| JX1/73 x JX1/6 | 438.0 | 18.4 | 34.7 | 298.1 | 2.4 | | 39.1 | 84.9 | -12.7 | 22.3 | 79.0 | -8.8 | 36.1 |
| JX1/73 x JX1/63 | 181.6 | 3.7 | 34.7 | 157.6 | 1.2 | | 31.0 | 44.2 | -9.4 | 22.5 | 39.6 | -5.4 | 36.3 |
| JX1/74 x JX1/108 | - | 103.3 | 151.3 | - | 1.6 | | 25.6 | -72.0 | -11.7 | 1.2 | -72.9 | -7.8 | 12.7 |
| JX1/8 x JX1/112 | - | 93.3 | 73.7 | - | -3.4 | | 13.1 | 49.8 | 10.3 | -7.3 | 45.0 | -6.4 | 3.2 |
| JX1/8 x JX1/113 | - | 36.8 | 38.1 | - | -31.6 | | -30.9 | -29.3 | -36.5 | -26.2 | -31.5 | -33.7 | -17.9 |
| JX1/8 x JX1/118 | 135.2 | -3.8 | 10.2 | 102.2 | -4.4 | | 7.9 | 52.9 | -10.2 | 15.2 | 48.1 | -6.2 | 28.2 |
| JX1/8 x JX1/119 | 191.2 | -6.0 | 21.4 | 125.5 | -9.5 | | 4.7 | 70.6 | -9.3 | 11.7 | 65.2 | -5.3 | 24.4 |
| JX1/8 x JX1/23 | 211.1 | 96.4 | 64.2 | 55.6 | -1.8 | | -17.9 | 17.7 | -8.9 | -12.3 | 13.9 | -4.9 | -2.4 |
| JX1/83 x JX1/33 | - | 95.8 | 128.2 | - | -2.1 | | 14.7 | 49.1 | -10.4 | 16.9 | 44.4 | -6.4 | 30.1 |
| JX1/87 x JX1/118 | 0.0 | - | - | 0.0 | - | | - | -100 | - | - | -100.0 | - | - |
| JX1/87 x JX1/6 | - | 201.2 | 183.9 | - | 50.6 | | 42.0 | 34.7 | -6.3 | 24.9 | 30.4 | -2.2 | 38.9 |
| JX1/89 x JX1/63 | - | 99.7 | 170.3 | - | -0.1 | | 35.3 | -37.7 | -10.6 | 16.2 | -39.6 | -6.6 | 29.3 |
| JX1/90 x JX1/51 | 1527.3 | 197.1 | 265.7 | 714.7 | 48.6 | | 82.9 | 39.8 | -13.1 | 8.9 | 35.4 | -9.3 | 21.2 |
| JX1/98 x JX1/51 | - | - | - | - | - | | - | -30.1 | -4.4 | 12.1 | -32.3 | -0.2 | 24.8 |
| JX1/J 1 x JX1/33 | - | 88.8 | 94.9 | - | -5.6 | | -2.6 | - | -13.6 | -0.7 | - | -9.7 | 10.5 |
| JX1/J1 x JX1/ 59 | - | - | - | - | - | | - | 25.1 | -12.8 | 4.6 | 21.1 | -8.9 | 16.4 |
| JX1/J1 x JX1/ 90 | - | 233.2 | 248.7 | - | 66.6 | | 74.3 | -54.9 | -2.6 | 3.8 | -56.4 | 1.7 | 15.6 |
| JX1/J1 x JX1/23 | - | - | - | - | 0.0 | | 0.0 | 91.9 | -14.3 | 21.2 | 85.9 | -10.5 | 34.9 |
| JX1/J1 x JX1/51 | - | - | - | - | 0.0 | | 0.0 | 67.6 | -9.4 | 20.7 | 62.3 | -5.4 | 34.3 |
| JX1/J1 x JX1/66 | - | -13.2 | -25.2 | - | -56.6 | | -62.6 | -95.1 | -64.3 | -76.8 | -95.3 | -62.7 | -74.2 |
| JX1/J1 xJX1/6 | - | 160.2 | 186.2 | - | 30.1 | | 43.2 | - | -19.1 | 25.9 | - | -15.5 | 40.1 |
| JX1/JX1 x JX1/20 | - | 90.3 | 229.3 | - | -4.9 | | 64.8 | 21.9 | -16.5 | 8.3 | 18.1 | -12.8 | 20.6 |

MPH = Mid parent heterosis, BPH = Better parent heterosis, ECH1 = Economic heterosis in relation to standard variety 1 (GX1/46 × GX1/16), Economic heterosis in relation to standard variety 2 (JX1/5 × JX1/9).

**Supplementary Table S9. Soil chemical and physical properties**

|  | GX1 | | JX1 | | Bunso progeny | | | MX2 Tafo | |
| --- | --- | --- | --- | --- | --- | --- | --- | --- | --- |
| Property | 0-15cm depth | 15-30cm depth | 0-15cm depth | 15-30cm depth | 0-15cm depth | | 15-30cm depth | 0-15cm depth | 15-30cm depth |
| pH | 5.18 | 5.3 | 5.15 | 5.17 | 5.56 | | 5.46 | 5.59 | 5.75 |
| Electrical conductivity µScm-1 | 31.13 | 29.04 | 26.75 | 19.06 | 134.64 | | 81.78 | - | - |
| Organic C | 1.14 | 0.8 | 1.5 | 1.21 | 1.71 | | 1.06 | 0.96 | 0.81 |
| Total N | 0.07 | 0.05 | 0.1 | 0.08 | 0.11 | | 0.07 | 0.11 | 0.09 |
| Available P (mgkg-1) | 6.17 | 5.26 | 6.99 | 7.12 | 4.85 | | 4.76 | 24.98 | 20.49 |
| Exchangeable K (CmolcKg-1) | 0.08 | 0.07 | 0.11 | 0.09 | 0.16 | | 0.12 | 0.06 | 0.04 |
| Exchangeable Mg (CmolcKg-1) | 0.47 | 0.42 | 0.72 | 0.62 | 1.02 | | 0.67 | 1.05 | 0.73 |
| Exchangeable Ca (CmolcKg-1) | 1.33 | 1.38 | 1.69 | 1.68 | 2.07 | | 1.54 | 3.03 | 2.39 |
| Sand (%) | 72.57 | 68.24 | 44.35 | 50.8 | 42.07 | | 36.91 | 72.04 | 72.44 |
| Clay (%) | 12.09 | 13.09 | 30.32 | 27.87 | 28.26 | | 37.09 | 14.8 | 14.8 |
| Silt (%) | 15.33 | 18.67 | 25.33 | 21.33 | 29.67 | | 26 | 13.16 | 12.76 |
| Textural class (USDA standard) | Sandy loam | Sandy loam | clay loam | sandy clay loam | | Clay loam | Clay loam | Sandy loam | Sandy loam |

Supplementary Table S10. Year of establishment and number of accessions that constitute the four field gene banks of kola assessed in this study.

| Field gene bank | Year of establishment | Number of genotypes |
| --- | --- | --- |
| MX2 | 1987 | 32 |
| JX1 | 1986 | 90 |
| GX1 | 1984 | 84 |
| Bunso progeny | 1997 | 35 |
